# Covalent bidentate ligand-enabled regioselective Wacker-type oxidation of olefins†

**DOI:** 10.1039/d4ra07296k

**Published:** 2024-11-28

**Authors:** Liping Chen, Shuai Zhang, Yuchen Yang, Xue Wang, Wenjie Lan, Zhijie Chen, Wang Gong, Qingqing Nie, Wenqiang Cao, Ziyan Meng

**Affiliations:** a Ganzhou Polytechnic College Ganzhou 341000 Jiangxi province China mengzy0724@163.com; b Ganzhou People's Hospital Ganzhou 341000 Jiangxi province China

## Abstract

The utilization of Pd(ii)-catalyzed oxidation for the transformation of terminal olefins into methyl ketones has emerged as a particularly intriguing and versatile strategy in organic synthesis. Herein we report a novel Pd(ii)-catalyzed Wacker-type oxidation with covalent bidentate ligands. The ligand, 1-(pyridin-2-yl)-1,2-dihydro-3*H*-indazol-3-one, exhibits excellent performance in converting olefins to ketones. The optimized reaction conditions include the use of TBHP as oxidant, EtOH or MeCN as solvent and short reaction time. The substrate scope includes various substituted olefins, which undergo the desired oxidation reaction with high efficiency.

## Abstract 2

The utilization of Pd(ii)-catalyzed oxidation for the transformation of terminal olefins into methyl ketones has emerged as a particularly intriguing and versatile strategy in organic synthesis.

## Graphical abstract

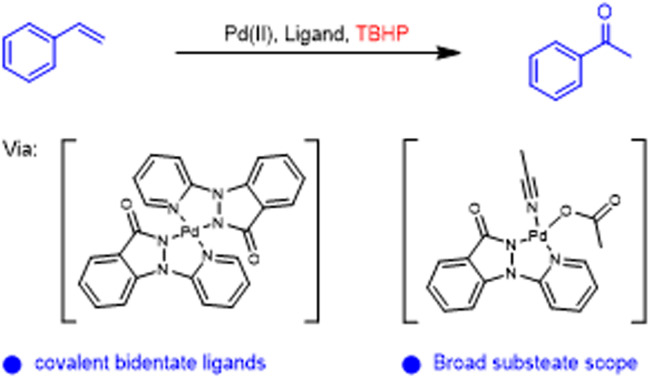

## Introduction

The oxidation of terminal olefins catalyzed by palladium(ii) to methyl ketones is a highly valuable chemical process, widely utilized in the synthesis of natural products and fine chemicals.^1^ Among these, the Wacker-type oxidation, typically facilitated by PdCl_2_ and CuCl_2_ under aerobic conditions, has garnered significant attention.^2^ However, the application of Wacker-type oxidation is hindered by limitations in the rate and selectivity of ketone and aldehyde product formation, particularly with olefins containing proximal heteroatoms.^3^

**Scheme 1:**
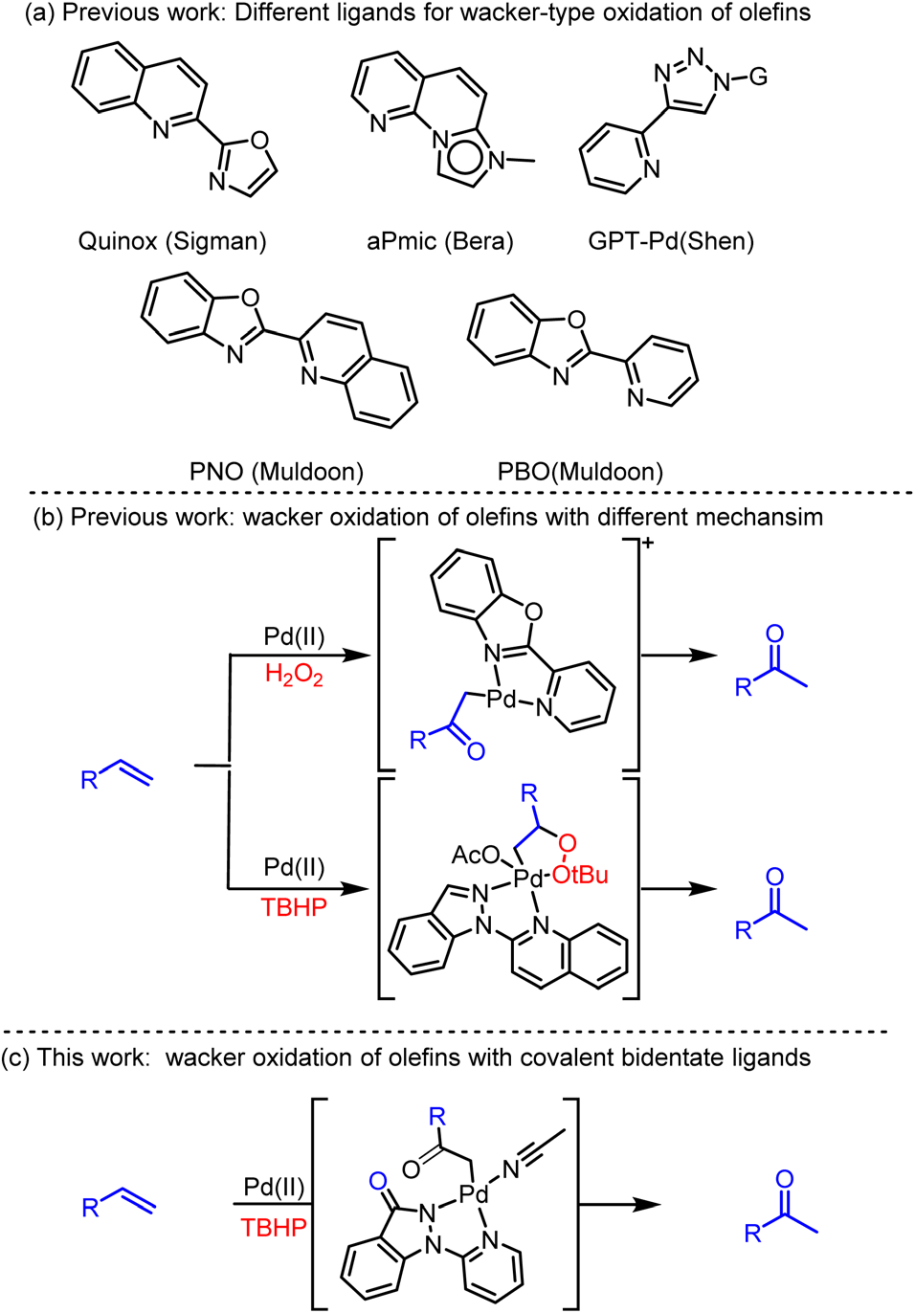
Oxidation of olefins with different Pd intermediates and ligands.

In 2009, Sigman's team innovatively introduced the tertiary butyl hydroperoxide (TBHP)-mediated Wacker-type oxidation method, in which they successfully demonstrated the high selectivity towards ketone products by utilizing Pd(ii) complexes containing the bidentate ligand quinoxaline-2-oxazoline (Quinox) (Scheme 1a).^4^ Building upon this foundation, they further reported the effective oxidation of various challenging substrates, such as protected allyl alcohols, allylamines, homoallyl alcohols, and internal olefins, into methyl ketone products.^5^ Subsequently, a novel TBHP-mediated Wacker-type oxidation method was also developed by Bera and colleagues, ingeniously utilizing fused pyridinyl-mesoionic carbenes (aPmic) as ligands (Scheme 1a).^6^ More recently, the glycosylpyridyltriazole palladium (GPT Pd) complex had emerged as an environmentally friendly catalyst for selective conversions in water.^7^

Notably, Muldoon's group pioneered the 2-(pyridin-2-yl)naphtho[1,2-*d*]oxazole (PNO) as the coupling ligand to achieve a Pd(ii)-catalyzed oxidation methodology, using O_2_ as the sole oxidant.^8^ Their subsequent studies devised innovative hydrogen peroxide (H_2_O_2_)-mediated Wacker-type oxidation utilizing a cationic palladium(ii) complex, [(PBO)Pd(NCMe)_2_][OTf]_2_, where PBO represents 2-(pyridin-2-yl)benzo[*d*]oxazole (Scheme 1a).^9^ To unravel the intricate catalytic oxidation mechanism, *in situ* high-resolution mass spectrometry analyses and exhaustive isotopic labeling experiments were undertaken (Scheme 1b).^10,11^ Their investigations revealed that he synthesis of methyl ketones was achieved *via* the formation of a Pd(ii)-enolate intermediate, followed by its subsequent protonolysis. Recently, Zou's team has reported an innovative TBHP-mediated, Pd(ii)-catalyzed, Wacker-type oxidation that utilizes 2-(1*H*-indazol-1-yl)quinolinone as ligand. This advanced methodology involves the acquisition of methyl ketones through a 1,2-hydride shift mechanism of the Pd(ii)-alkylperoxide complex.^11^

Herein we reported a TBHP-mediated Pd(ii)-catalyzed Wacker-type oxidation with covalent bidentate ligands (Scheme 1c). Although numerous related ligands have been reported and the catalytic efficiency is relatively high, the potential side reactions and the presence of impurities during the reaction process continue to pose significant challenges. The ligand, 1-(pyridin-2-yl)-1,2-dihydro-3*H*-indazol-3-one, features an electron-deficient covalent indazol-3-one moiety and electron-rich pyridin rings, enhancing the resilience of the catalytic system to harsh oxidative conditions. This Pd(ii) catalytic system exhibits exceptional performance in converting olefins to ketones, demonstrating remarkable versatility and stability even in the presence of interfering molecules such as Ac_2_O, MeOH, EtOH, NaOAc, and NaBr. To gain deeper insights into the intricate selective reaction mechanism, experimental studies were conducted, revealing that the dissociation of Pd(ii) intermediates and the subsequent regeneration of Pd(OAc)_2_ are primary contributors to impurity formation.

## Results and discussion

We began our studies by examining the oxidative progress of styrene (1a) in the presence of various of ligands (Table 1). A simple mixture of 1a with 5 mol% of Pd(OAc)_2,_ and 3.0 equivalents of TBHP(decan) in hexafluoroisopropanol (HFIP) at 45 °C for 12 h obtained acetophenone (2a) and benzaldehyde (3a), with an yield of 15% and 8%, respectively(Table 1). Moreover, 2-oxo-2-phenylethyl acetate (4a) could also be detected with a 6% yield in the presence of interfering ions AcO^−^ ions^.^. Interestingly, the assistance of 5 mol% of 1-(pyridin-2-yl)-1*H*-indazole (L1) resulted in the production of 2a, 3a and 4a with yields of 34%, 15% and 8%, respectively. Furthermore, covalent bidentate ligands (L2) could also enhance this transformation to 2a at higher rates, while simultaneously decreasing the rates towards 3a and 4a. Other covalent bidentate ligands, such as *N*-methoxypicolinamide (L3), failed to initiate this transformation, highlighting the importance of Pyridin-indazol-3-one in Pd-catalyzed Wacker-type oxidations. Notably, the two coordinating modules of this ligand exhibit distinct relative capabilities, with the indazol-3-one moiety forming a robust covalent bond, while the pyridyl unit functions as a σ-donor. Additionally, when quinolone rings (L4) or pyridine rings bearing different substituents (L5) were employed, the yields of 2a were inferior to those achieved with L2. Further ligands screening revealed that the presence of electron-withdrawing and electron-donating group on the benzene ring of indazol-3-one (L6-L8) could facilitating this reaction, albeit at lower rates. Consequently, 1-(pyridin-2-yl)-1,2-dihydro-3*H*-indazol-3-one (L2) was identified to be the optimal ligands for the Wacker-type oxidation of 1a.

**Table 1:** Ligand optimizations for the Wacker-type oxidation of olefins^a^^,^^b^

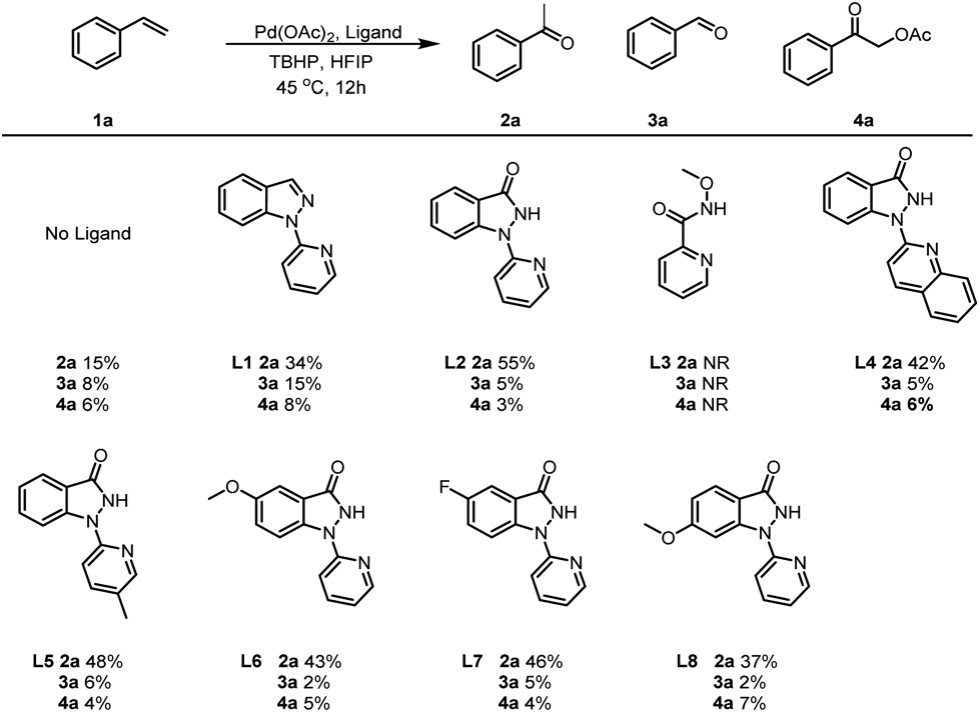

a The yields of 2a, 3a and 4a were determined by HPLC.

b Reaction conditions: 1a (1.0 mmol), Pd(OAc)_2_ (0.10 mmol), ligand (0.10 mmol), TBHP(decane) (3.0 mmol) and HFIP (6 mL) at 45 °C for 12 h.

With the optimal ligands in hand, we continued to meticulously screen the reaction conditions by testing a diverse array of reaction parameters, including oxidants, solvents, temperatures and reaction time(Table 2). Alternative peroxide oxidants, such as TBHP (water), H_2_O_2_, *m*-CPBA, PhI(OAc)_2_ and K_2_S_2_O_8_ also facilitated the reaction, with reduced yields of 2a (entries 2–6). Further solvent screening revealed that both EtOH and MeCN could also promote the reaction, with diminished transformation rates of 2a (entries 7–8). Notably, elevating the reaction temperature to 60 °C significantly enhanced the yield of 2a to 86% (entry 9). However, a subsequent increase in temperature to 75 °C led to a slight decrease in yield (entry 10), underscoring the crucial role of temperature optimization in achieving successful transformation. Meanwhile, increasing the quantity of L2 and reducing the reaction duration have negligible influence on the conversion efficiency to 2a, yet they contribute to diminishing the formation of impurities (entry 11). Further solvent screening showed that a mixed solvent of HFIP and MeCN was capable of increasing the yield of 2a and reducing the formation of impurities (entry 12). Furthermore, the incorporation of additional MeCN enables the similar yield of 2a with a reduced quantity of Pd(OAc)_2_ and L2 (entries 13–14). Oxidant equivalents were also investigated, with reduced yields of 2a using 1 eq. or 2eq. of oxidant (entries 15–16) and approximate yield use 5eq. of oxidant.

**Table 2:** Optimization of reaction conditions^a^^,^^b^

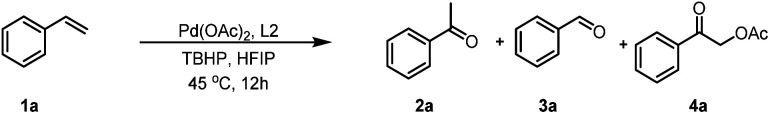					
Entry	Oxidant	Solvent	Temp (°C)	Time (h)	Yield^c^ (%)
1	TBHP	HFIP	45	12	55 : 5 : 3
2^c^	TBHP	HFIP	45	12	31 : 15 : 0
3	H_2_O_2_	HFIP	45	12	26 : 17 : 0
4	*m*-CPBA	HFIP	45	12	23 : 9 : 0
5	PhI(OAc)_2_	HFIP	45	12	11 : 0 : 0
6	K_2_S_2_O_8_	HFIP	45	12	35 : 21 : 0
7	TBHP	EtOH	45	12	21 : 7 : 10
8	TBHP	MeCN	45	12	39 : 6 : 16
9	TBHP	HFIP	60	12	86 : 4 : 6
10	TBHP	HFIP	75	12	82 : 5 : 8
11^d^	TBHP	HFIP	60	6	83 : 3 : 0
12^e^	TBHP	HFIP + MeCN	60	6	97 : 0 : 0
13^f^	**TBHP**	**HFIP** + **MeCN**	**60**	**6**	**95 : 0 : 0**
14^f^	TBHP	HFIP + MeCN	60	4	84 : 0 : 0
15^g^	TBHP	HFIP + MeCN	60	6	68 : 0 : 0
16^h^	TBHP	HFIP + MeCN	60	6	90 : 0 : 0
17^i^	TBHP	HFIP + MeCN	60	6	96 : 0 : 0

a For entries 1–14: reaction was conducted with 1a (1.0 mmol), Pd(OAc)_2_ (0.10 mmol), L2 (0.10 mmol), oxidant (3.0 mmol), and solvent (6 mL).

b The yields of 2a, 3a and 4a were determined by HPLC.

c For entry 2: TBHP(70% in water) (3.0 mmol).

d For entry 8: L2 (0.15 mmol).

e For entry 9: HFIP (5.5 mL) and MeCN (0.5 mL).

f For entries 13–14: reaction was conducted with 1a (1.0 mmol), Pd(OAc)_2_ (0.02 mmol), L2 (0.025 mmol), oxidant (3.0 mmol), HFIP (5.5 mL) and MeCN (0.5 mL).

g xidant (1.0 mmol).

h xidant (2.0 mmol).

i xidant (5.0 mmol).

With the optimized reaction conditions established, we embarked on investigating the olefin scope of the Wacker-type oxidation reaction (Table 3). Styrenes with diverse mono-substituents, such as fluorine, chlorine, bromine, trifluoromethyl, methyl, tertiary butyl, phenyl, methoxy, acetoxy, and phenoloxy groups, underwent the desired oxidation reaction with remarkable efficiency, yielding the products in excellent isolated yields ranging from 89% to 98% (2a–2k). Notably, styrenes featuring strong electron-withdrawing group or harboring reactive phenolichydroxyl and dimethylamino groups were compatible with the reaction conditions, smoothly affording the target products in moderate to good isolated yields (2l–2o). Furthermore, the presence of electron-withdrawing or electron-donating groups at the *meta* or *ortho* positions of styrenes still permitted the formation of the corresponding methyl ketones in good yields (2q–2u). 1,2,3,4,5-Pentafluoro-6-vinylbenzene remained inert under the reaction conditions, likely hindered by its highly electron-deficient benzene ring, resulting in no observable product formation (2v). Additionally, more sterically hindered naphthyl-substituted olefins furnished 1-(naphthalen-2-yl)ethan-1-one in a yield of 94% (2w). Remarkably, the oxidation of internal alkenes, such as 1,2-dihydronaphthalene and (*E*)-1,2-diphenylethene, yielded their respective ketones in moderate yields (2x, 2y). Moderate yields of the corresponding product 1-phenoxypropan-2-one was achieved (2aa). Intriguingly, 4-vinylaniline, methyl cinnamate and allyl(phenyl)sulfane failed to yield any product, presumably due to their heightened susceptibility to oxidation (2p, 2z, 2ab).

**Table 3:** Scope of Wacker-type oxidation of olefins^a^^,^^b^

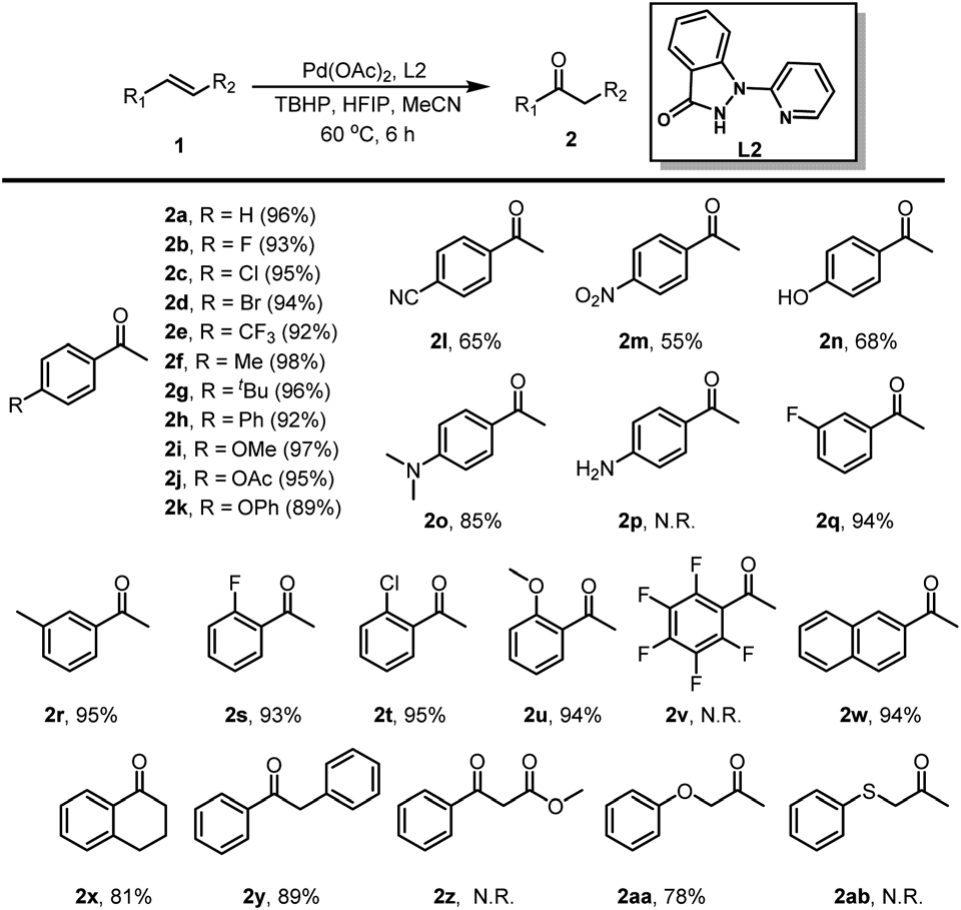

a Reaction was conducted with 1 (1.0 mmol), Pd(OAc)_2_ (0.02 mmol), L2 (0.025 mmol), TBHP(decane) (3.0 mmol), HFIP (5.5 mL) and MeCN (0.5 mL) at 60 °C for 12 h.

b Yields determined by HPLC.

**Scheme 2:**
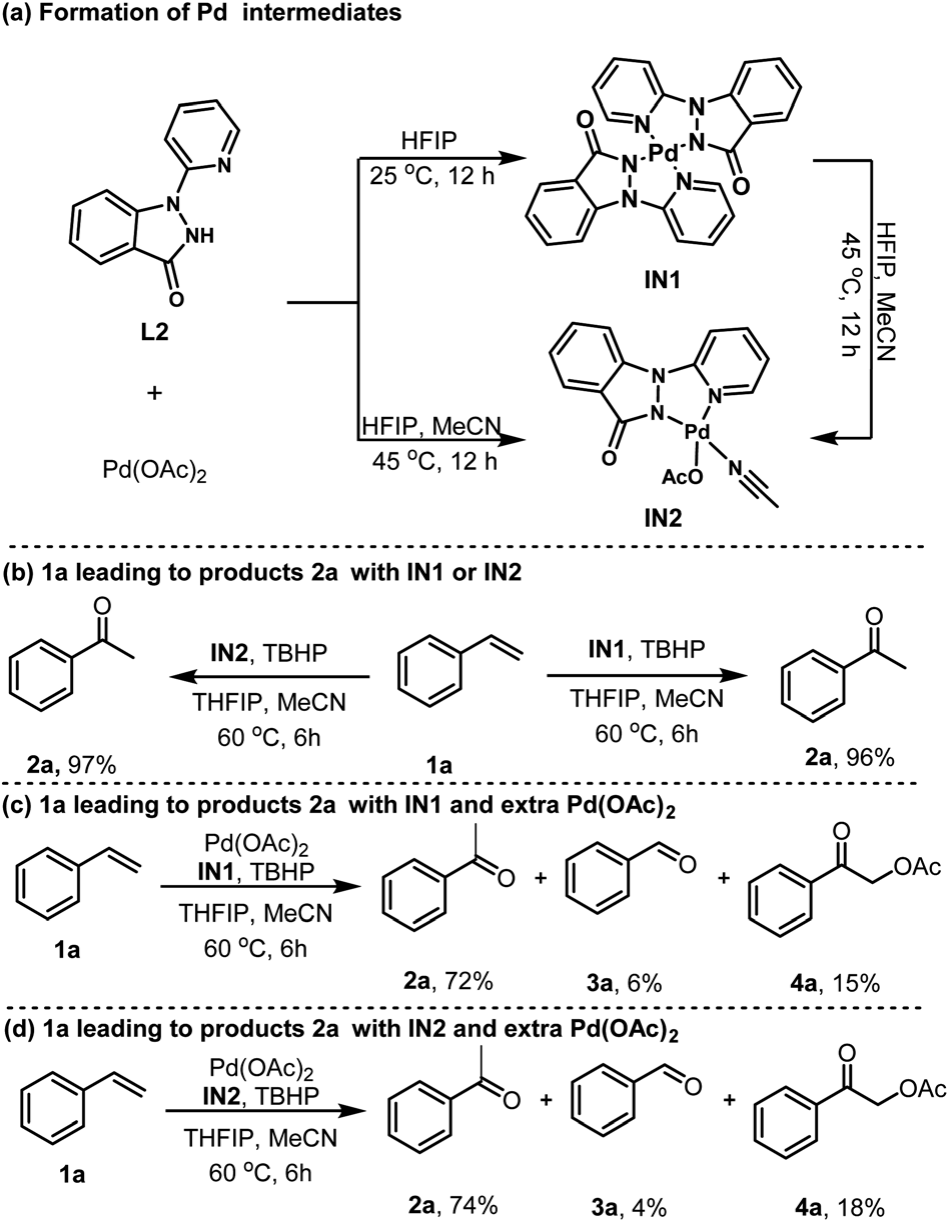
Mechanistic studies.

To gain insights into the reaction mechanism, we conducted a comprehensive mechanistic study. Initially, exposing L2 to Pd(OAc)_2_ in HFIP at ambient temperature yielded the corresponding dimeric Pd(ii) intermediate IN1 (Scheme 2a). Interestingly, substitution HFIP with MeCN/HFIP (8.3%, w/w) at 45 °C exclusively produced another monomer palladacycle IN2 under otherwise similar reaction conditions (Scheme 2a). Furthermore, IN1 could be easily converted into IN2 with the assistance of MeCN in HFIP. Additional experiments were conducted to substantiate the significance of these isolated intermediates. As anticipated, using a catalytic amount of IN1 or IN2 as a surrogate for Pd(OAc)_2_ successfully yielded 2a with yields of 97% and 96%, respectively (Scheme 2b). These observations highlight the role of IN1 and IN2 as activating intermediate in the synthesis of the targeted ketone products. To further elucidate the reaction mechanism, a series of studies were carried out by adding extra Pd(OAc)_2_. Specifically, the addition of extra Pd(OAc)_2_ beyond the amount required for IN1 or IN2 explicitly led to the formation of impurities 3a and 4a, suggesting that the presence of unbound Pd(OAc)_2_ in the system is the predominant factor contributing to the generation of these impurities (Scheme 2c and d).

**Scheme 3:**
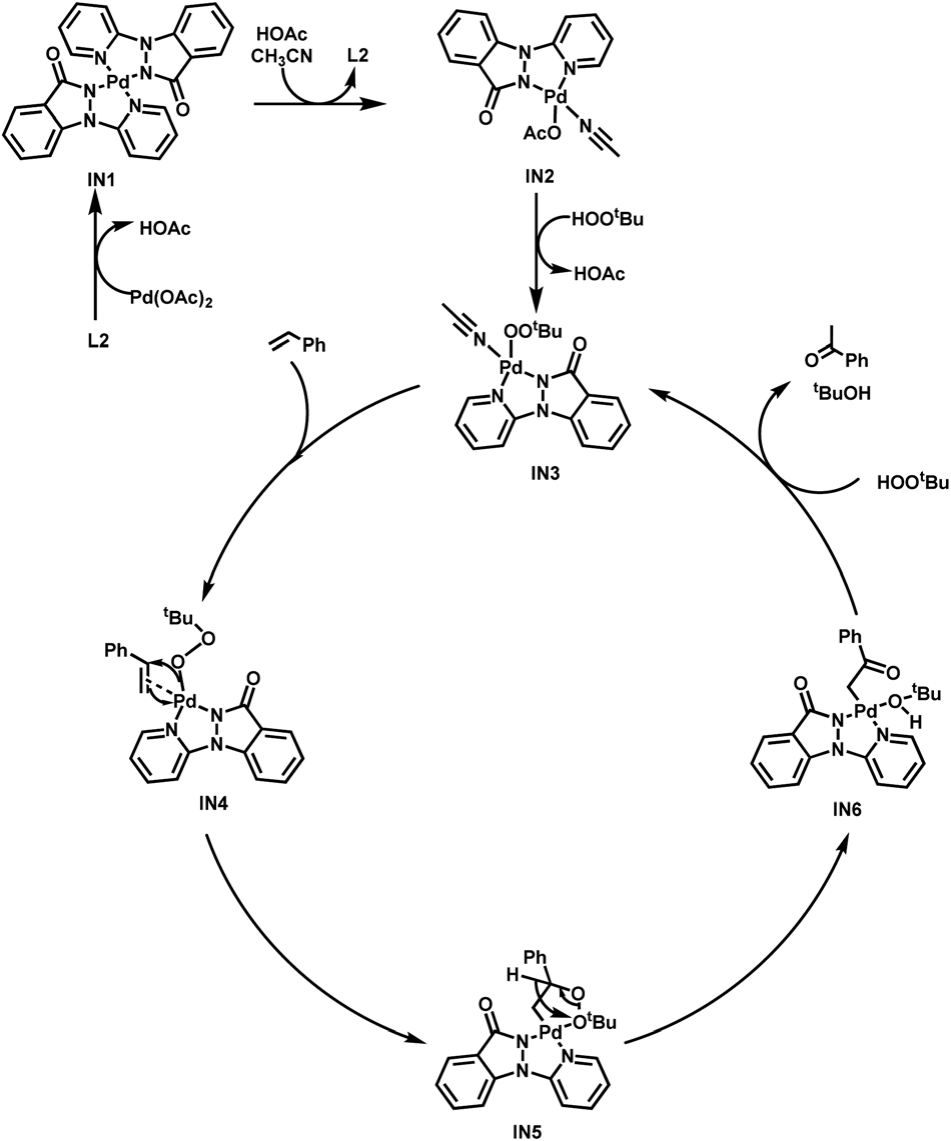
Proposed mechanism.

Based on the experimental results and previous research reports,^11^ a plausible mechanism is outlined here, utilizing 1a as an illustrative example (Scheme 3). The initial step involves the coordination of L2 with Pd(OAc)_2_, resulting in formation of the dimeric Pd(ii) intermediate IN1. Subsequently, the transition to the MeCN-coordinated monomer Pd(ii) intermediate IN2 is facilitated by the dissociation of the Pd–N (L2) interaction and the concurrent establishment of Pd–N (MeCN) and Pd–O (AcO^−^) bonds. Following this, a reaction with TBHP occurs, leading to the release of MeCN and the generation of the Pd–OOtBu intermediate IN3. It is postulated that the subsequent interaction of IN3 with styrene (1a) generates the alkylperoxide intermediate IN4, which undergoes an oxygen insertion at the Markovnikov position, yielding IN5. IN5 then undergoes hydrogen-atom abstraction of the α-H and homolysis of O–O bond, culminating in the production of intermediate IN6. Ultimately, the protonolysis of the novel Pd-enolate intermediate IN6 gives rise to acetophenone (2a) and the activating intermediate IN3, thereby completing the catalytic cycle.

## Conclusions

In conclusion, this study presents a novel and efficient method for the Wacker-type oxidation of olefins to methyl ketones using covalent bidentate ligands. The method exhibits remarkable versatility and stability even in the presence of interfering molecules such as Ac_2_O, MeOH, EtOH, NaOAc, and NaBr. The ligand identified in this study plays a crucial role in enhancing the resilience of the catalytic system to harsh oxidative conditions and promotes the conversion of olefins to ketones through a series of key intermediates. The resulting diverse ketones from the olefin demonstrate this protocol's potential for further structural manipulation of functional molecules.

## Data availability

The data supporting this article has been uploaded as part of the ESI.†

## Author contributions

L. Chen, S. Zhang, Y. Yang and X. Wang performed reaction experiments and syntheses of substrates, W. Lan, Z. Chen, W. Gong, revised the manuscript. Q. Nie and W. Cao helped to prepare the manuscript. Z. Meng supervised the project and prepared the manuscript. All authors discussed the results and commented on the manuscript.

## Conflicts of interest

The authors declare no competing financial interest.

## Supplementary Material

RA-014-D4RA07296K-s001
