# Supplementary material for: Covalent bidentate ligand-enabled regioselective Wacker-type oxidation of olefins
Source: RSC Adv. 2024 Nov 28;14(51):37928–32. doi: 10.1039/d4ra07296k (PMC11603338; doi:10.1039/d4ra07296k)
Supplement: RA-014-D4RA07296K-s001 [file RA-014-D4RA07296K-s001.pdf]

Supplementary Information (SI) for RSC Advances.
This journal is © The Royal Society of Chemistry 2024

# Supporting Information

# Covalent Bidentate Ligand-Enabled Regioselective Wacker-type Oxidation of Olefins

*Liping Chen[b], Shuai Zhang[a], Yuchen Yang[a], Xue Wang[a], Wenjie Lan[a], Zhijie Chen[a], wang Gong[a], qingqing Nie[a], Wenqiang Cao[a], ┴and Ziyan Meng[a*]*

[a] Ganzhou polytechnic college, Ganzhou, 341000, Jiangxi province, China

[b] Ganzhou People's Hospital, Ganzhou, 341000, Jiangxi province, China

*Corresponding author. Email: mengzy0724@163.com

# Table of Contents

1. General Information..........................................................................................S3

2. Experimental Section..........................................................................................S3

2.1 Ligands Preparation..........................................................................................S3

2.2 General Procedures for Ketone.........................................................................S4

3. Characterization Data..........................................................................................S5

4. Mechanistic Study..........................................................................................S13

4.1 Preparation of Intermediates..........................................................................S13

4.2 **1a** Leading to Products **2a** using **IN1** or **IN2**…...…..........................S15

4.3 **1a** leading to products **2a** with **IN1** and extra $Pd(OAc)_2$............................S15

4.4 **1a** leading to products **2a** with **IN2** and extra Pd(OAc)……...................…S16

5. References..........................................................................................S16

6. NMR Spectra..........................................................................................S17

# 1. General Information

Catalytic reactions were carried out in Schlenk tubes using pre-dried glassware. Various ligands were synthesized according to previously described procedures. Commercially available reagents were purchased from Energy Chemical, Bidepharm, Sigma Aldrich, Alfa Aesar, Acros or TCI, and used without purification unless otherwise noted. Column chromatography purification was performed using 200–300 mesh silica gel. NMR spectra were mostly recorded for $^{1}$H NMR at 500 MHz and for $^{13}$C NMR at 125 MHz. $CDCl_3$ and DMSO-D6 were used as solvents. Chemical shifts were referenced relative to residual solvent signal ($CDCl_3$: $^{1}$H NMR: δ 7.26 ppm, $^{13}$C NMR: δ 77.16 ppm; DMSO-D6: $^{1}$H NMR: δ 2.50 ppm, $^{13}$C NMR: δ 39.52 ppm). The following abbreviations are used to describe peak patterns where appropriate: br = broad, s = singlet, d = doublet, t = triplet, q = quartet, m = multiplet. Coupling constants (*J*) are reported in Hertz (Hz). HRMS was performed on Agilent Technologies 6224 TOF LC/MS apparatus (ESI).

# 2. Experimental Section

## 2.1 Ligands Preparation

### 2.1.1 Preparation of 1-(pyridin-2-yl)-1*H*-indazole (L1)[1]

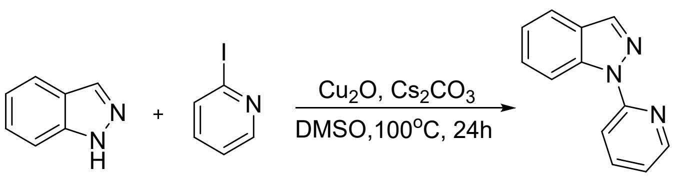


**L1**

A reaction tube (10 mL) with magnetic stir bar was charged with 1*H*-indazole (118 mg, 1.0 mmol), $Cu_2O$ (143 mg, 1.0 mmol), $Cs_2CO_3$ (750 mg, 2.0 mmol), and 2-iodopyridine (306 mg, 1.5 mmol). Then 1 mL DMSO was added to the reaction tube. The reaction system was stirred at 100°C oil bath for 24h. After cooling to room temperature, the mixture was diluted with dichloromethane and filtered through a pad of celite. The combined organic extracts were dried over anhydrous $Na_2SO_4$ and the solvent was removed under reduced pressure. The residue was purified by silica gel column chromatography (PET: EtOAc = 15:1) to afford **L1** as white solids (135 mg) in 69% yield.

### 2.1.2 Preparation of covalent bidentate ligands (L2, L4−L8)[2](Method 1)

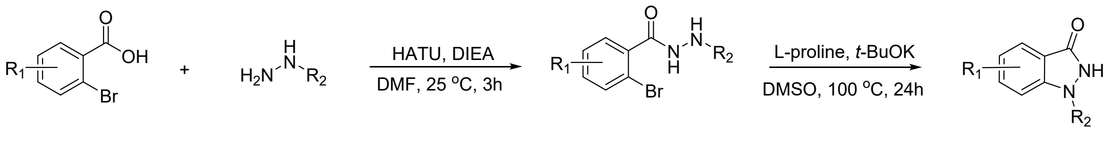


**S1** **S2** **S3** **L2, L4-L8**

Step 1：A reaction tube (100 mL) with magnetic stir bar was charged with benzoic acid derivative **S1** (10.0 mmol), HATU (15.0 mmol), DIPEA (15.0 mmol). Then 15 mL DMSO was added to the reaction tube. Subsequently, hydrazine derivatives **S2** (12.0 mmol) was added to the reaction

system. Then, the reaction system was stirred at 25 °C for 3 h. the reaction mixture was poured in 100 mL water and extracted with ethyl acetate (3 × 50 mL). The combined organic layers were washed with brine and dried over sodium sulphate. The solvent was removed under reduced pressure, and directly loaded onto silica gel for flash column chromatography (PET/EtOAc) to afford the desired products **S3**.

Step 2：A reaction tube (100 mL) with magnetic stir bar was charged with **S3 (**5.0 mmol**)**, L-proline (2.0 mmol, 40 mol %), and *t*-BuOK (10.0 mmol, 2.0 equiv) in DMSO (15 mL). The reaction system was stirred at 100 °C for 36 h. The reaction was quenched with a saturated $NaHCO_3$ solution, and the mixture was extracted with AcOEt. The organic layers were dried over sodium sulphate. The solvent was removed under reduced pressure, and directly loaded onto silica gel for flash column chromatography (PET/EtOAc) to afford the desired products **(L2, L4−L8)**.

**2.1.3 Preparation of covalent bidentate ligands (L3)**

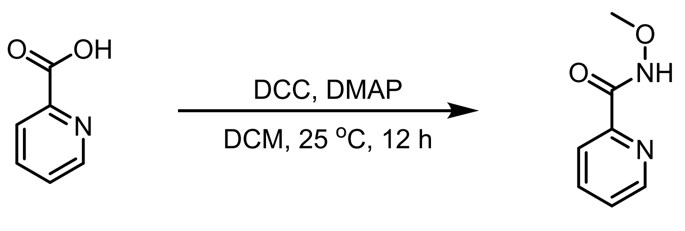


**S4** **L3**

A reaction tube (100 mL) with magnetic stir bar was charged with picolinic acid derivative **S4** (5.0 mmol), methoxyammonium chloride (625 mg, 7.5 mmol), dimethyllaminopropyl) carbodiimidehydrochloride (1440 mg, 7.5 mmol), and 4-dimethylaminopyridine (1830 mg, 15 mmol). Then 25 mL DCM was added to the reaction tube. Then, the reaction system was stirred at 25 °C for 12 h. The solvent was removed under reduced pressure, and the residue was purified by silica gel column chromatography (PET: EtOAc = 10:1) to afford **L3** as a white solid (1216.4 mg) in 80% yield.

## 2.2 General Procedures for Ketone (Method A)

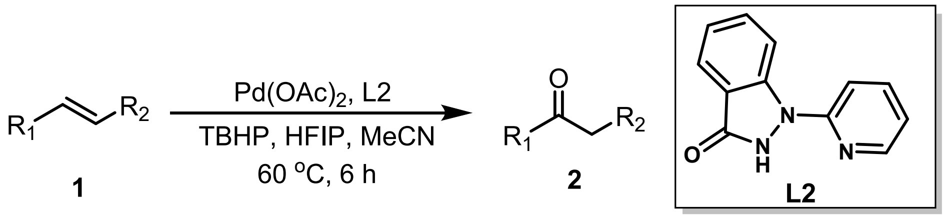


A reaction tube (10 mL) with magnetic stir bar was charged with olefins **1** (1.0 mmol), $Pd(OAc)_2$ (5.0 mg, 0.02 mmol), **L2** (5.0 mg, 0.025 mmol), TBHP (5 mmol/L in decane, 600 μL, 3.0 mmol), MeCN (0.5 mL)and HFIP (5.5 mL). The reaction was allowed to stir at 60°C oil bath for 6 h. After cooling to room temperature, the reaction mixture was evaporated to remove the solvent and

directly loaded onto silica gel for flash column chromatography (PET/EtOAc) to afford the desired products **2**.

# 3. Characterization Data

## 3.1 For ligand

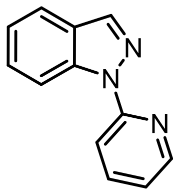

**1-(Pyridin-2-yl)-1H-indazole (L1):** $^{1}$H NMR (400 MHz, $CD_3OD$-$d_4$) $\delta$ 8.76 (dd, $J$ = 8.8, 1.2 Hz, 1H), 8.52 (dd, $J$ = 4.8, 1.2 Hz, 1H), 8.24 (s, 1H), 8.01 (d, $J$ = 8.8 Hz, 1H), 7.93 – 7.89 (m, 1H), 7.81 (d, $J$ = 8.0 Hz, 1H), 7.53 – 7.49 (m, 1H), 7.30 – 7.26 (m, 1H), 7.24 – 7.21 (m, 1H); $^{13}$C NMR (100 MHz, $CD_3OD$-$d_4$) $\delta$ 155.5, 148.9, 140.1, 139.8, 138.0, 129.0, 127.4, 123.7, 121.9, 121.4, 116.1, 114.5; HRMS (ESI) *m/z* calcd. for $C_{12}H_{10}N_3$ $[M+H]^+$ 196.0869, found 196.0872.

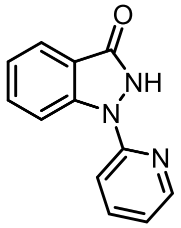

**1-(Pyridin-2-yl)-1,2-dihydro-3*H*-indazol-3-one (L2):** The title compound was obtained by column chromatography (PET: EtOAc = 10:1) as a white solid (1614.9 mg) in 73% yield according to the **Method 1**. $^{1}$H NMR (400 MHz, DMSO-$d_6$) $\delta$ 11.59 (s, 1H), 8.68 (d, $J$ = 8.5 Hz, 1H), 8.47 (dd, $J$ = 5.0, 1.8 Hz, 1H), 7.90 (ddd, $J$ = 8.9, 7.4, 1.9 Hz, 1H), 7.80 – 7.70 (m, 2H), 7.53 (ddd, $J$ = 8.4, 7.0, 1.2 Hz, 1H), 7.27 – 7.21 (m, 1H), 7.15 (ddd, $J$ = 7.3, 4.9, 1.1 Hz, 1H); $^{13}$C NMR (100 MHz, DMSO-$d_6$) $\delta$ 157.0, 153.8, 147.8, 139.4, 138.8, 128.9, 121.6, 120.1, 118.6, 116.0, 115.0, 111.5; HRMS (ESI) m/z calcd. for $C_{12}H_{10}N_3O$ $[M+H]^+$ 212.0818, found 212.0812.

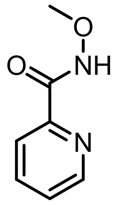

**1-Phenyl-1,2-dihydro-3*H*-indazol-3-one (L3):** $^{1}$H NMR (400 MHz, DMSO-$d_6$) $\delta$ 12.03 (s, 1H), 8.61 (dt, $J$ = 4.8, 1.4 Hz, 1H), 8.01 – 7.97 (m, 2H), 7.60 (p, $J$ = 4.4 Hz, 1H), 3.70 (s, 3H); $^{13}$C NMR (100 MHz, DMSO-$d_6$) $\delta$ 161.1, 149.6, 148.5, 137.8, 126.8, 122.1, 63.2; HRMS (ESI) m/z calcd. for $C_7H_9N_2O_2$ $[M+H]^+$ 153.0659, found 153.0653.

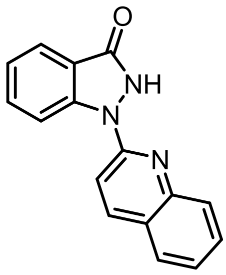

***N-M*ethoxyisoquinoline-1-carboxamide (L4):** The title compound was obtained by column chromatography (PET: EtOAc = 10:1) as a white solid (1932.1 mg) in 74% yield according to the **Method 1**. $^{1}$H NMR (400 MHz, DMSO-$d_6$) $\delta$ 11.74 (s, 1H), 9.06 (d, $J$ = 8.5 Hz, 1H), 8.43 (d, $J$ = 9.0 Hz, 1H), 8.01 (d, $J$ = 8.8 Hz, 2H), 7.94 (dd, $J$ = 8.1, 1.4 Hz, 1H), 7.83 (d, $J$ = 7.9 Hz, 1H), 7.76 (ddd, $J$ = 8.5, 6.9, 1.5 Hz, 1H), 7.64 (ddd, $J$ = 8.4, 6.9, 1.2 Hz, 1H), 7.53 – 7.47 (m, 1H), 7.31 (t, $J$ = 7.5 Hz, 1H); $^{13}$C NMR (100 MHz, DMSO-$d_6$) $\delta$ 157.3, 152.4, 146.3, 139.6, 138.8, 130.3, 129.3, 128.0, 127.4, 125.1, 124.9, 122.3, 120.3, 116.6, 115.7, 112.4; HRMS (ESI) m/z calcd. for $C_{16}H_{12}N_3O$ $[M+H]^+$ 262.0975, found 262.0969.

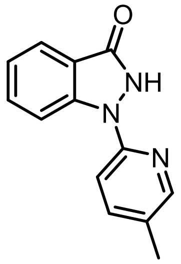

**1-(Quinolin-2-yl)-1,2-dihydro-3*H*-indazol-3-one (L5):** The title compound was obtained by column chromatography (PET: EtOAc = 10:1) as a white solid (1575.6 mg) in 70% yield according to the **Method 1**. $^{1}$H NMR (400 MHz, DMSO-$d_6$) $\delta$ 11.50 (s, 1H), 8.63 (d, $J$ = 8.6 Hz, 1H), 8.30 (d, $J$ = 2.2 Hz, 1H), 7.78 – 7.70 (m, 2H), 7.63 (d, $J$ = 8.5 Hz, 1H), 7.51 (ddd, $J$ = 8.4, 7.0, 1.2 Hz, 1H), 7.21 (t, $J$ = 7.5 Hz, 1H), 2.30 (s, 3H); $^{13}$C NMR (100 MHz, DMSO-$d_6$) $\delta$ 156.7, 151.9, 147.4, 139.3, 139.2, 128.7, 127.6, 121.3, 120.1, 115.7, 114.8, 111.2, 17.3; HRMS (ESI) m/z calcd. for $C_{13}H_{12}N_3O$ $[M+H]^+$ 226.0975, found 226.0969.

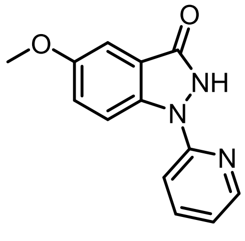

1-(5-Methylpyridin-2-yl)-1,2-dihydro-3*H*-indazol-3-one **(L6):** The title compound was obtained by column chromatography (PET: EtOAc = 10:1) as a white solid (1856.4 mg) in 77% yield according to the **Method 1**. $^{1}$H NMR (400 MHz, DMSO-$d_6$) $\delta$ 11.46 (s, 1H), 8.67 – 8.54 (m, 1H), 8.43 (dd, $J$ = 5.1, 1.8 Hz, 1H), 7.87 (ddd, $J$ = 8.9, 7.3, 1.9 Hz, 1H), 7.68 (d, $J$ = 8.4 Hz, 1H), 7.24 – 7.05 (m, 3H), 3.82 (s, 3H); $^{13}$C NMR (100 MHz, DMSO-$d_6$) $\delta$ 156.7, 154.6, 153.6, 147.7, 138.7, 134.9, 119.6, 118.3, 116.3, 116.1, 111.0, 100.2, 55.5; HRMS (ESI) m/z calcd. for $C_{13}H_{12}N_3O_2$ $[M+H]^+$ 242.0924, found 242.0918.

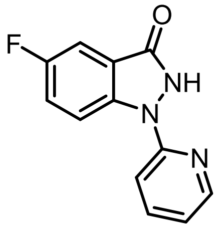

**1-(5-Chloropyridin-2-yl)-1,2-dihydro-3H-indazol-3-one (L7):** The title compound was obtained by column chromatography (PET: EtOAc = 10:1) as a white solid (1718.0 mg) in 75% yield according to the **Method 1**. $^{1}$H NMR (400 MHz, DMSO-$d_6$) $\delta$ 11.68 (s, 1H), 8.70 (dd, *J* = 9.2, 4.5 Hz, 1H), 8.46 (dd, *J* = 5.1, 1.8 Hz, 1H), 7.91 (ddd, *J* = 8.9, 7.3, 1.9 Hz, 1H), 7.72 (d, *J* = 8.4 Hz, 1H), 7.53 (dd, *J* = 8.4, 2.6 Hz, 1H), 7.43 (td, *J* = 9.2, 2.6 Hz, 1H), 7.17 (dd, *J* = 7.3, 4.9 Hz, 1H); $^{13}$C NMR (100 MHz, DMSO-$d_6$) $\delta$ 158.5, 156.6 (d, *J* = 4.8 Hz), 153.4, 147.7, 138.9, 136.3, 118.9, 117.8, 117.5, 116.6 (d, *J* = 8.8 Hz), 116.1 (d, *J* = 9.9 Hz), 111.3, 104.9, 104.6; HRMS (ESI) m/z calcd. for $C_{12}H_9FN_3O$ [M+H]$^+$ 230.0724, found 230.0718.

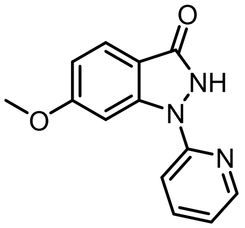

**5-Methoxy-1-(pyridin-2-yl)-1,2-dihydro-3*H*-indazol-3-one (L8):** The title compound was obtained by column chromatography (PET: EtOAc = 10:1) as a white solid (1880.4 mg) in 78% yield according to the **Method 1**. $^{1}$H NMR (400 MHz, DMSO-$d_6$) $\delta$ 11.44 (s, 1H), 8.47 (ddd, *J* = 4.9, 1.9, 0.8 Hz, 1H), 8.21 (d, *J* = 2.2 Hz, 1H), 7.89 (ddd, *J* = 9.0, 7.3, 1.9 Hz, 1H), 7.70 (dt, *J* = 8.5, 1.0 Hz, 1H), 7.63 (d, *J* = 8.7 Hz, 1H), 7.14 (ddd, *J* = 7.4, 4.9, 1.0 Hz, 1H), 6.86 (dd, *J* = 8.8, 2.2 Hz, 1H), 3.87 (s, 3H); $^{13}$C NMR (100 MHz, DMSO-$d_6$) $\delta$ 160.6, 156.9, 153.9, 147.7, 140.8, 138.7, 120.9, 118.5, 112.1, 111.5, 110.2, 97.3, 55.4; HRMS (ESI) m/z calcd. for $C_{13}H_{12}N_3O_2$ [M+H]$^+$ 242.0924, found 242.0918.

## 3.2 For Ketone Products

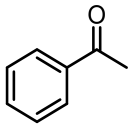

**Acetophenone (2a):** The title compound was obtained by column chromatography (PET: EtOAc = 20:1) as a colorless oil (115.2 mg) in 96% yield according to the **Method A**. $^{1}$H NMR (400 MHz, CDCl$_3$): $\delta$ 7.89−7.87 (m, 2H), 7.47 (t, *J* = 7.6 Hz, 1H), 7.39−7.35 (t, *J* = 7.6 Hz, 2H), 2.51 (s, 3H); $^{13}$C NMR (100 MHz, CDCl$_3$): $\delta$ 197.9, 137.0, 132.9, 128.4, 128.1, 26.4; HRMS (ESI) *m/z* calcd. for $C_8H_9O$ [M+H]$^+$ 121.0648, found 121.0642.

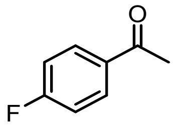

**1-(4-Fluorophenyl)ethan-1-one (2b):** The title compound was obtained by column chromatography (PET: EtOAc = 20:1) as a colorless oil (128.4 mg) in 93% yield according to the **Method A**. $^1$H NMR (400 MHz, $CDCl_3$): $\delta$ 8.01 – 7.90 (m, 2H), 7.15 – 7.04 (m, 2H), 2.56 (s, 3H); $^{13}$C NMR (100 MHz, $CDCl_3$): $\delta$ 196.6, 165.8 (d, $J$ = 253.0 Hz), 133.6 (d, $J$ = 3.0 Hz), 131.0 (d, $J$ = 9.2 Hz), 115.7 (d, $J$ = 21.8 Hz), 26.6; HRMS (ESI) m/z calcd. for $C_8H_8FO$ $[M+H]^+$ 139.0554, found 139.0548.

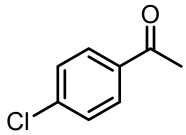

**1-(4-Chlorophenyl)ethan-1-one (2c):** The title compound was obtained by column chromatography (PET: EtOAc = 20:1) as a colorless oil (146.3 mg) in 95% yield according to the **Method A**. $^1$H NMR (400 MHz, $CDCl_3$): $\delta$ 7.86 (d, $J$ = 8.5 Hz, 2H), 7.39 (d, $J$ = 8.6 Hz, 2H), 2.55 (s, 3H); $^{13}$C NMR (100 MHz, $CDCl_3$): $\delta$ 196.9, 139.6, 135.5, 129.8, 128.9, 26.6; HRMS (ESI) m/z calcd. for $C_8H_8ClO$ $[M+H]^+$ 155.0258, found 155.0252.

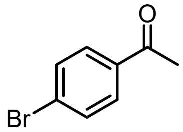

1-**(4-Bromophenyl)ethan-1-one (2d):** The title compound was obtained by column chromatography (PET: EtOAc = 20:1) as a colorless oil (186.1 mg) in 94% yield according to the **Method A**. $^1$H NMR (400 MHz, $CDCl_3$): $\delta$ 7.86 – 7.75 (m, 2H), 7.65 – 7.53 (m, 2H), 2.58 (s, 3H); $^{13}$C NMR (100 MHz, $CDCl_3$): $\delta$ 197.2, 135.9, 132.0, 130.0, 128.4, 26.7; HRMS (ESI) m/z calcd. for $C_8H_8BrO$ $[M+H]^+$ 198.9753, found 198.9747.

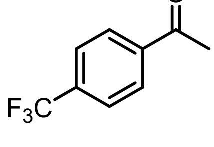

**1-(4-(Trifluoromethyl)phenyl)ethan-1-one (2e):** The title compound was obtained by column chromatography (PET: EtOAc = 20:1) as a colorless oil (173.0 mg) in 92% yield according to the **Method A**. $^1$H NMR (400 MHz, $CDCl_3$): $\delta$ 8.05 (d, $J$ = 8.1 Hz, 2H), 7.71 (d, $J$ = 8.1 Hz, 2H), 2.63 (s, 3H); $^{13}$C NMR (100 MHz, $CDCl_3$): $\delta$ 197.1, 139.8, 134.5 (q, $J$ = 32.6 Hz), 128.7, 125.8 (q, $J$ = 4.0 Hz), 125.8 (q, $J$ = 271.1 Hz), 26.6; HRMS (ESI) m/z calcd. for $C_9H_8F_3O$ $[M+H]^+$ 189,0522, found 189.0516.

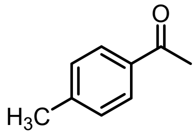

**1-(p-Tolyl)ethan-1-one (2f):** The title compound was obtained by column chromatography (PET: EtOAc = 20:1) as a colorless oil (131.4 mg) in 98% yield according to the **Method A**. $^1$H NMR (400 MHz, $CDCl_3$): $\delta$ 7.80 (d, $J$ = 7.9 Hz, 2H), 7.19 (d, $J$ = 7.9 Hz, 2H), 2.50 (s, 3H), 2.34 (s, 3H); $^{13}$C NMR (100 MHz, $CDCl_3$): $\delta$ 197.7, 143.7, 134.6, 129.1, 128.3, 26.4, 21.5; HRMS (ESI) m/z calcd. for $C_9H_{11}O$ $[M+H]^+$ 135.0848, found 135.0842.

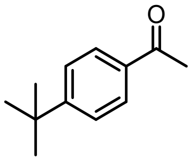


**1-(4-(*tert*-Butyl)phenyl)ethan-1-one (2g):** The title compound was obtained by column chromatography (PET: EtOAc = 20:1) as a colorless oil (169.1 mg) in 96% yield according to the **Method A**. $^1$H NMR (400 MHz, $CDCl_3$): $\delta$ 7.92 – 7.82 (m, 2H), 7.51 – 7.39 (m, 2H), 2.55 (s, 3H), 1.31 (s, 9H); $^{13}$C NMR (100 MHz, $CDCl_3$): $\delta$ 197.7, 156.7, 134.6, 128.3, 125.5, 35.0, 31.0, 26.5; HRMS (ESI) m/z calcd. for $C_{12}H_{17}O$ $[M+H]^+$ 177.1274, found 177.1268.

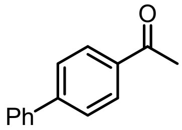


**1-([1,1'-Biphenyl]-4-yl)ethan-1-one (2h):** The title compound was obtained by column chromatography (PET: EtOAc = 20:1) as a white solid (180.4 mg) in 92% yield according to the **Method A**. $^1$H NMR (400 MHz, $CDCl_3$): $\delta$ 8.08 – 8.01 (m, 2H), 7.72 – 7.66 (m, 2H), 7.66 – 7.60 (m, 2H), 7.53 – 7.44 (m, 2H), 7.43 – 7.37 (m, 1H), 2.64 (s, 3H); $^{13}$C NMR (100 MHz, $CDCl_3$): $\delta$ 197.9, 145.9, 140.0, 136.0, 129.1, 129.0, 128.4, 127.4, 127.3, 26.8; HRMS (ESI) m/z calcd. for $C_{17}H_{13}O$ $[M+H]^+$ 197.0961, found 197.0955.

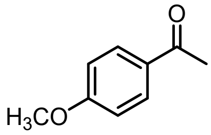


1-**(4-Methoxyphenyl)ethan-1-one (2i):** The title compound was obtained by column chromatography (PET: EtOAc = 20:1) as a colorless oil (145.6 mg) in 97% yield according to the **Method A**. $^1$H NMR (400 MHz, $CDCl_3$): $\delta$ 7.88 (d, $J$ = 8.5 Hz, 2H), 6.88 (d, $J$ = 8.5 Hz, 2H), 3.81 (s, 3H), 2.50 (d, $J$ = 1.2 Hz, 3H); $^{13}$C NMR (100 MHz, $CDCl_3$) $\delta$ 196.8, 163.5, 130.6, 130.3, 113.7, 55.4, 26.3; HRMS (ESI) m/z calcd. for $C_9H_{11}O_2$ $[M+H]^+$ 151.0754, found 151.0748.

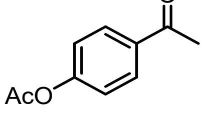


**4-Acetylphenyl acetate (2j):** The title compound was obtained by column chromatography (PET: EtOAc = 20:1) as a colorless oil (169.2 mg) in 95% yield according to the **Method A**. $^1$H NMR (400 MHz, CDCl3): $\delta$ 7.98 (dd, $J$ = 9.0, 2.4 Hz, 2H), 7.18 (dd, $J$ = 9.0, 2.4 Hz, 2H), 2.59 (s, 3H),

2.32 (s, 3H); $^{13}$C NMR (100 MHz, $CDCl_3$): $\delta$ 197.1, 169.0, 154.5, 134.8, 130.1, 121.9, 26.7, 21.2; RMS (ESI) m/z calcd. for $C_{10}H_{11}O_3$ $[M+H]^+$ 179.0703, found 179.0697.

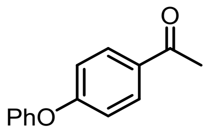


**1-(4-Phenoxyphenyl)ethan-1-one (2k):** The title compound was obtained by column chromatography (PET: EtOAc = 20:1) as a light yellow solid (188.8 mg) in 89% yield according to the **Method A**. $^1$H NMR (400 MHz, $CDCl_3$): $\delta$ 7.99 – 7.89 (m, 2H), 7.39 (ddd, $J$ = 9.8, 6.0, 2.2 Hz, 2H), 7.24 – 7.16 (m, 1H), 7.12 – 7.04 (m, 2H), 7.03 – 6.95 (m, 2H), 2.57 (s, 3H); $^{13}$C NMR (100 MHz, $CDCl_3$): $\delta$ 196.9, 162.1, 155.6, 132.0, 130.7, 130.2, 124.7, 120.3, 117.4, 26.5; RMS (ESI) m/z calcd. for $C_{14}H_{13}O_2$ $[M+H]^+$ 213.0910, found 213.0904.

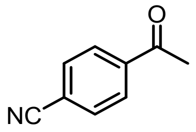


**4-acetylbenzonitrile (2l):** The title compound was obtained by column chromatography (PET: EtOAc = 20:1) as a light yellow solid (94.3 mg) in 65% yield according to the **Method A**. $^1$H NMR (400 MHz, $CDCl_3$) $\delta$ 8.06 – 8.01 (m, 2H), 7.81 – 7.75 (m, 2H), 2.64 (s, 3H; $^{13}$C NMR (100 MHz, CDCl3) $\delta$ 196.7, 140.1, 132.7, 128.8, 118.1, 116.6, 26.9; RMS (ESI) m/z calcd. for $C_9H_8NO$ $[M+H]^+$ 146.0600, found 146.0594.

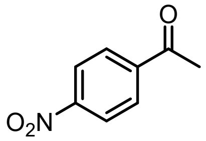


**1-(4-Nitrophenyl)ethan-1-one (2m):** The title compound was obtained by column chromatography (PET: EtOAc = 20:1) as a yellow solid (90.8 mg) in 55% yield according to the **Method A**. $^1$H NMR (400 MHz, $CDCl_3$): $\delta$ 8.36 – 8.24 (m, 2H), 8.17 – 8.03 (m, 2H), 2.67 (s, 3H); $^{13}$C NMR (100 MHz, $CDCl_3$): $\delta$ 196.5, 150.5, 141.5, 129.4, 124.0, 27.1; RMS (ESI) m/z calcd. for $C_8H_8NO_3$ $[M+H]^+$ 166.0499, found 166.0493

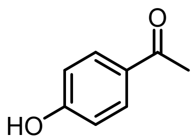


**1-(4-Hydroxyphenyl)ethan-1-one (2n):** The title compound was obtained by column chromatography (PET: EtOAc = 20:1) as a colorless oil (92.5 mg) in 68% yield according to the **Method A**. $^1$H NMR (400 MHz, $CDCl_3$): $\delta$ 7.99 (s, 1H), 7.94 – 7.87 (m, 2H), 6.98 – 6.91 (m, 2H), 2.58 (s, 3H); $^{13}$C NMR (100 MHz, $CDCl_3$): $\delta$ 198.8, 161.6, 131.4, 129.6, 115.7, 26.4; RMS (ESI) m/z calcd. for $C_8H_9O_2$ $[M+H]^+$ 139.0597, found 137.0591.

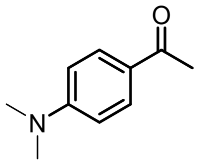

**1-(4-(Dimethylamino)phenyl)ethan-1-one (2o):** The title compound was obtained by column chromatography (PET: EtOAc = 20:1) as a colorless oil (138.6 mg) in 85% yield according to the **Method A**. $^1$H NMR (400 MHz, $CDCl_3$) $\delta$ 7.92 – 7.82 (m, 2H), 6.69 – 6.59 (m, 2H), 3.05 (s, 6H), 2.50 (s, 3H); $^{13}$C NMR (100 MHz, $CDCl_3$) $\delta$ 196.6, 153.5, 130.6, 125.4, 110.7, 40.1, 26.1; RMS (ESI) m/z calcd. for $C_{10}H_{14}NO$ $[M+H]^+$ 164.1070, found 164.1064.

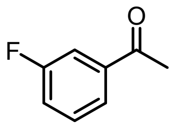

**1-(3-Fluorophenyl)ethan-1-one (2q):** The title compound was obtained by column chromatography (PET: EtOAc = 20:1) as a colorless oil (129.8 mg) in 94% yield according to the **Method A**. $^1$H NMR (400 MHz, $CDCl_3$): $\delta$ 7.66 (dt, $J$ = 7.7, 1.4 Hz, 1H), 7.55 (ddd, $J$ = 9.5, 2.8, 1.6 Hz, 1H), 7.37 (td, $J$ = 7.9, 5.4 Hz, 1H), 7.18 (tdd, $J$ = 8.2, 2.7, 1.1 Hz, 1H), 2.52 (s, 3H); $^{13}$C NMR (100 MHz, $CDCl_3$): $\delta$ 196.7 (d, $J$ = 2.1 Hz), 162.7 (d, $J$ = 246.1 Hz), 139.1 (d, $J$ = 6.1 Hz), 130.2 (d, $J$ = 7.6 Hz), 124.1 (d, $J$ = 2.9 Hz), 120.0 (d, $J$ = 21.5 Hz), 114.8 (d, $J$ = 22.2 Hz), 26.6; RMS (ESI) m/z calcd. for $C_8H_8FO$ $[M+H]^+$ 139.0554, found 139.0548.

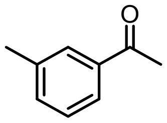

**1-(*m*-Tolyl)ethan-1-one (2r):** The title compound was obtained by column chromatography (PET: EtOAc = 20:1) as a colorless oil (127.4 mg) in 95% yield according to the **Method A**. $^1$H NMR (400 MHz, $CDCl_3$): $\delta$ 7.79 – 7.68 (m, 2H), 7.37 – 7.27 (m, 2H), 2.55 (s, 3H), 2.37 (s, 3H); $^{13}$C NMR (100 MHz, $CDCl_3$): $\delta$ 198.4, 138.3, 137.1, 133.9, 128.8, 128.4, 125.6, 26.6, 21.3; RMS (ESI) m/z calcd. for $C_9H_{11}O$ $[M+H]^+$ 135.0804, found 135.0798

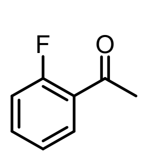

**1-(2-Fluorophenyl)ethan-1-one (2s):** TThe title compound was obtained by column chromatography (PET: EtOAc = 20:1) as a colorless oil (128.4 mg) in 93% yield according to the **Method A**. $^1$H NMR (400 MHz, $CDCl_3$): $\delta$ 7.77 (td, $J$ = 7.7, 2.0 Hz, 1H), 7.42 (dddd, $J$ = 9.0, 7.2, 5.0, 1.9 Hz, 1H), 7.12 (td, $J$ = 7.6, 1.2 Hz, 1H), 7.03 (ddd, $J$ = 11.3, 8.3, 1.1 Hz, 1H), 2.54 (d, $J$ = 4.9 Hz, 3H); $^{13}$C NMR (100 MHz, $CDCl_3$): $\delta$ 195.6 (d, $J$ = 3.3 Hz), 162.1 (d, $J$ = 253.2 Hz), 134.6 (d, $J$ = 9.0 Hz), 130.4 (d, $J$ = 2.5 Hz), 125.5 (d, $J$ = 12.8 Hz), 124.3 (d, $J$ = 3.7 Hz), 116.5 (d, $J$ = 21.7 Hz), 31.2 (d, $J$ = 7.3 Hz); RMS (ESI) m/z calcd. for $C_8H_8FO$ $[M+H]^+$ 139.0554, found 139.0548.

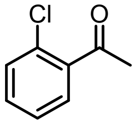

**1-(2-Chlorophenyl)ethan-1-one (2t):** The title compound was obtained by column chromatography (PET: EtOAc = 20:1) as a colorless oil (146.3 mg) in 95% yield according to the **Method A**. $^{1}$H NMR (400 MHz, $CDCl_3$): $\delta$ 7.51 (dd, $J$ = 7.6, 1.6 Hz, 1H), 7.39 – 7.31 (m, 2H), 7.28 (ddd, $J$ = 8.4, 6.7, 2.2 Hz, 1H), 2.60 (s, 3H); $^{13}$C NMR (100 MHz, $CDCl_3$): $\delta$ 200.3, 139.0, 132.0, 131.1, 130.6, 129.3, 126.9, 30.6; RMS (ESI) m/z calcd. for $C_8H_8ClO$ $[M+H]^+$ 155.0258, found 139.0252.

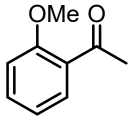

**1-(2-Methoxyphenyl)ethan-1-one (2u):** The title compound was obtained by column chromatography (PET: EtOAc = 20:1) as a colorless oil (141.1 mg) in 94% yield according to the **Method A**. $^{1}$H NMR (400 MHz, $CDCl_3$): $\delta$ 7.69 (dd, $J$ = 7.7, 1.8 Hz, 1H), 7.42 (ddd, $J$ = 8.7, 7.3, 1.8 Hz, 1H), 7.10 – 6.84 (m, 2H), 3.86 (s, 3H), 2.57 (d, $J$ = 1.2 Hz, 3H); $^{13}$C NMR (100 MHz, $CDCl_3$): $\delta$ 199.8, 158.9, 133.7, 130.3, 128.1, 120.5, 111.6, 55.4, 31.8; RMS (ESI) m/z calcd. for $C_9H_{11}O_2$ $[M+H]^+$ 151.0754, found 151.0748.

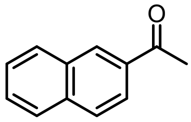

1-**(Naphthalen-2-yl)ethan-1-one (2w):** The title compound was obtained by column chromatography (PET: EtOAc = 20:1) as a white solid (159.9 mg) in 94% yield according to the **Method A**. $^{1}$H NMR (400 MHz, $CDCl_3$) $\delta$ 8.49 – 8.45 (m, 1H), 8.03 (dd, $J$ = 8.6, 1.8 Hz, 1H), 7.99 – 7.95 (m, 1H), 7.92 – 7.86 (m, 2H), 7.58 (dddd, $J$ = 19.5, 8.2, 6.9, 1.4 Hz, 2H), 2.73 (s, 3H). $^{13}$C NMR (100 MHz, $CDCl_3$) $\delta$ 198.3, 135.7, 134.6, 132.6, 130.3, 129.7, 128.6, 128.6, 127.9, 126.9, 124.0, 26.8; RMS (ESI) m/z calcd. for $C_{12}H_{11}O$ $[M+H]^+$ 171.0804, found 171.0798.

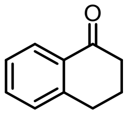

**3,4-Dihydronaphthalen-1-(2*H*)-one (2x):** The title compound was obtained by column chromatography (PET: EtOAc = 20:1) as a brown liquid (118.3 mg) in 81% yield according to the **Method A**. $^{1}$H NMR (400 MHz, $CDCl_3$) $\delta$ 8.06 (dd, $J$ = 7.9, 1.5 Hz, 1H), 7.49 (td, $J$ = 7.5, 1.5 Hz, 1H), 7.36 – 7.25 (m, 2H), 2.99 (t, $J$ = 6.1 Hz, 2H), 2.68 (dd, $J$ = 7.2, 5.8 Hz, 2H), 2.17 (p, $J$ = 6.3 Hz, 2H); 13C NMR (100 MHz, $CDCl_3$) $\delta$ 198.5, 144.6, 133.5, 132.7, 128.9, 127.2, 126.7, 39.2, 29.8, 23.4; RMS (ESI) m/z calcd. for $C_{10}H_{11}O$ $[M+H]^+$ 147.0804, found 147.0798.

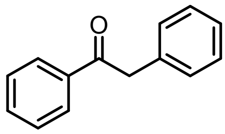

**1,2-Diphenylethan-1-one (2y):** The title compound was obtained by column chromatography (PET: EtOAc = 20:1) as a white solid (174.5 mg) in 89% yield according to the **Method A**. $^1$H NMR (400 MHz, $CDCl_3$) $\delta$ 8.08 – 8.04 (m, 2H), 7.63 – 7.57 (m, 1H), 7.53 – 7.47 (m, 2H), 7.40 – 7.34 (m, 2H), 7.31 (d, $J$ = 8.6 Hz, 3H), 4.33 (s, 2H); $^{13}$C NMR (100 MHz, $CDCl_3$) $\delta$ 197.8, 136.7, 134.7, 133.3, 129.6, 128.8, 128.8, 128.8, 127.0, 45.6; RMS (ESI) m/z calcd. for $C_{14}H_{13}O$ $[M+H]^+$ 197.0961, found 197.0955.

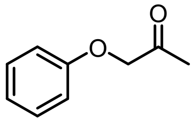

**1-Phenoxypropan-2-one (2aa):** The title compound was obtained by column chromatography (PET: EtOAc = 20:1) as a colorless oil (117.0 mg) in 78% yield according to the **Method A**. $^1$H NMR (400 MHz, $CDCl_3$) $\delta$ 7.33 – 7.27 (m, 2H), 7.00 (tt, $J$ = 7.4, 1.1 Hz, 1H), 6.91 – 6.86 (m, 2H), 4.53 (s, 2H), 2.28 (s, 3H); $^{13}$C NMR (100 MHz, $CDCl_3$) $\delta$ 206.1, 157.8, 129.8, 121.8, 114.6, 73.1, 26.7; RMS (ESI) m/z calcd. for $C_9H_{11}O_2$ $[M+H]^+$ 151.0754, found 151.0748.

# 4. Mechanistic Study

## 4.1 Preparation of Intermediates

### 4.1.1 Preparation of IN1

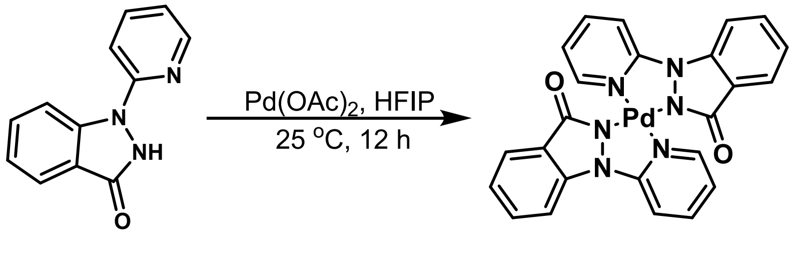


A reaction tube (10 mL) with magnetic stir bar was charged with 1-(Pyridin-2-yl)-1,2-dihydro-3*H*-indazol-3-one **L2** (105 mg, 0.50 mmol), $Pd(OAc)_2$ (47 mg, 0.20 mmol), and HFIP (5 mL). The reaction was allowed to stir at room temperature for 12 h. Upon completion, the reaction mixture was evaporated to remove the solvent and the solid obtained was washed with ether to remove excess **L2**. Analytically pure intermediate **IN1** was obtained yellow solids (55 mg) in 52% yield by recrystallization using DCM/EA at room temperature. **IN1** was then used to perform NMR.

$^1$H NMR (400 MHz,DMSO-$d_6$): 9.67 (dd, $J$ = 6.0, 1.2 Hz, 2H), 8.11−8.06 (m, 4H), 7.95 (d, $J$ = 7.6 Hz, 2H), 7.78 (d, $J$ = 7.6 Hz, 2H), 7.68 (t, $J$ = 8.0 Hz, 2H), 7.37 (t, $J$ = 7.6 Hz, 2H), 7.08 (t, $J$ = 7.2 Hz, 2H); $^{13}$C NMR (100 MHz, $CDCl_3$) $\delta$ 118.2 (2C), 147.9 (2C), 138.5 (2C), 130.0 (2C), 122.0 (2C), 120.7 (2C), 118.8 (2C), 112.8 (2C), 112.0 (2C).

### 4.1.2 Preparation of IN2

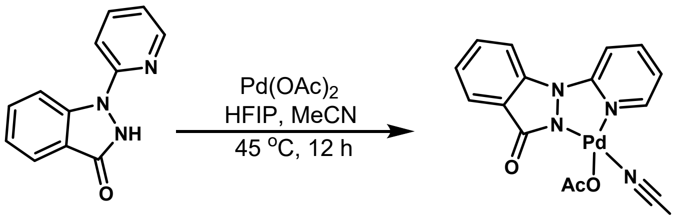


L2 IN2

A reaction tube (10 mL) with magnetic stir bar was charged with 1-(Pyridin-2-yl)-1,2-dihydro-3*H*-indazol-3-one **L2** (78 mg, 0.25 mmol), $Pd(OAc)_2$ (47 mg, 0.20 mmol), MeCN (0.5 mL) and HFIP (5 mL). The reaction was allowed to stir at room temperature for 12 h. Upon completion, the reaction mixture was evaporated to remove the solvent and the solid obtained was washed with ether to remove excess **L2**. Analytically pure intermediate **IN2** was obtained yellow solids (61 mg) in 73% yield by recrystallization using DCM/EA at room temperature. **IN2** was then used to perform NMR.

$^1$H NMR (400 MHz, $CDCl_3$:$CD_3OD$ = 1:6): $\delta$ 9.44 (d, $J$ = 5.2 Hz, 1H), 7.97−7.93 (m, 1H), 7.84−7.81 (m, 2H), 7.74 (d, $J$ = 8.4 Hz, 1H), 7.61 (t, $J$ = 8.4 Hz, 1H), 7.32 (t, $J$ = 7.2 Hz, 1H), 6.94 (t, $J$ = 6.8 Hz, 1H), 2.01 (s, 3H), 1.97 (s, 3H); $^{13}$C NMR (100 MHz, $CDCl_3$:$CD_3OD$ = 1:6): $\delta$ 194.0, 176.1, 155.9, 143.2, 142.3, 133.7, 133.0, 125.5, 124.6, 118.7, 114.4, 114.3, 109.8, 21.9, 2.0.

### 4.1.3 The reaction of IN1 to IN2

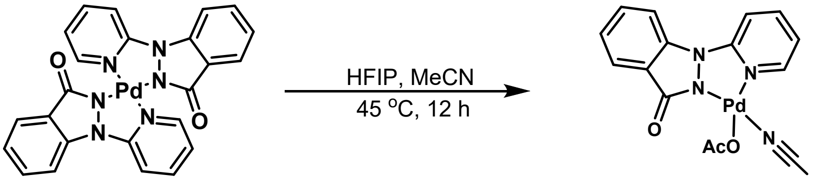


IN1 IN2

A reaction tube (5 mL) with magnetic stir bar was charged with **IN1** (53 mg, 0.10 mmol), MeCN (0.5 mL) and HFIP (5 mL). The reaction was allowed to stir at 45 °C oil bath for 12 h. Upon completion, the reaction mixture was evaporated to remove the solvent and the solid obtained was washed with ether to remove excess complexes. Analytically pure intermediate **IN2** was obtained as yellow solids (37 mg) in 82% yield by recrystallization using DCM/EA at room temperature.

## 4.2 1a Leading to Products 2a Using IN1 or IN2

### 4.2.1 1a leading to product 2a using IN1

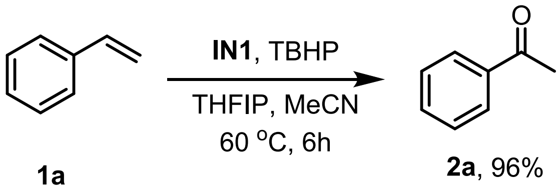


A reaction tube (10 mL) with magnetic stir bar was charged with olefins **1a** (1.0 mmol), **IN1** (10.0 mg, 0.02 mmol), TBHP (5 mmol/L in decane, 600 μL, 3.0 mmol), MeCN (0.5 mL)and HFIP (5.5 mL). The reaction was allowed to stir at 60°C oil bath for 6 h. After cooling to room temperature, the reaction mixture was evaporated to remove the solvent and directly loaded onto silica gel for flash column chromatography (PET/EtOAc) to afford the desired products **2a** (115 mg) in 96% yield.

### 4.2.1 1a leading to product 2a using IN2

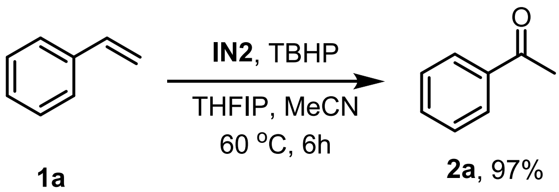


A reaction tube (10 mL) with magnetic stir bar was charged with olefins **1a** (1.0 mmol), **IN2** (8.0 mg, 0.02 mmol), TBHP (5 mmol/L in decane, 600 μL, 3.0 mmol), MeCN (0.5 mL)and HFIP (5.5 mL). The reaction was allowed to stir at 60°C oil bath for 6 h. After cooling to room temperature, the reaction mixture was evaporated to remove the solvent and directly loaded onto silica gel for flash column chromatography (PET/EtOAc) to afford the desired products **2a** (117 mg) in 97% yield.

## 4.3 1a leading to products 2a with IN1 and extra $Pd(OAc)_2$

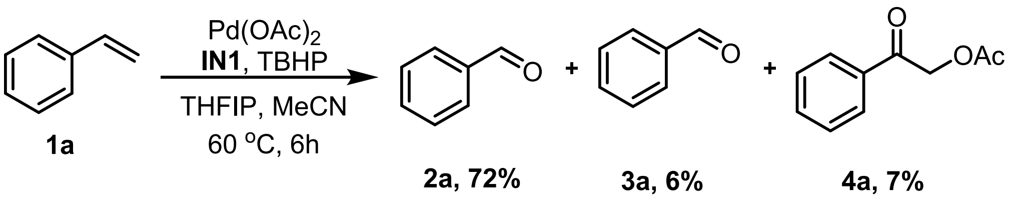


A reaction tube (20 mL) with magnetic stir bar was charged with olefins **1a** (2.0 mmol), **IN1** (21.0 mg, 0.04 mmol), $Pd(OAc)_2$ (23.4 mg, 0.10 mmol), TBHP (5 mmol/L in decane, 1.2 mL, 3.0 mmol), MeCN (1.0 mL)and HFIP (12 mL). The reaction was allowed to stir at 60°C oil bath for 6 h. After cooling to room temperature, the reaction mixture was evaporated to remove the solvent and directly loaded onto silica gel for flash column chromatography (PET/EtOAc) to afford the desired products **2a** (172 mg) in 72% yield. Intriguingly, the incorporation of $Pd(OAc)_2$ leads to the concurrent production of both **3a** and **4a**, with the yields of 6% and 7%, respectively.

## 4.4 1a leading to products 2a with IN2 and extra $Pd(OAc)_2$

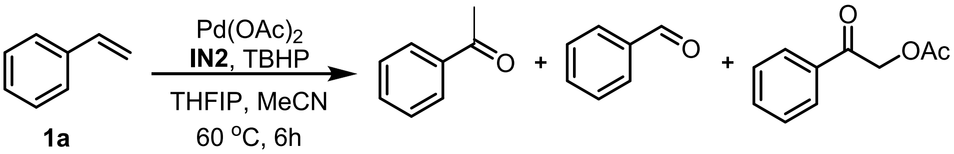


**1a** **2a, 74%** **3a, 4%** **4a, 10%**

A reaction tube (20 mL) with magnetic stir bar was charged with olefins **1a** (2.0 mmol), **IN2** (16.0 mg, 0.04 mmol), $Pd(OAc)_2$ (23.4 mg, 0.10 mmol), TBHP (5 mmol/L in decane, 1.2 mL, 3.0 mmol), MeCN (1.0 mL)and HFIP (12 mL). The reaction was allowed to stir at 60°C oil bath for 6 h. After cooling to room temperature, the reaction mixture was evaporated to remove the solvent and directly loaded onto silica gel for flash column chromatography (PET/EtOAc) to afford the desired products **2a** (178 mg) in 74% yield. Intriguingly, the incorporation of $Pd(OAc)_2$ leads to the concurrent production of both **3a** and **4a**, with the yields of 4% and 10%, respectively.

# 5. References


(1) Teo, Y.; Yong, F.; Sim, S. Ligand-free Cu2O-catalyzed cross coupling of nitrogen heterocycles with iodopyridines. *Tetrahedron* **2013**, *69*, 7279–7284.

# 8. NMR Spectra

## L1

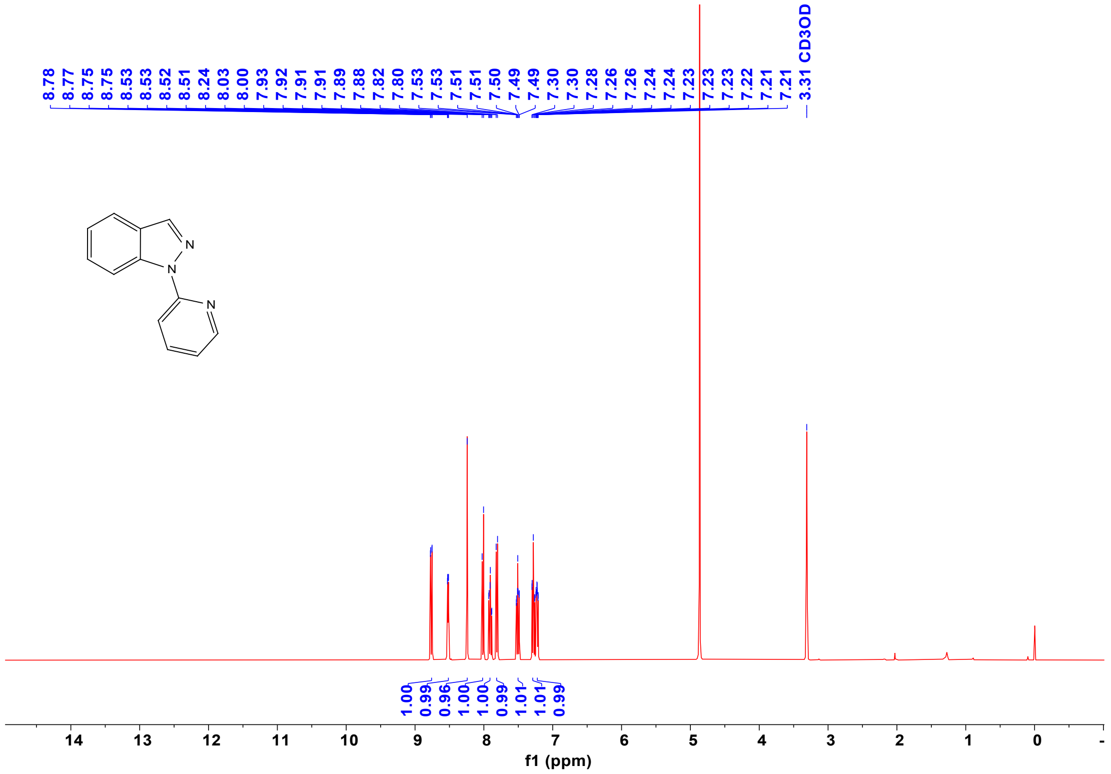


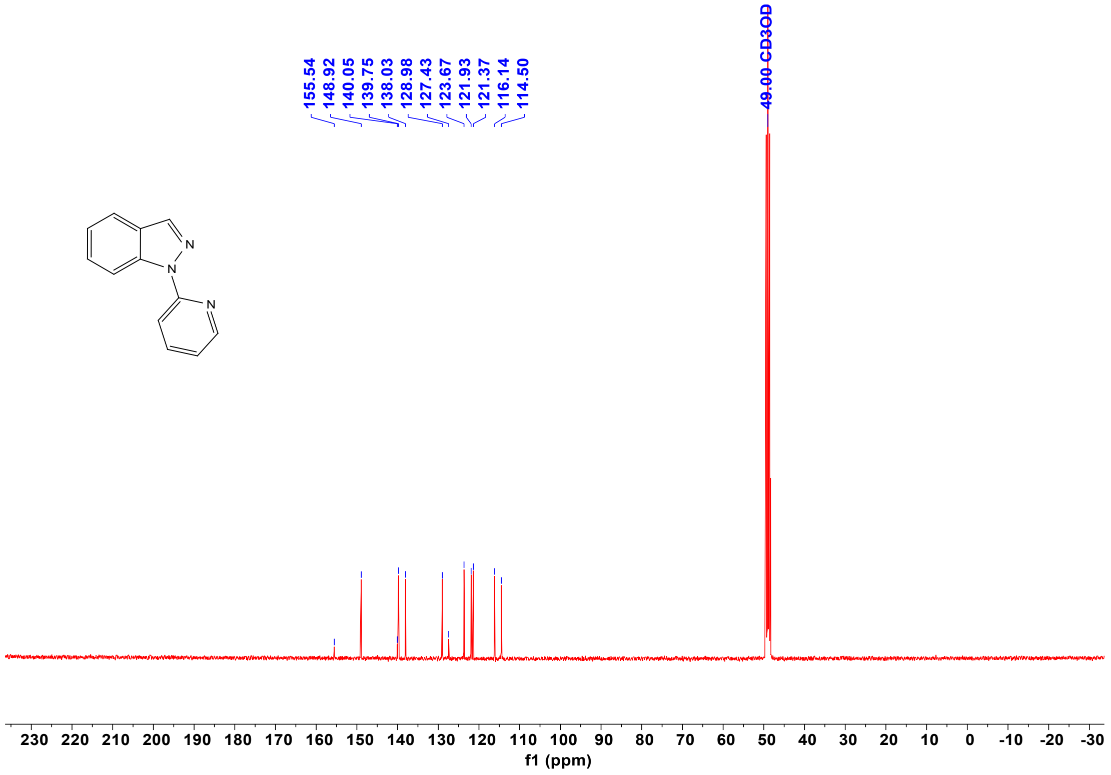

# L2

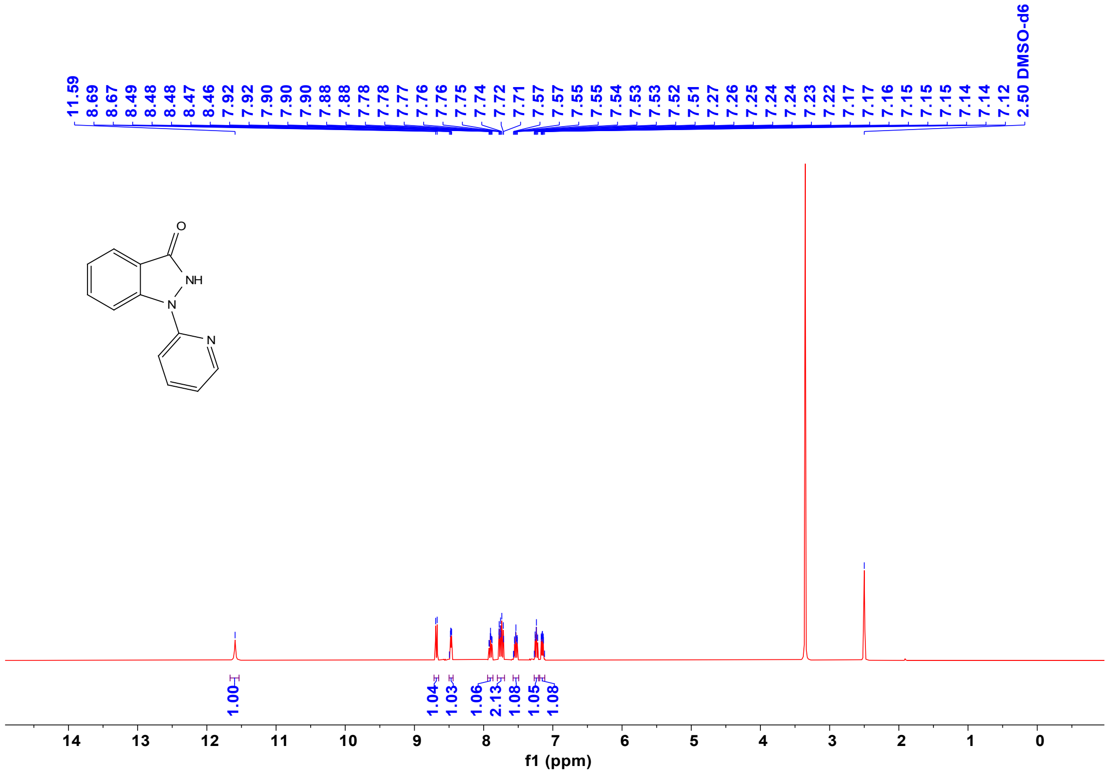
11.59
8.69
8.67
8.49
8.48
8.48
8.47
8.46
7.92
7.92
7.90
7.90
7.90
7.88
7.88
7.78
7.78
7.77
7.76
7.76
7.75
7.74
7.72
7.71
7.57
7.57
7.55
7.55
7.54
7.53
7.53
7.52
7.51
7.27
7.26
7.25
7.24
7.24
7.23
7.22
7.17
7.17
7.16
7.15
7.15
7.15
7.14
7.14
7.12
2.50 DMSO-d6
O
NH
N
N
1.00
1.04
1.03
1.06
2.13
1.08
1.05
1.08
14 13 12 11 10 9 8 7 6 5 4 3 2 1 0
f1 (ppm)

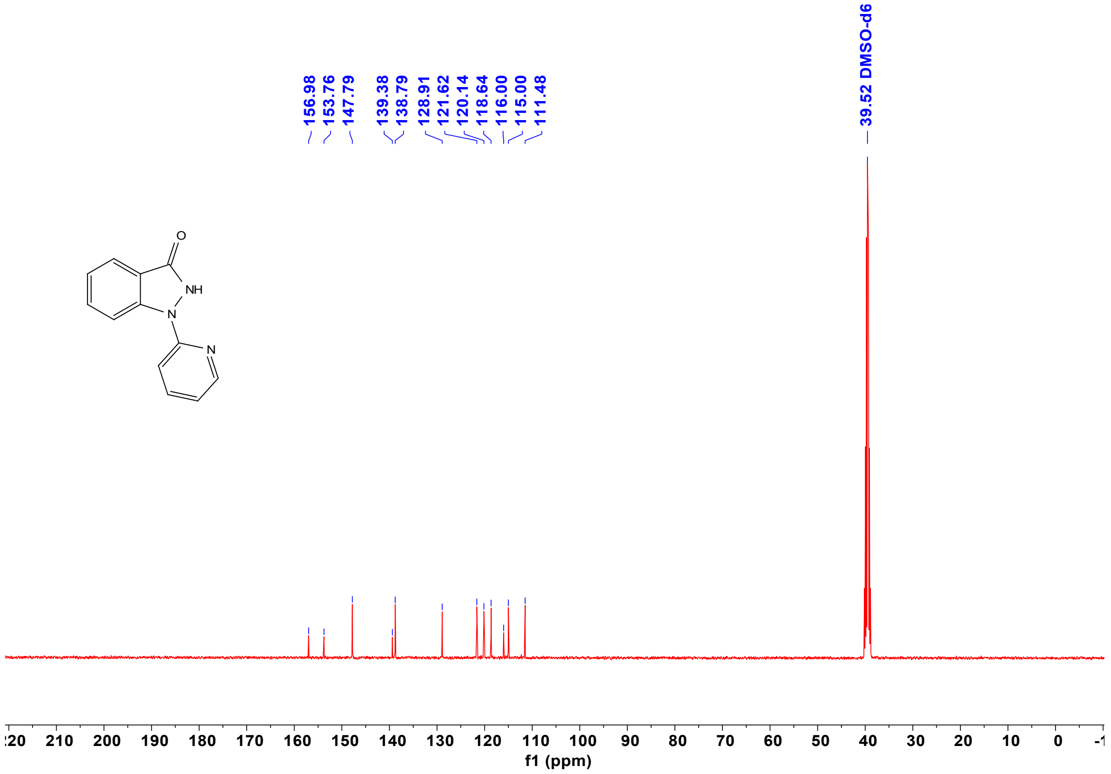
156.98
153.76
147.79
139.38
138.79
128.91
121.62
120.14
118.64
116.00
115.00
111.48
39.52 DMSO-d6
O
NH
N
N
220 210 200 190 180 170 160 150 140 130 120 110 100 90 80 70 60 50 40 30 20 10 0 -10
f1 (ppm)

# L3

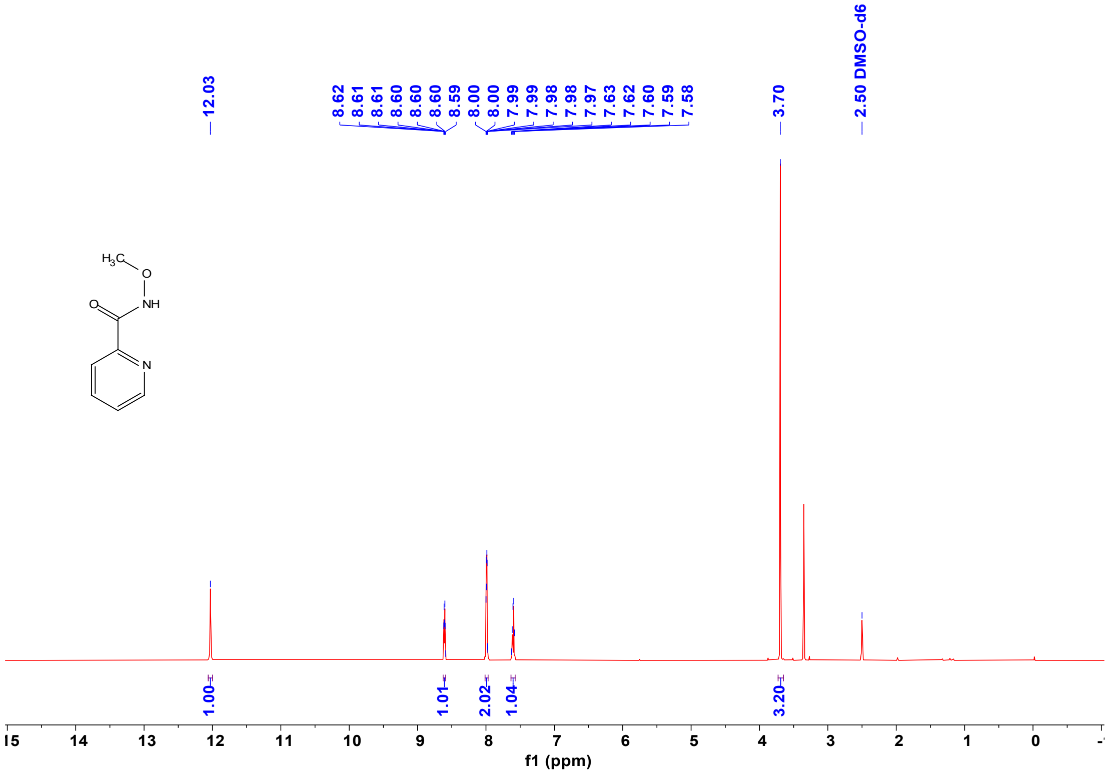

12.03
8.62
8.61
8.61
8.60
8.60
8.60
8.59
8.00
8.00
7.99
7.99
7.98
7.98
7.97
7.63
7.62
7.60
7.59
7.58
3.70
2.50 DMSO-d6
1.00
1.01
2.02
1.04
3.20
f1 (ppm)


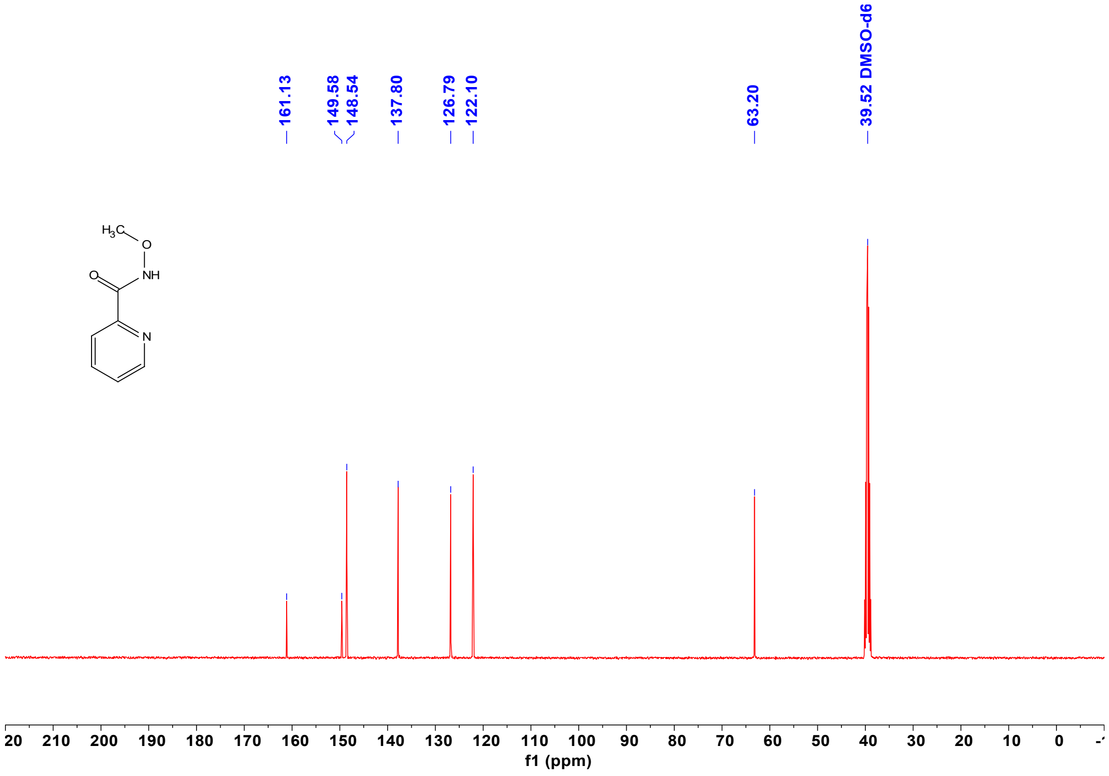

161.13
149.58
148.54
137.80
126.79
122.10
63.20
39.52 DMSO-d6
f1 (ppm)

# L4

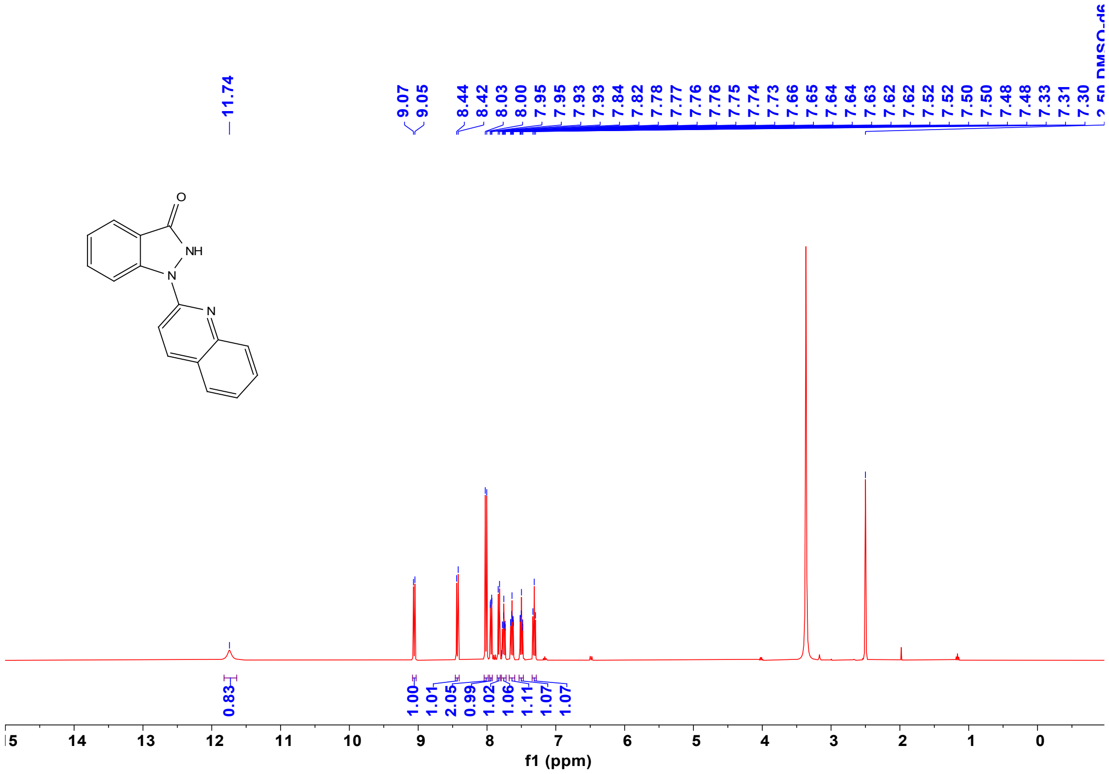
11.74
9.07
9.05
8.44
8.42
8.03
8.00
7.95
7.95
7.93
7.93
7.84
7.82
7.78
7.77
7.76
7.76
7.75
7.74
7.73
7.66
7.65
7.64
7.64
7.63
7.62
7.62
7.52
7.52
7.50
7.48
7.48
7.33
7.31
7.30
2.50 DMSO-d6
O
NH
N
N
0.83
1.00
1.01
2.05
0.99
1.02
1.06
1.11
1.07
1.07
15 14 13 12 11 10 9 8 7 6 5 4 3 2 1 0
f1 (ppm)

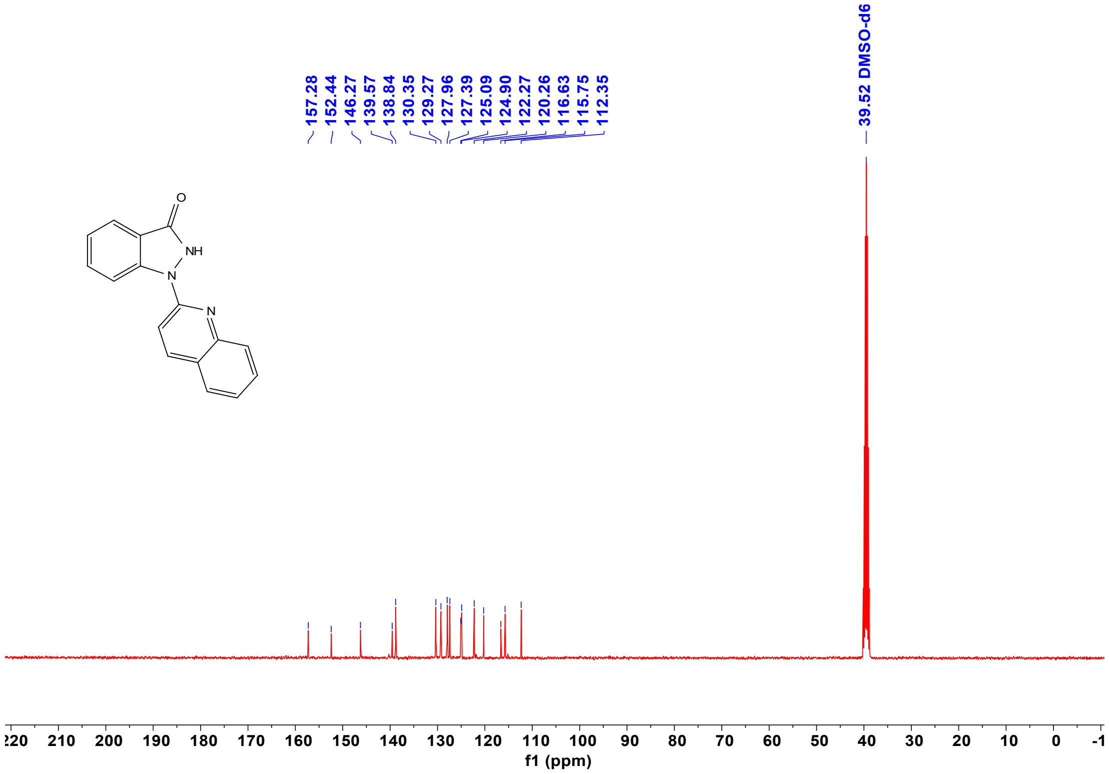
157.28
152.44
146.27
139.57
138.84
130.35
129.27
127.96
127.39
125.09
124.90
122.27
120.26
116.63
115.75
112.35
39.52 DMSO-d6
O
NH
N
N
220 210 200 190 180 170 160 150 140 130 120 110 100 90 80 70 60 50 40 30 20 10 0 -1
f1 (ppm)

# L5

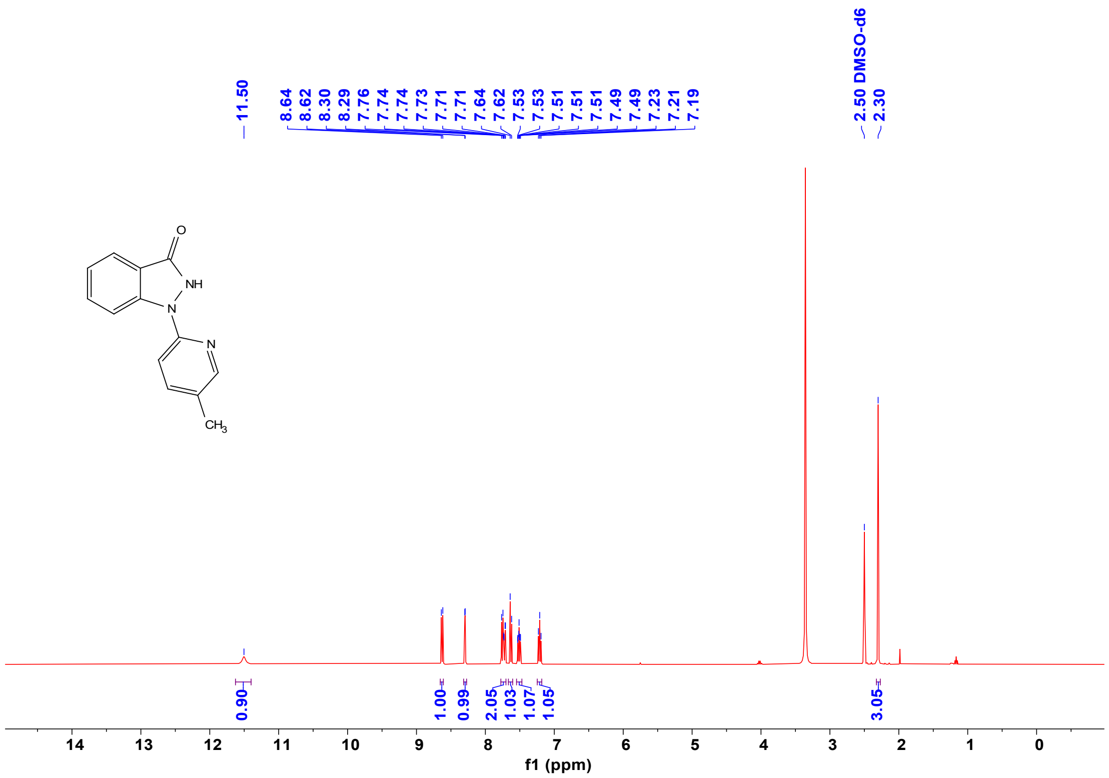

11.50
8.64
8.62
8.30
8.29
7.76
7.74
7.74
7.73
7.71
7.71
7.64
7.62
7.53
7.53
7.51
7.51
7.51
7.49
7.49
7.23
7.21
7.19
2.50 DMSO-d6
2.30
O
NH
N
N
CH3
0.90
1.00
0.99
2.05
1.03
1.07
1.05
3.05
14
13
12
11
10
9
8
7
6
5
4
3
2
1
0
f1 (ppm)


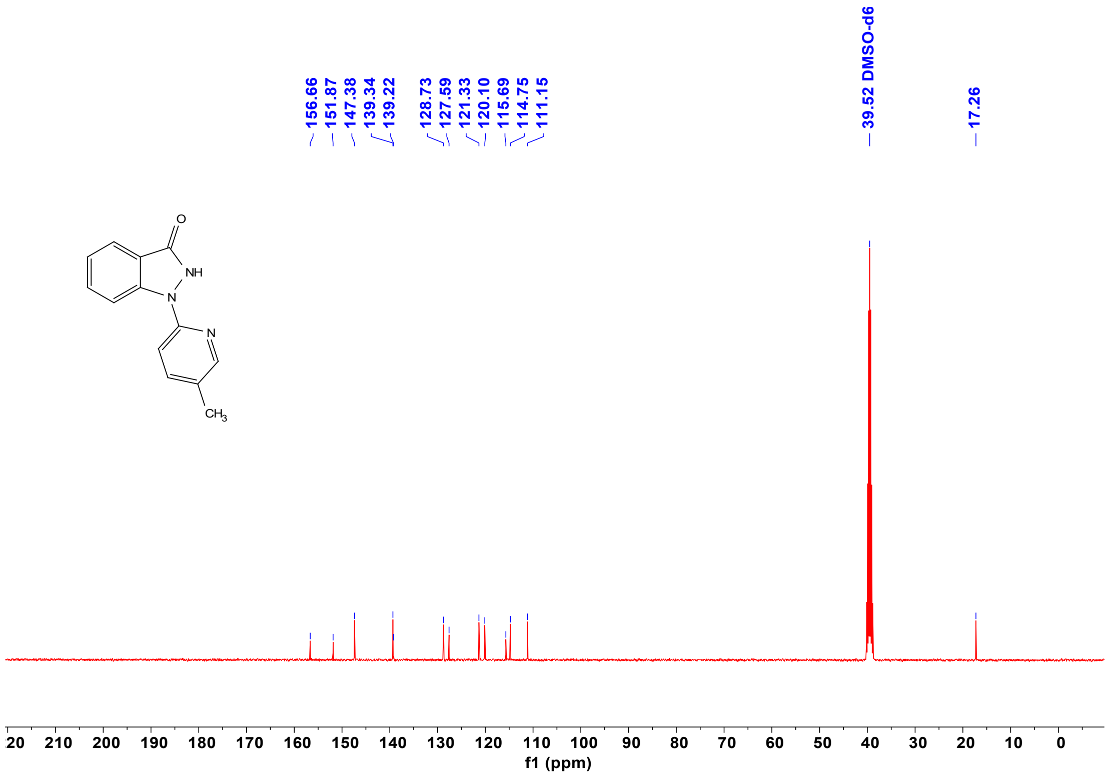

156.66
151.87
147.38
139.34
139.22
128.73
127.59
121.33
120.10
115.69
114.75
111.15
39.52 DMSO-d6
17.26
O
NH
N
N
CH3
20
210
200
190
180
170
160
150
140
130
120
110
100
90
80
70
60
50
40
30
20
10
0
f1 (ppm)

# L6

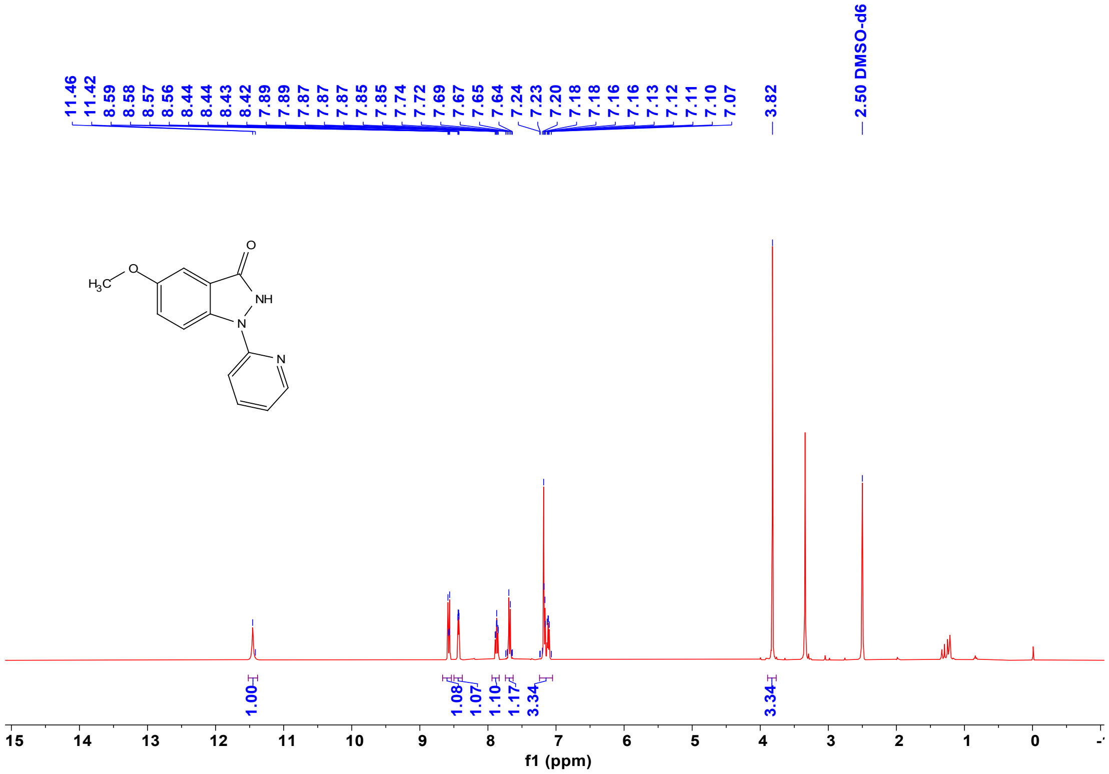

11.46
11.42
8.59
8.58
8.57
8.56
8.44
8.44
8.43
8.42
7.89
7.89
7.87
7.87
7.87
7.85
7.85
7.74
7.72
7.69
7.67
7.65
7.64
7.24
7.23
7.20
7.18
7.16
7.16
7.13
7.12
7.11
7.10
7.07
3.82
2.50 DMSO-d6
1.00
1.08
1.07
1.10
1.17
3.34
3.34
f1 (ppm)


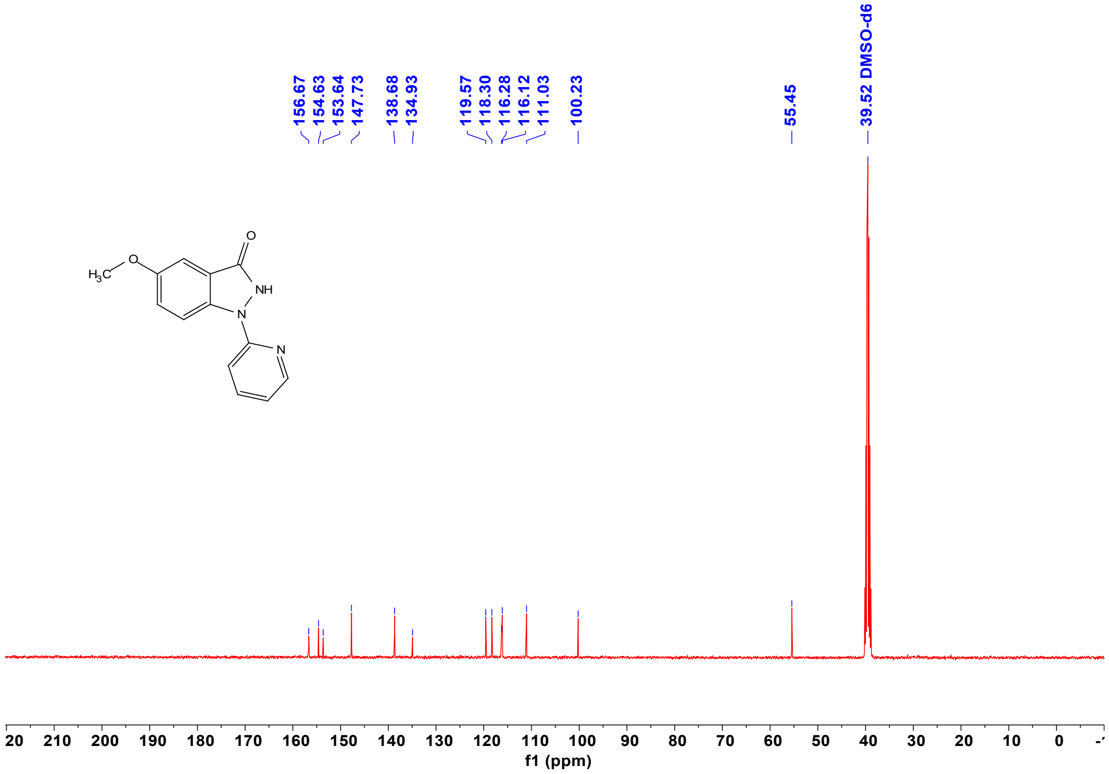

156.67
154.63
153.64
147.73
138.68
134.93
119.57
118.30
116.28
116.12
111.03
100.23
55.45
39.52 DMSO-d6
f1 (ppm)

**L7**

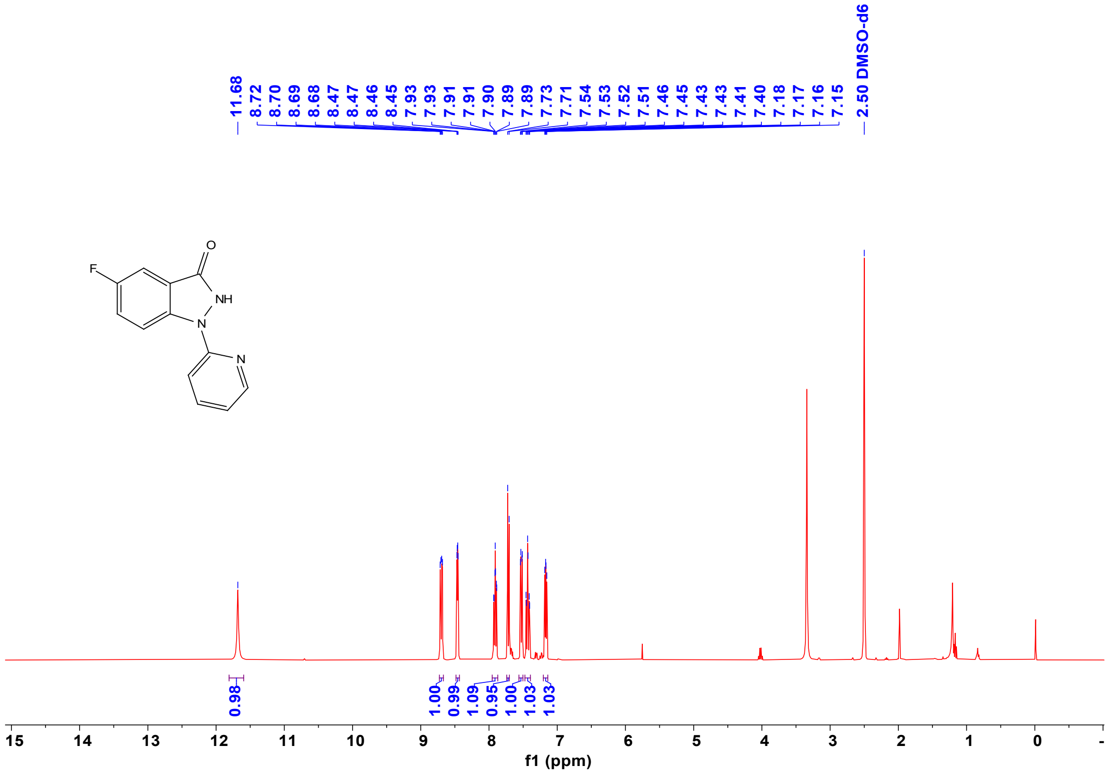
11.68
8.72
8.70
8.69
8.68
8.47
8.47
8.46
8.45
7.93
7.91
7.91
7.90
7.89
7.89
7.73
7.71
7.54
7.53
7.52
7.51
7.46
7.45
7.43
7.43
7.41
7.40
7.18
7.17
7.16
7.15
2.50 DMSO-d6
F
O
NH
N
N
0.98
1.00
0.99
1.09
0.95
1.00
1.03
1.03
f1 (ppm)

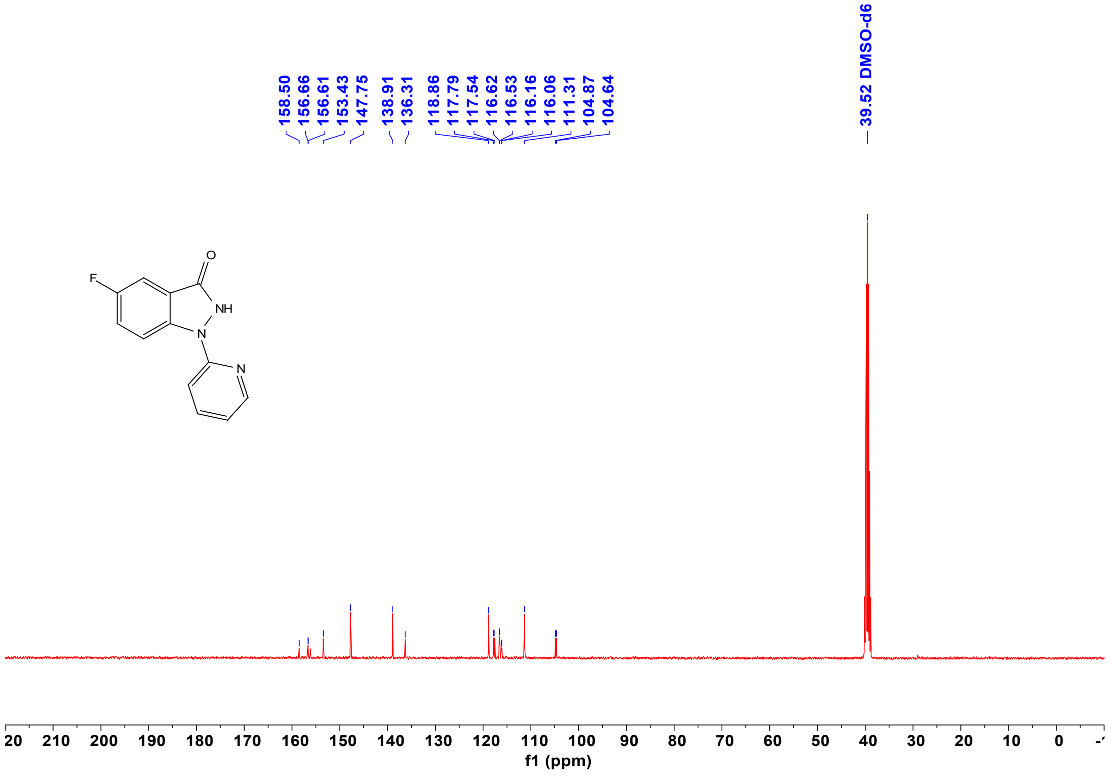
158.50
156.66
156.61
153.43
147.75
138.91
136.31
118.86
117.79
117.54
116.62
116.53
116.16
116.06
111.31
104.87
104.64
39.52 DMSO-d6
F
O
NH
N
N
f1 (ppm)

# L8

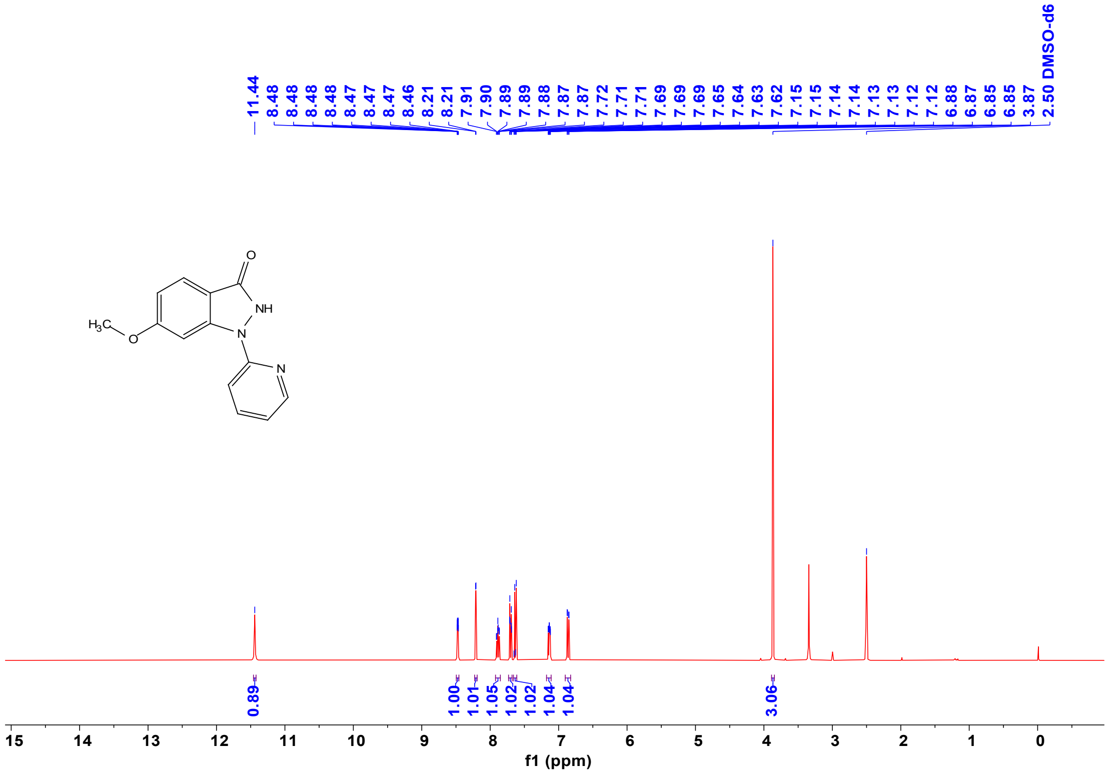
11.44
8.48
8.48
8.48
8.47
8.47
8.47
8.46
8.21
8.21
7.91
7.90
7.89
7.89
7.88
7.87
7.87
7.72
7.71
7.71
7.69
7.69
7.69
7.65
7.64
7.63
7.62
7.15
7.15
7.14
7.14
7.13
7.13
7.12
7.12
6.88
6.87
6.85
6.85
3.87
2.50 DMSO-d6
O
NH
N
N
$H_3C$
O
0.89
1.00
1.01
1.05
1.02
1.02
1.04
1.04
3.06
15
14
13
12
11
10
9
8
7
6
5
4
3
2
1
0
f1 (ppm)

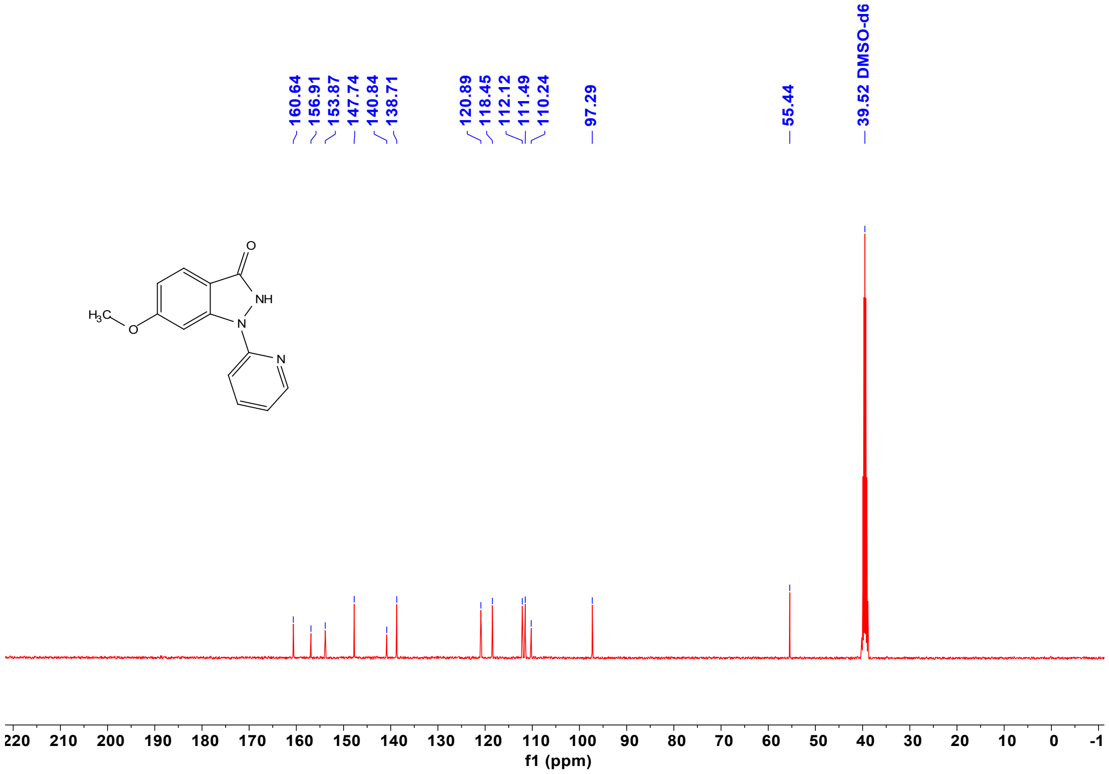
160.64
156.91
153.87
147.74
140.84
138.71
120.89
118.45
112.12
111.49
110.24
97.29
55.44
39.52 DMSO-d6
O
NH
N
N
$H_3C$
O
220
210
200
190
180
170
160
150
140
130
120
110
100
90
80
70
60
50
40
30
20
10
0
-1
f1 (ppm)

# 2a

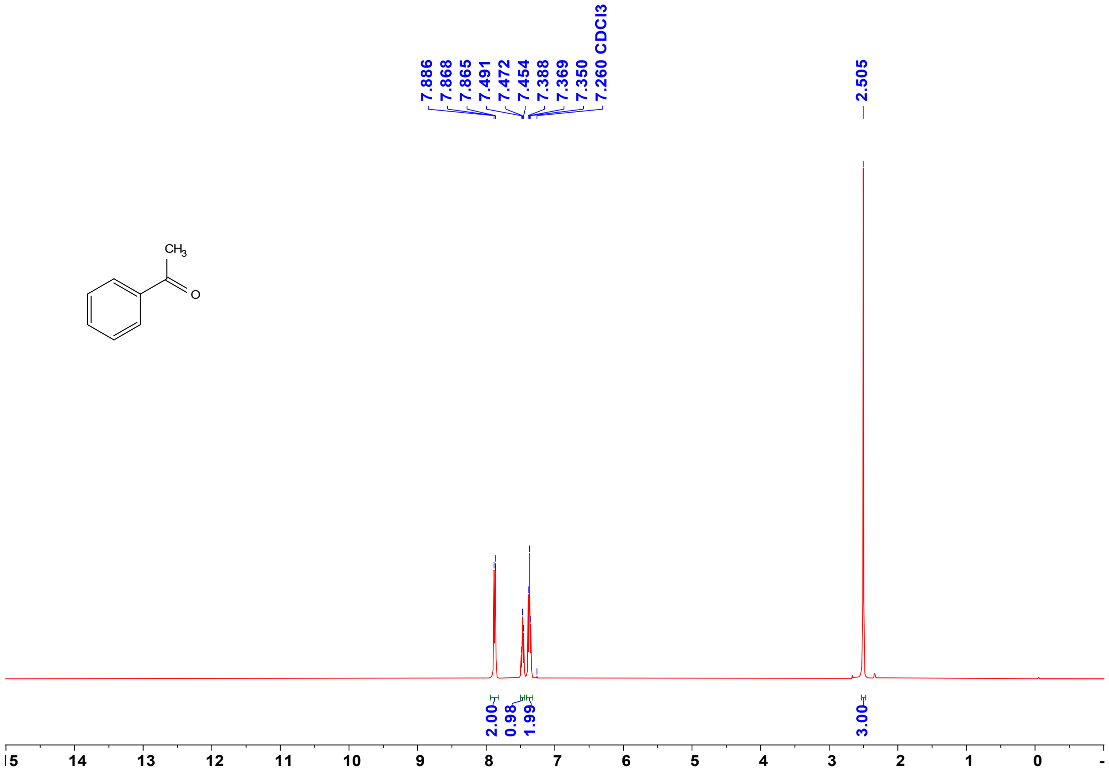
7.886
7.868
7.865
7.491
7.472
7.454
7.388
7.369
7.350
7.260 CDCl3
2.505
CH3
O
2.00
0.98
1.99
3.00
15 14 13 12 11 10 9 8 7 6 5 4 3 2 1 0 -

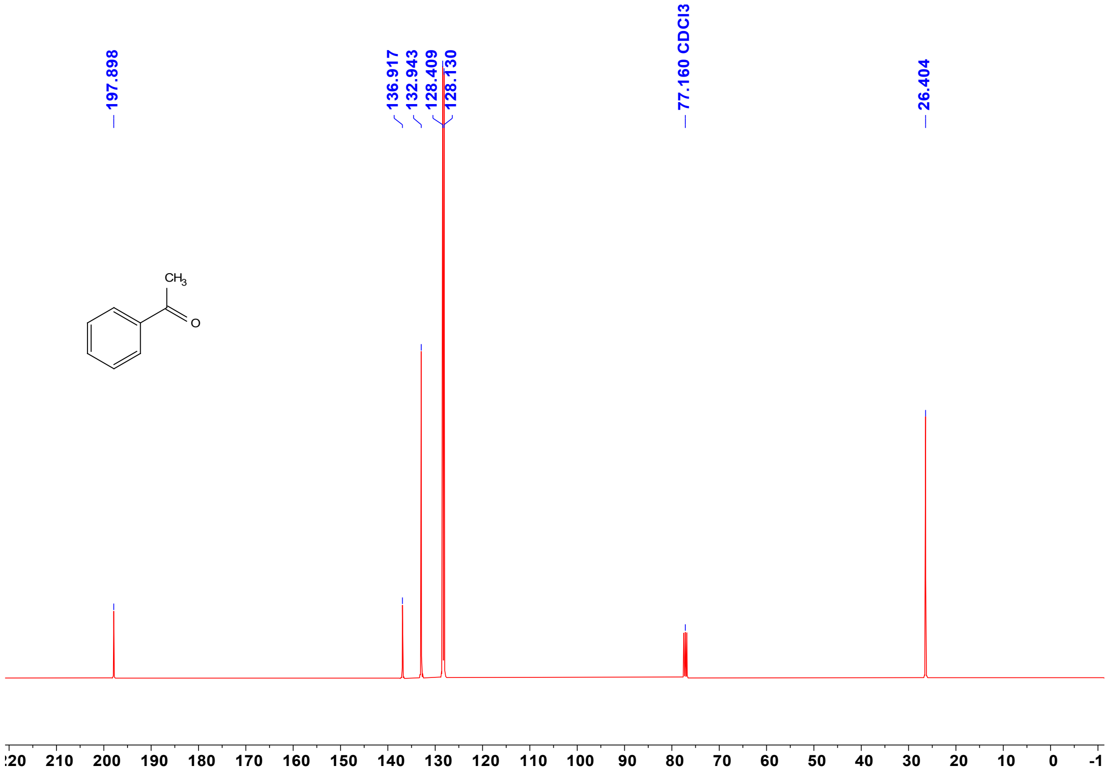
197.898
136.917
132.943
128.409
128.130
77.160 CDCl3
26.404
CH3
O
220 210 200 190 180 170 160 150 140 130 120 110 100 90 80 70 60 50 40 30 20 10 0 -1

# 2b

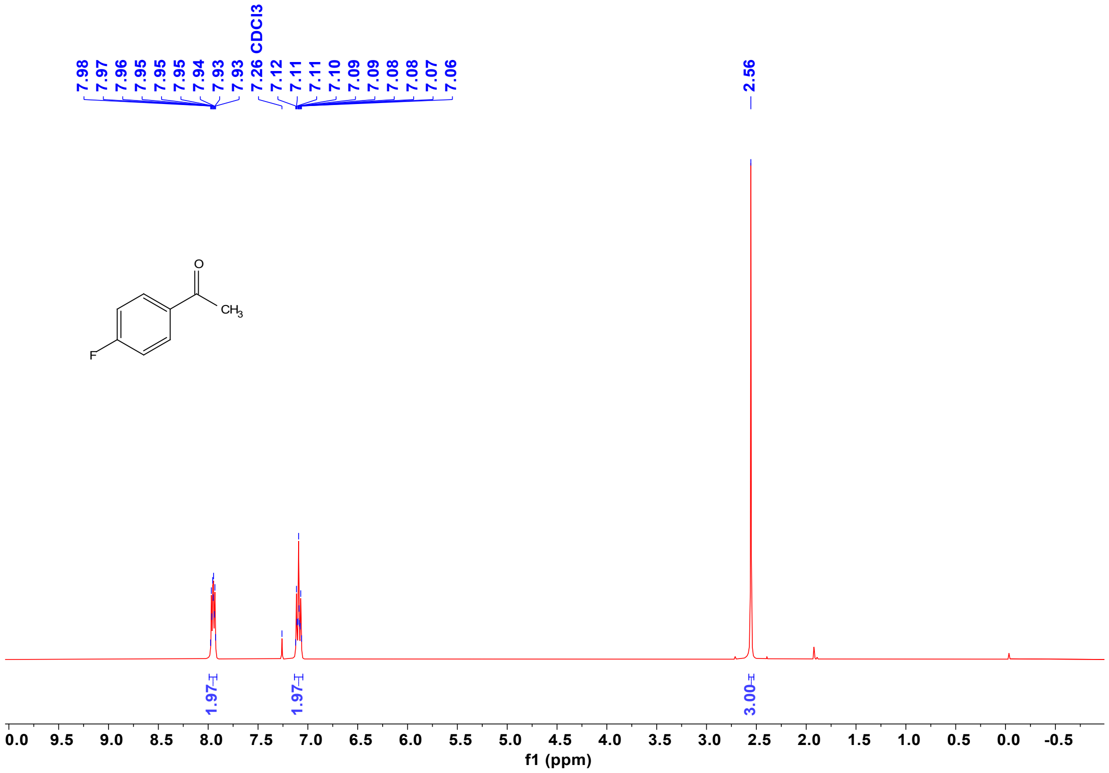

7.98
7.97
7.96
7.95
7.95
7.95
7.94
7.93
7.93
7.26 CDCl3
7.12
7.11
7.11
7.10
7.09
7.09
7.08
7.08
7.07
7.06
2.56
O
CH3
F
1.97
1.97
3.00
f1 (ppm)


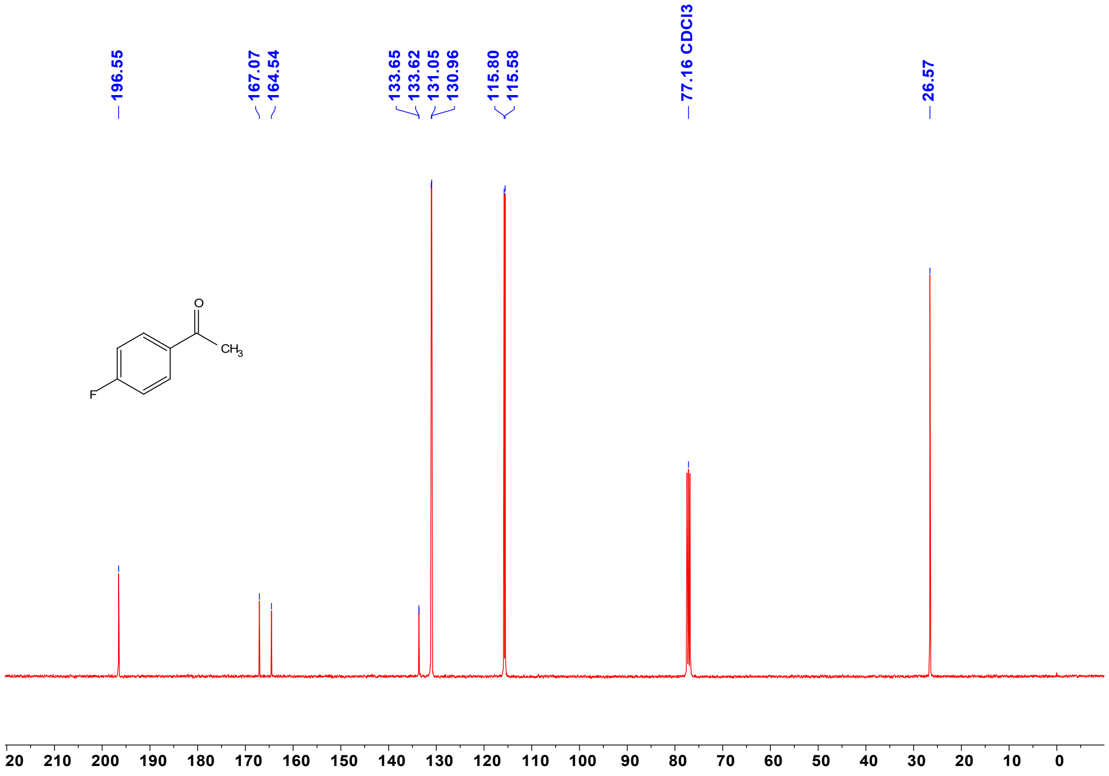

196.55
167.07
164.54
133.65
133.62
131.05
130.96
115.80
115.58
77.16 CDCl3
26.57
O
CH3
F

**2c**

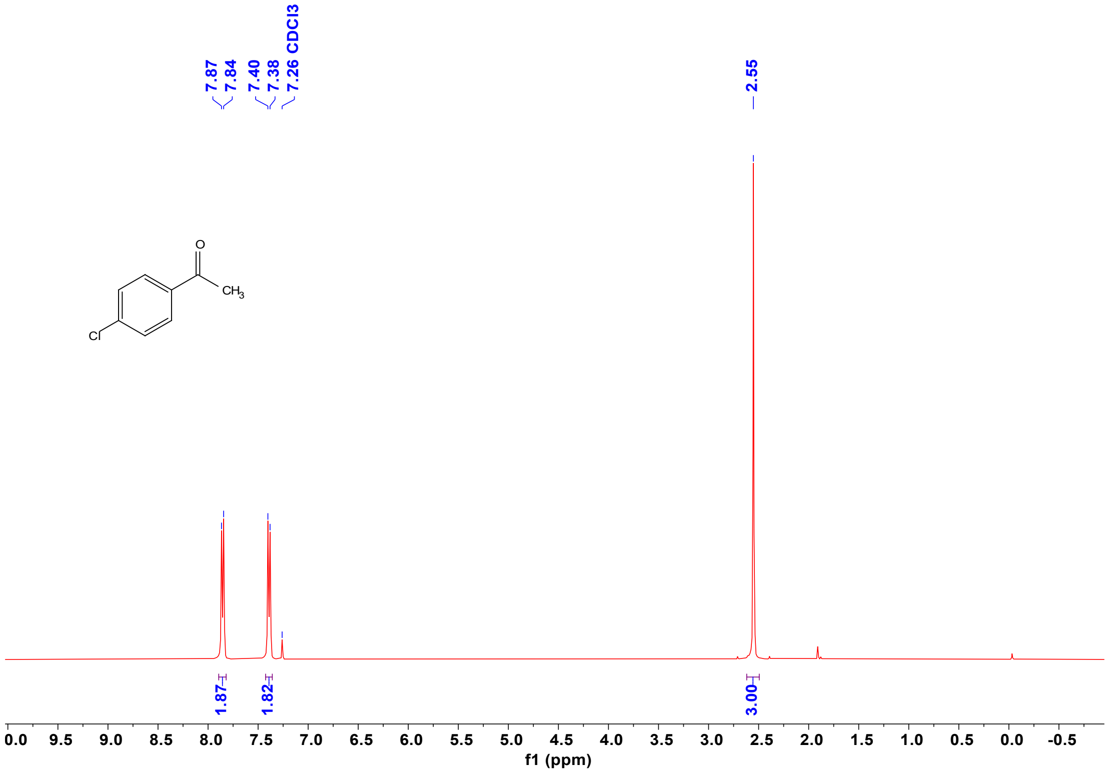

7.87
7.84
7.40
7.38
7.26 CDCl3
2.55
O
CH3
Cl
1.87
1.82
3.00
0.0 9.5 9.0 8.5 8.0 7.5 7.0 6.5 6.0 5.5 5.0 4.5 4.0 3.5 3.0 2.5 2.0 1.5 1.0 0.5 0.0 -0.5
f1 (ppm)


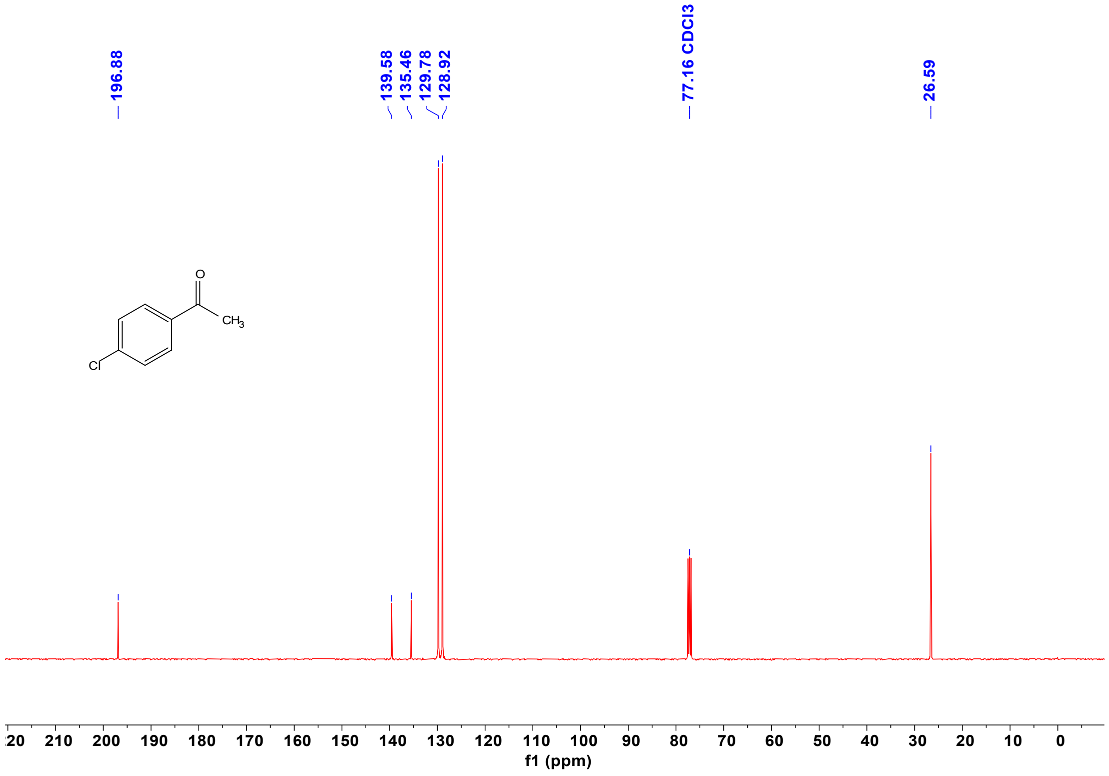

196.88
139.58
135.46
129.78
128.92
77.16 CDCl3
26.59
O
CH3
Cl
20 210 200 190 180 170 160 150 140 130 120 110 100 90 80 70 60 50 40 30 20 10 0
f1 (ppm)

**2d**

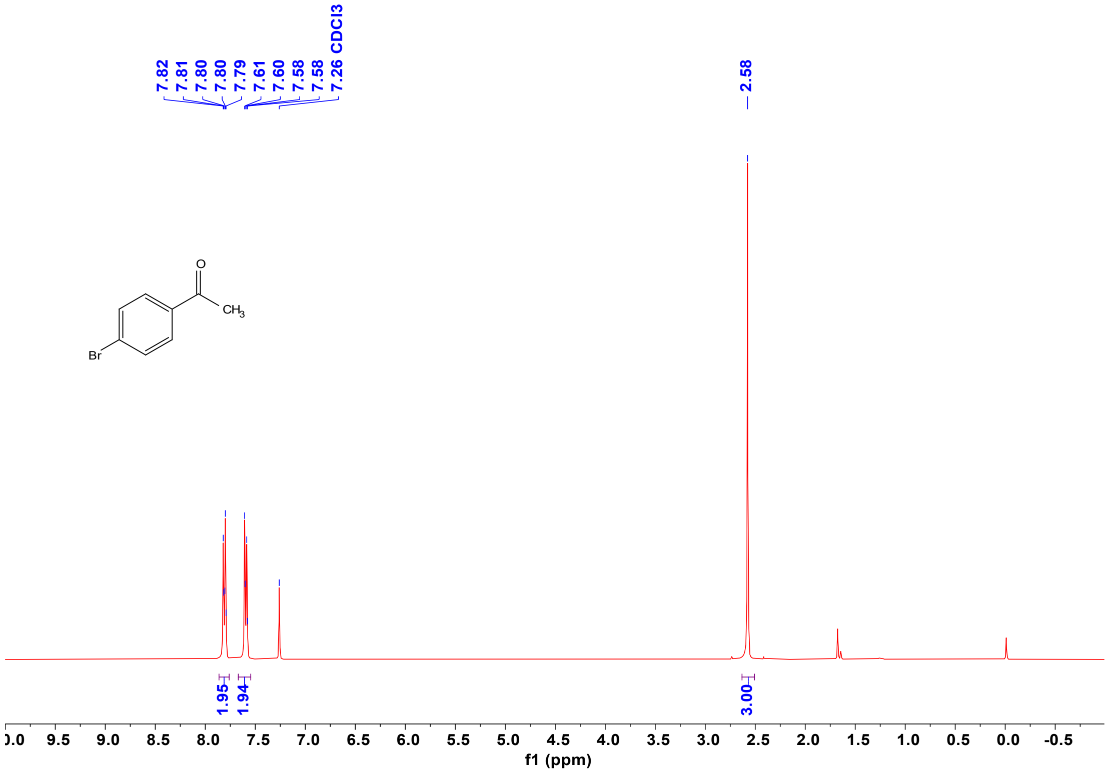
7.82
7.81
7.80
7.80
7.79
7.61
7.60
7.58
7.58
7.26 CDCl3
2.58
O
CH3
Br
1.95
1.94
3.00
f1 (ppm)

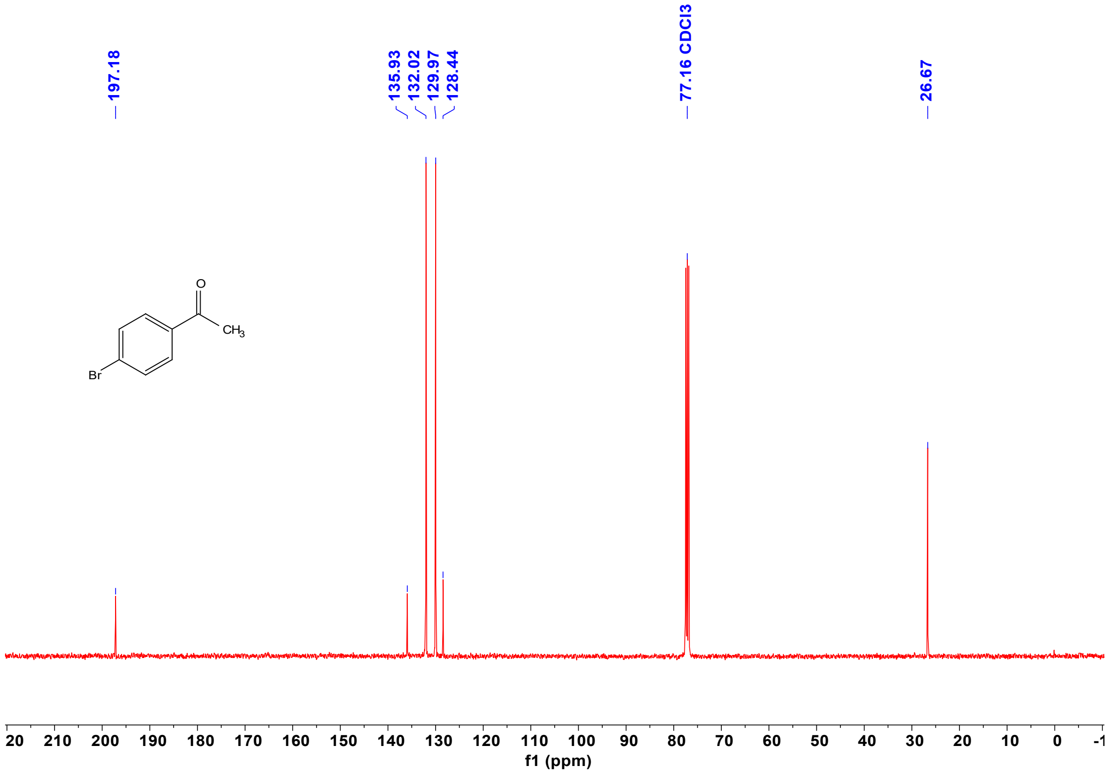
197.18
135.93
132.02
129.97
128.44
77.16 CDCl3
26.67
O
CH3
Br
f1 (ppm)

**2e**

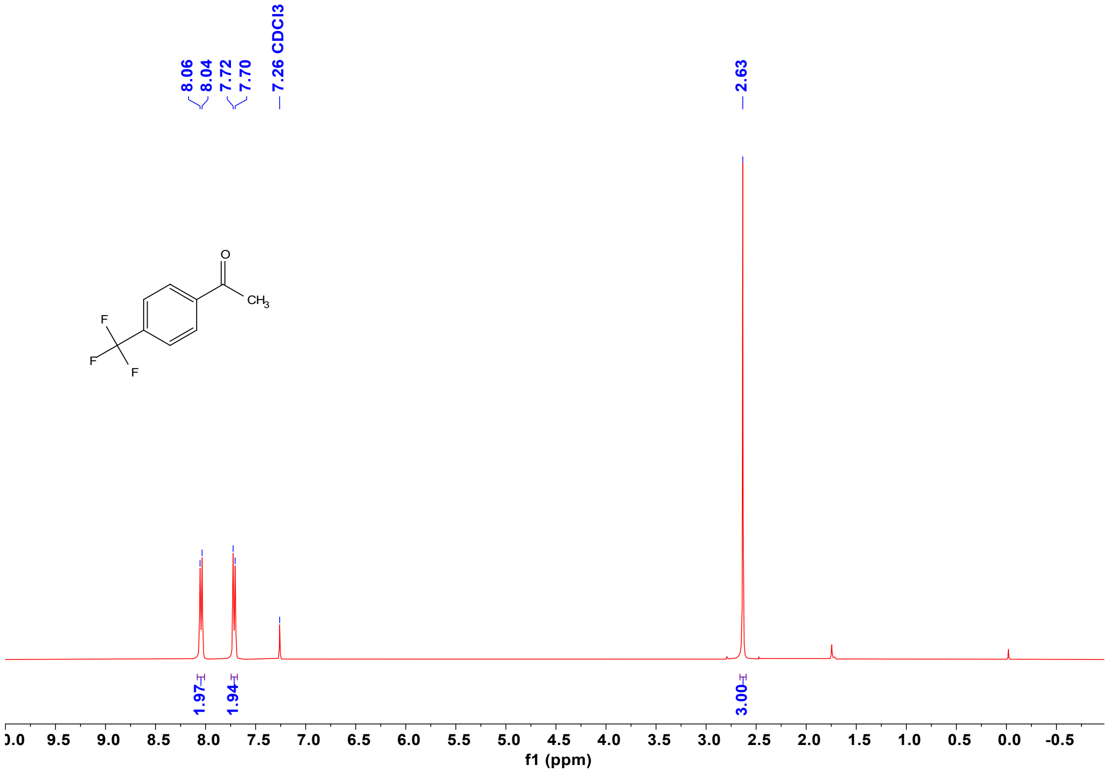

8.06
8.04
7.72
7.70
7.26 CDCl3
2.63
O
CH3
F
F
F
1.97
1.94
3.00
f1 (ppm)


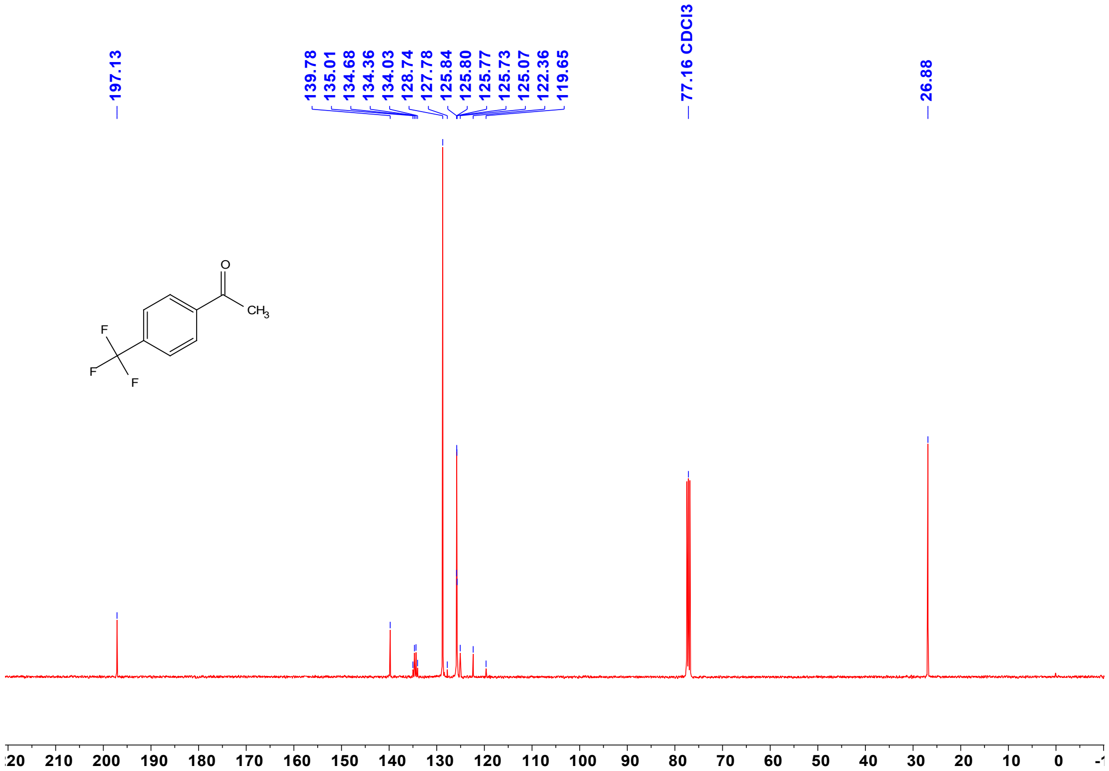

197.13
139.78
135.01
134.68
134.36
134.03
128.74
127.78
125.84
125.80
125.77
125.73
125.07
122.36
119.65
77.16 CDCl3
26.88
O
CH3
F
F
F

**2f**

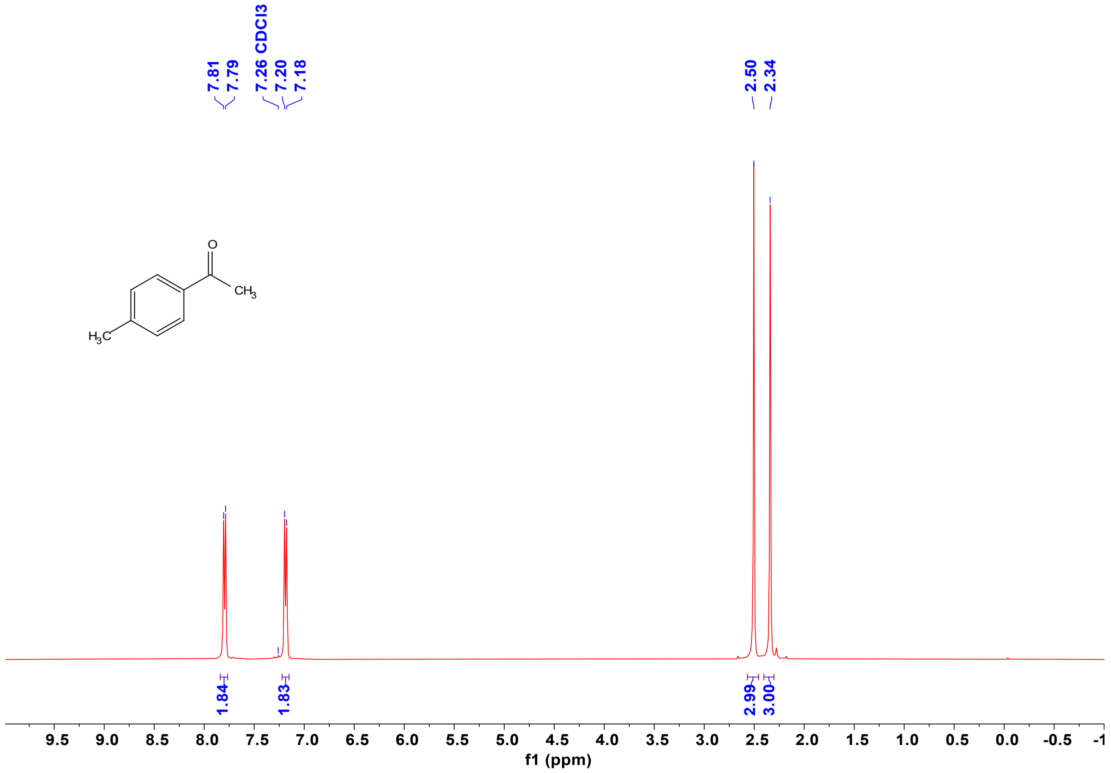
7.81
7.79
7.26 CDCl3
7.20
7.18
2.50
2.34
O
CH3
H3C
1.84
1.83
2.99
3.00
9.5
9.0
8.5
8.0
7.5
7.0
6.5
6.0
5.5
5.0
4.5
4.0
3.5
3.0
2.5
2.0
1.5
1.0
0.5
0.0
-0.5
-1
f1 (ppm)

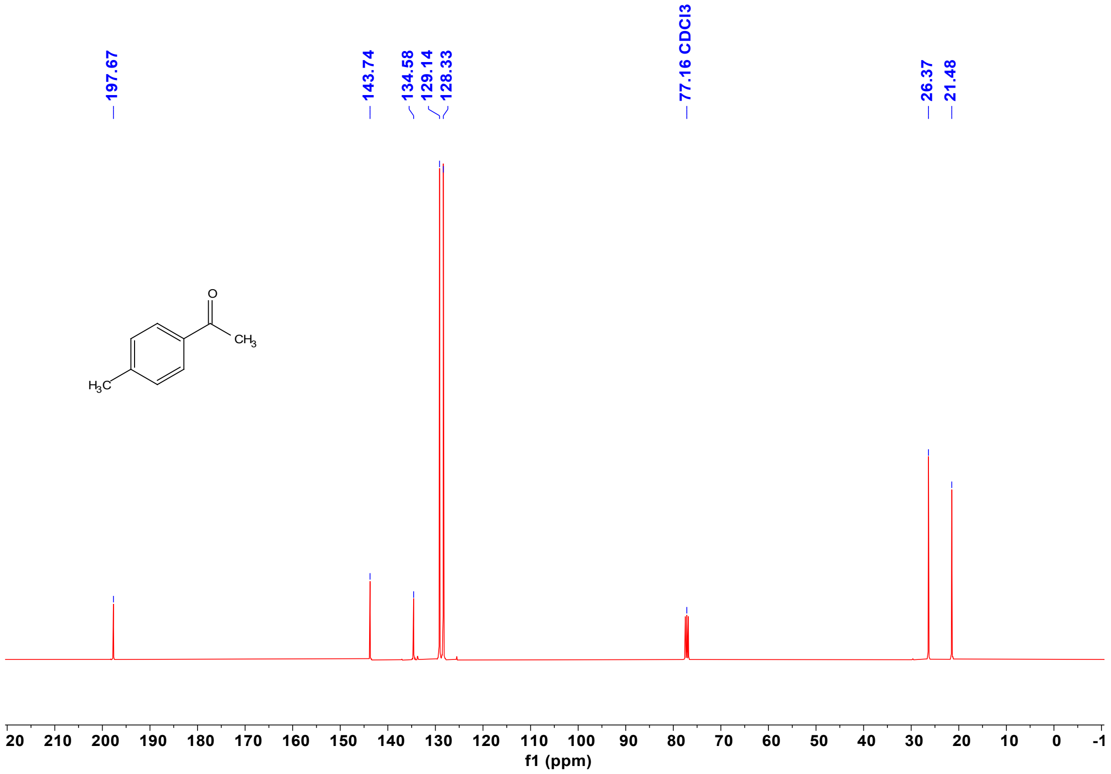
197.67
143.74
134.58
129.14
128.33
77.16 CDCl3
26.37
21.48
O
CH3
H3C
20
210
200
190
180
170
160
150
140
130
120
110
100
90
80
70
60
50
40
30
20
10
0
-1
f1 (ppm)

**2g**

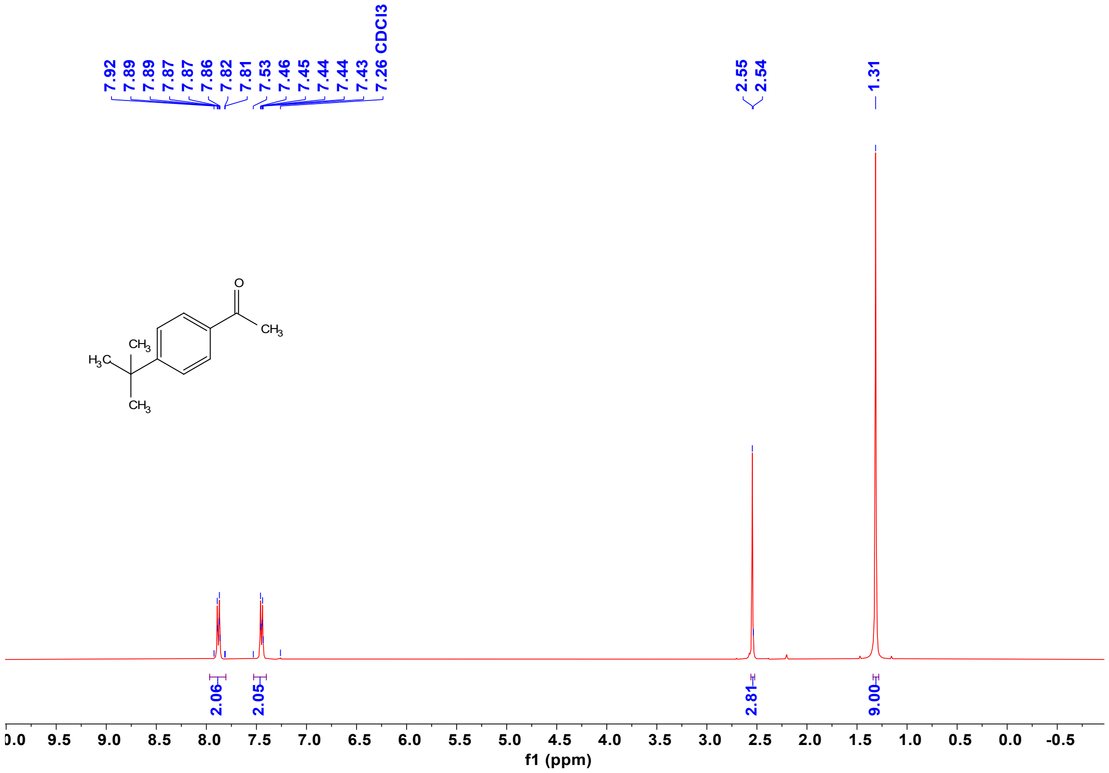

7.92
7.89
7.89
7.87
7.87
7.86
7.82
7.81
7.53
7.46
7.45
7.44
7.44
7.43
7.26 CDCl3
2.55
2.54
1.31
O
CH3
CH3
H3C
CH3
2.06
2.05
2.81
9.00
f1 (ppm)


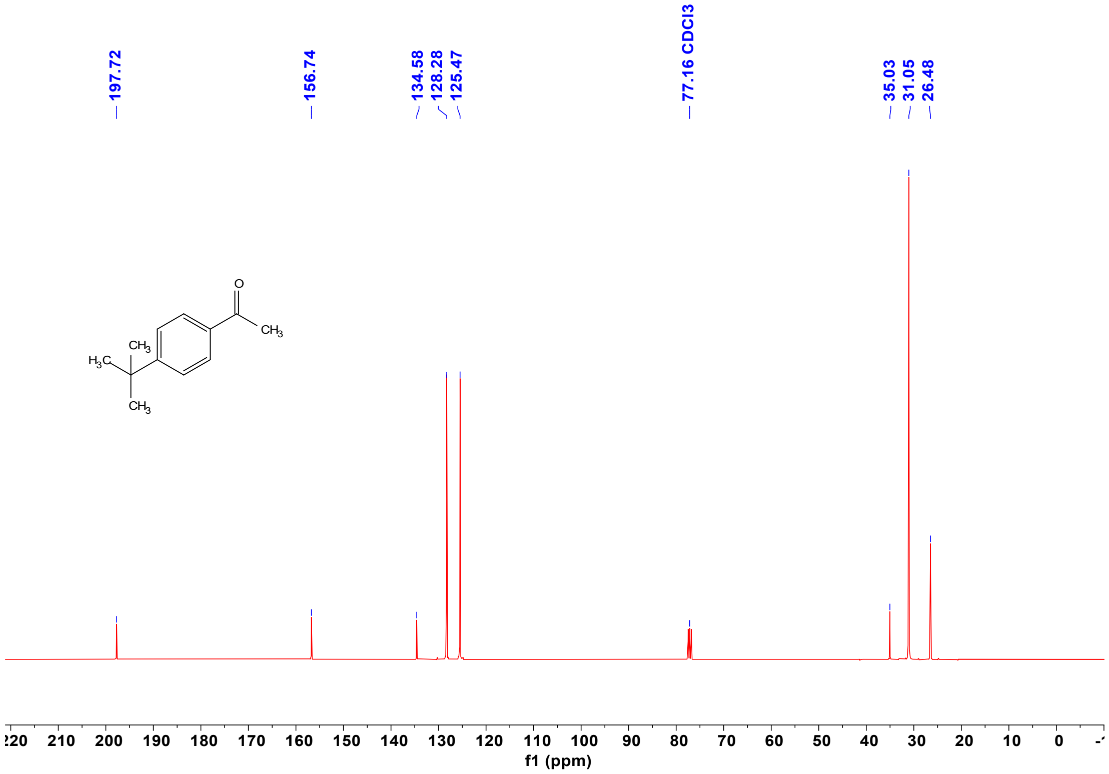

197.72
156.74
134.58
128.28
125.47
77.16 CDCl3
35.03
31.05
26.48
O
CH3
CH3
H3C
CH3
f1 (ppm)

## 2h

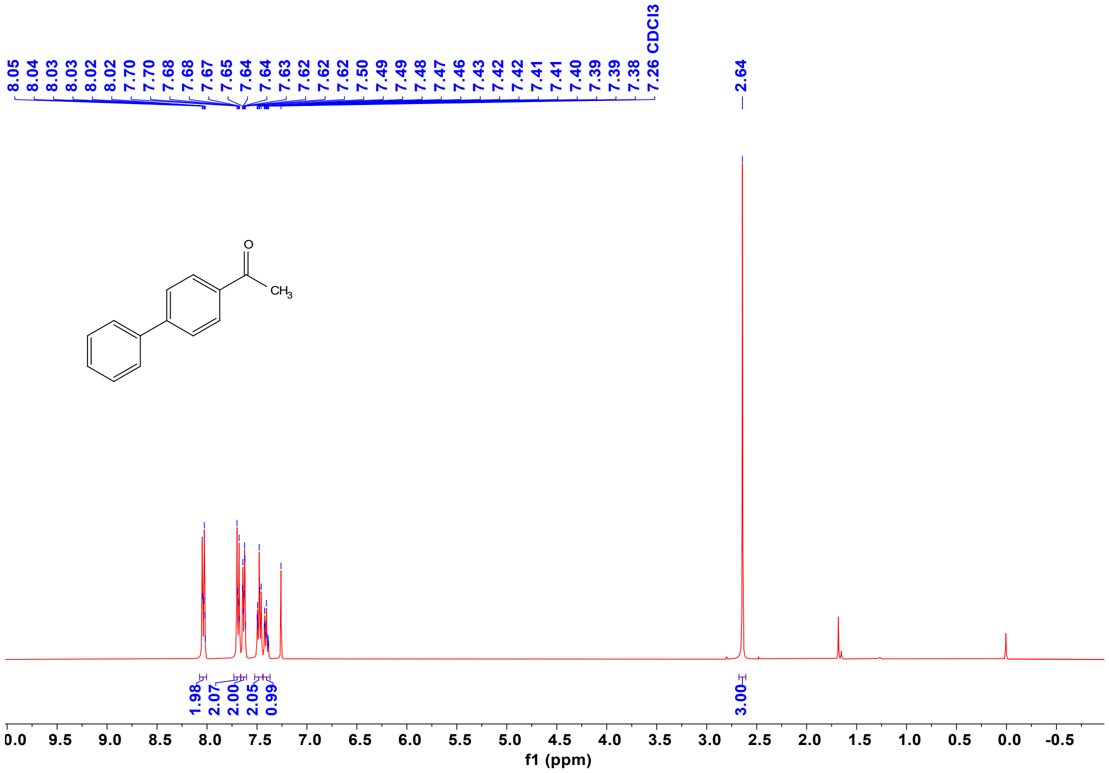

8.05
8.04
8.03
8.03
8.02
8.02
7.70
7.70
7.68
7.67
7.65
7.64
7.64
7.63
7.62
7.62
7.50
7.49
7.49
7.48
7.47
7.46
7.43
7.42
7.42
7.41
7.41
7.40
7.39
7.39
7.38
7.26 CDCl3
2.64
O
CH3
1.98
2.07
2.00
2.05
0.99
3.00
f1 (ppm)


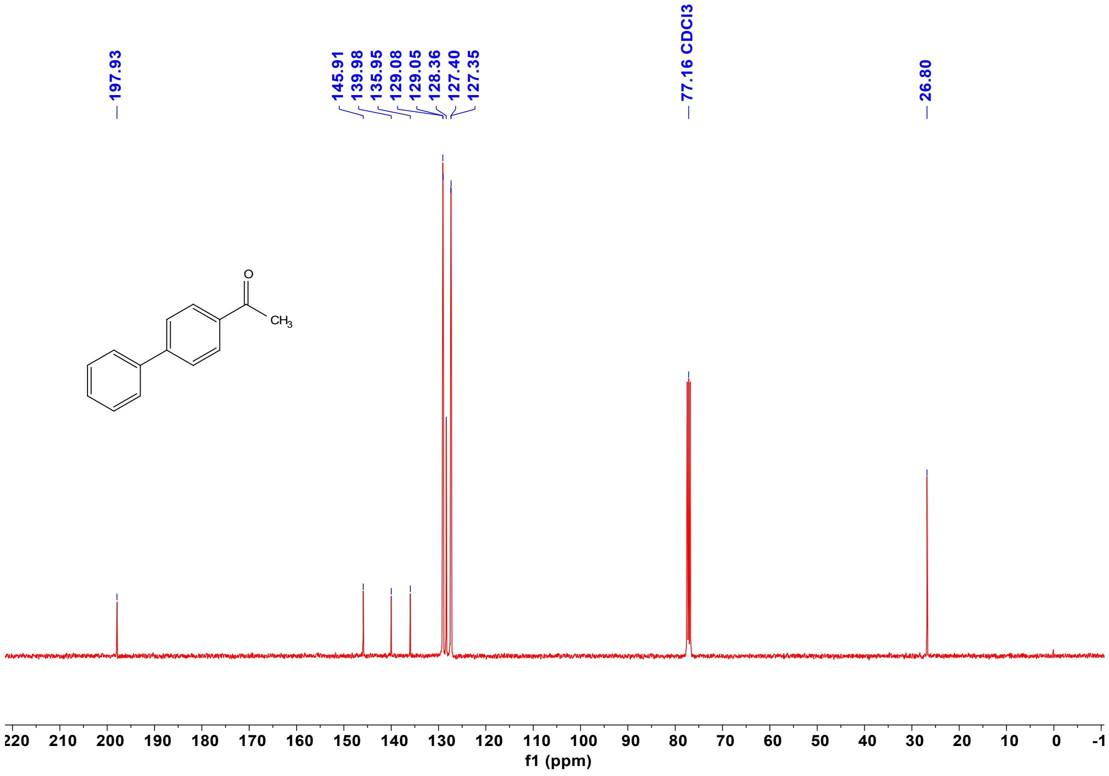

197.93
145.91
139.98
135.95
129.08
129.05
128.36
127.40
127.35
77.16 CDCl3
26.80
O
CH3
f1 (ppm)

**2i**

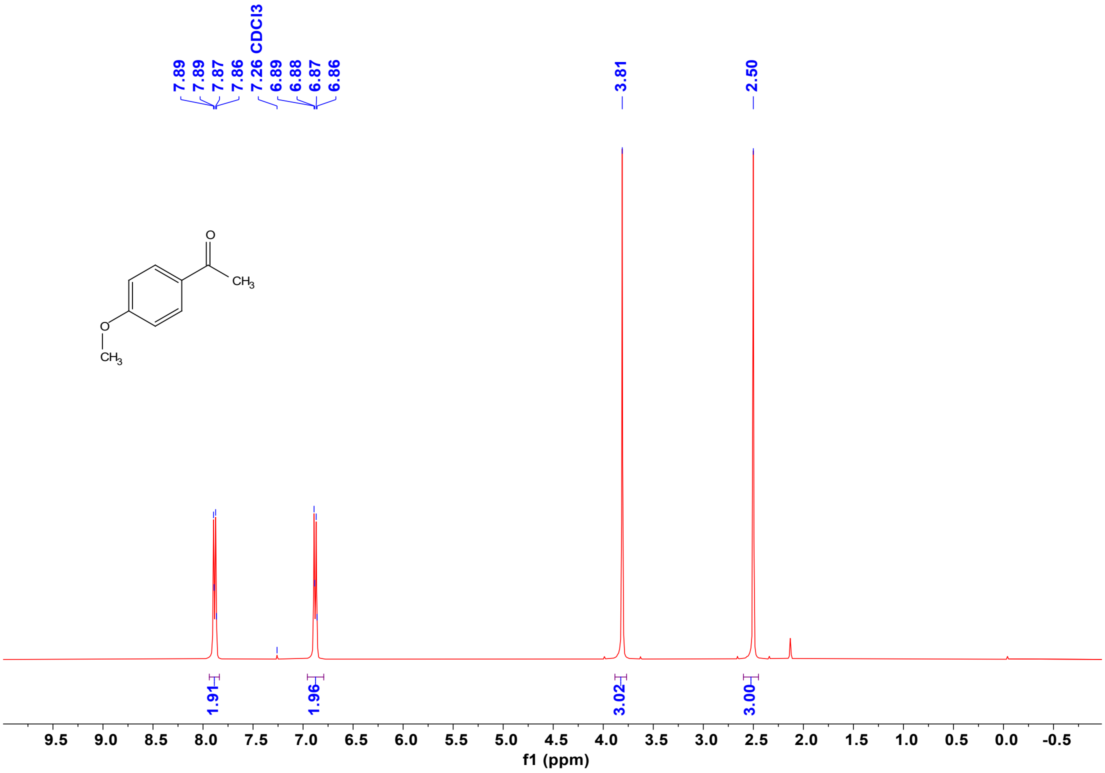
7.89
7.89
7.87
7.86
7.26 CDCl3
6.89
6.88
6.87
6.86
3.81
2.50
O
CH3
O
CH3
1.91
1.96
3.02
3.00
9.5 9.0 8.5 8.0 7.5 7.0 6.5 6.0 5.5 5.0 4.5 4.0 3.5 3.0 2.5 2.0 1.5 1.0 0.5 0.0 -0.5
f1 (ppm)

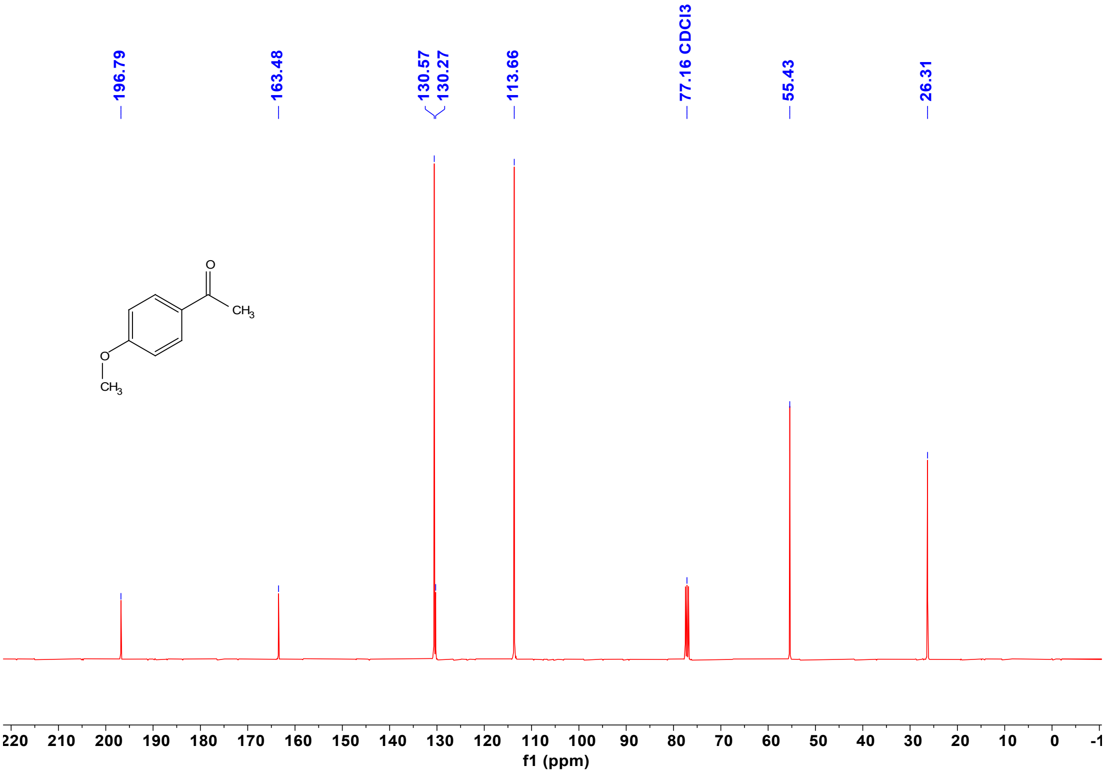
196.79
163.48
130.57
130.27
113.66
77.16 CDCl3
55.43
26.31
O
CH3
O
CH3
220 210 200 190 180 170 160 150 140 130 120 110 100 90 80 70 60 50 40 30 20 10 0 -1
f1 (ppm)

**2j**

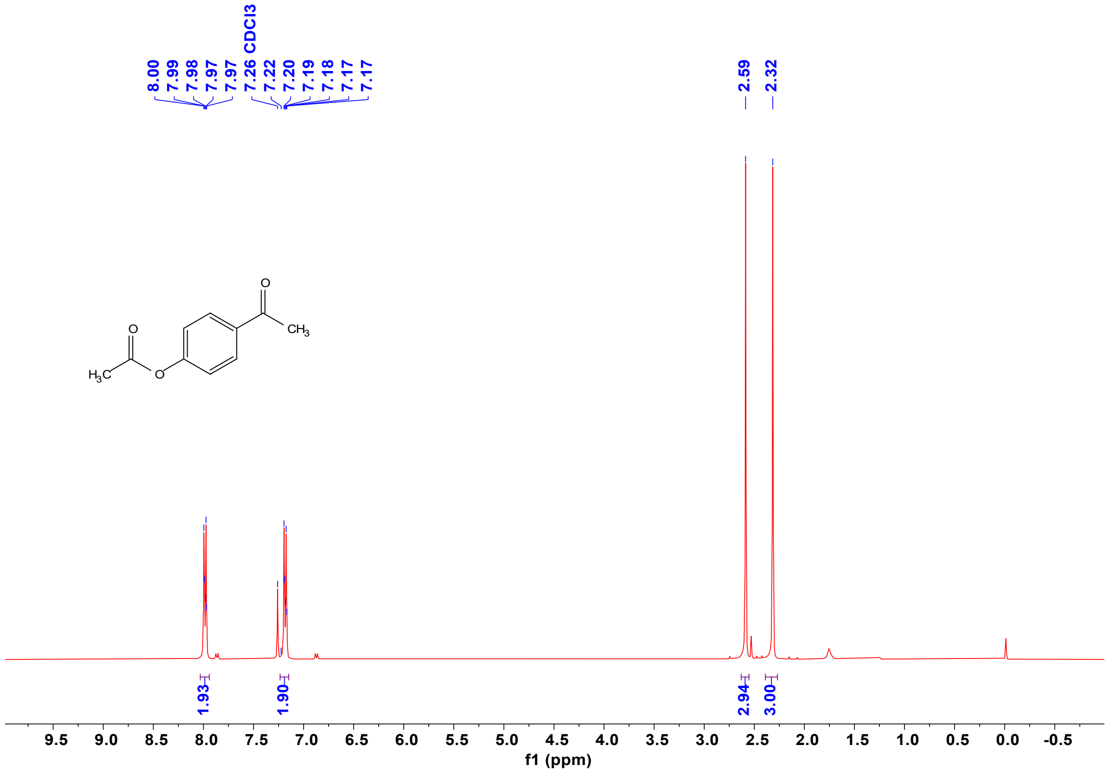
8.00
7.99
7.98
7.97
7.97
7.26 CDCl3
7.22
7.20
7.19
7.18
7.17
7.17
2.59
2.32
O
CH3
O
H3C
O
1.93
1.90
2.94
3.00
9.5 9.0 8.5 8.0 7.5 7.0 6.5 6.0 5.5 5.0 4.5 4.0 3.5 3.0 2.5 2.0 1.5 1.0 0.5 0.0 -0.5
f1 (ppm)

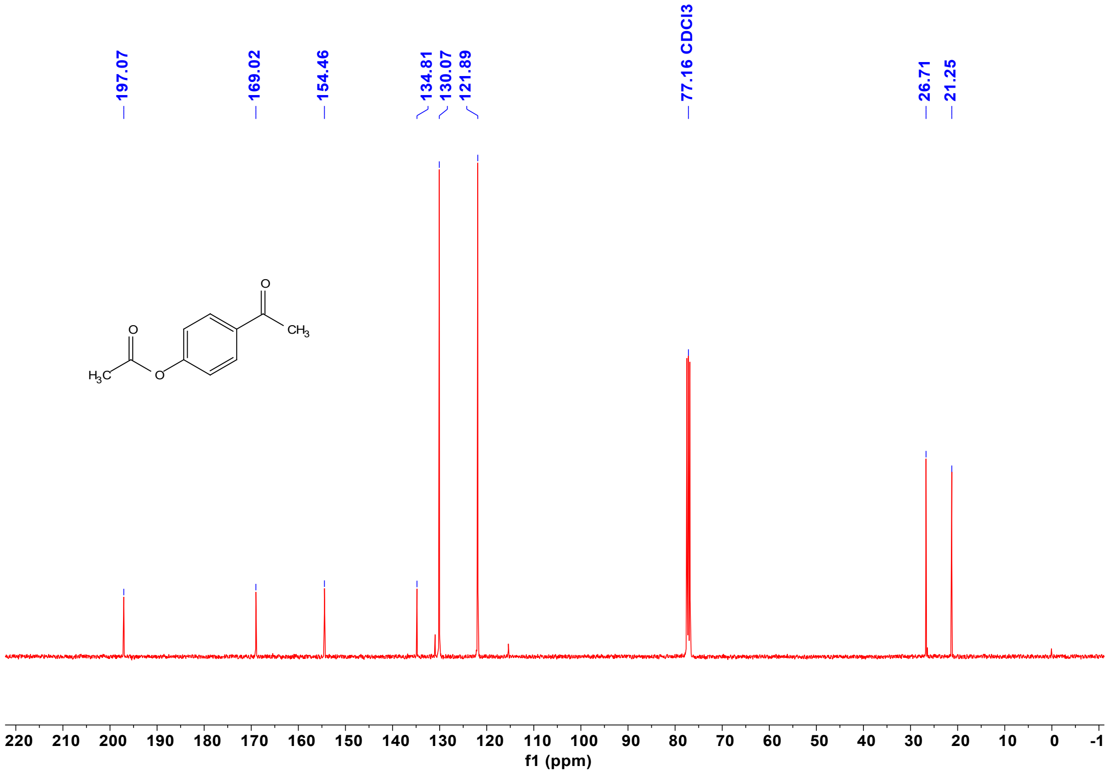
197.07
169.02
154.46
134.81
130.07
121.89
77.16 CDCl3
26.71
21.25
O
CH3
O
H3C
O
220 210 200 190 180 170 160 150 140 130 120 110 100 90 80 70 60 50 40 30 20 10 0 -1
f1 (ppm)

**2k**

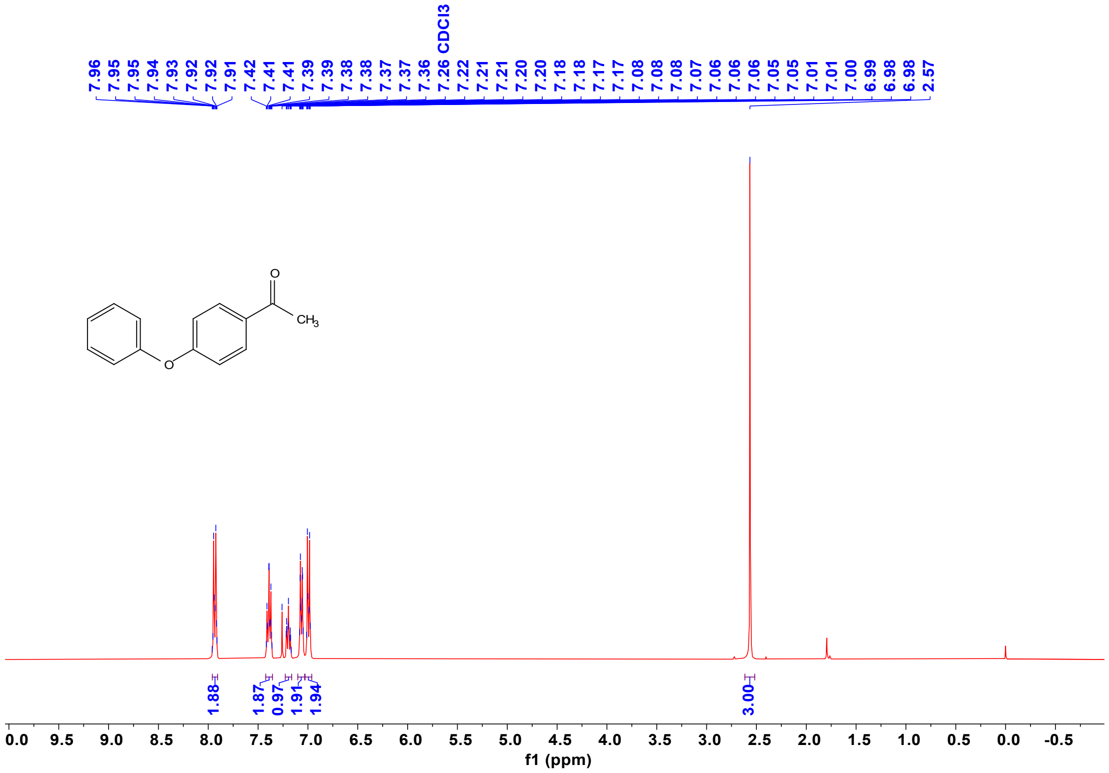
7.96
7.95
7.95
7.94
7.93
7.92
7.92
7.91
7.42
7.41
7.41
7.39
7.39
7.38
7.38
7.37
7.37
7.36
7.26 CDCl3
7.22
7.21
7.21
7.20
7.20
7.18
7.18
7.17
7.17
7.08
7.08
7.08
7.07
7.06
7.06
7.06
7.05
7.05
7.01
7.01
7.00
6.99
6.98
6.98
2.57
O
CH3
O
1.88
1.87
0.97
1.91
1.94
3.00
f1 (ppm)

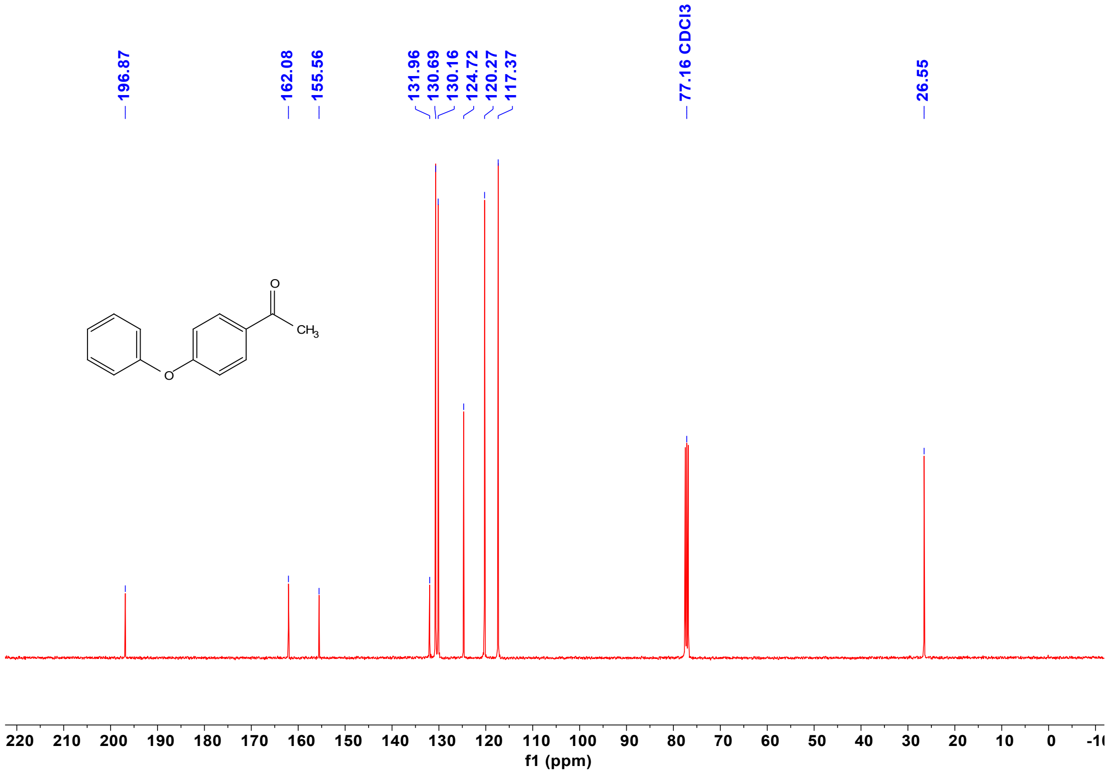
196.87
162.08
155.56
131.96
130.69
130.16
124.72
120.27
117.37
77.16 CDCl3
26.55
O
CH3
O
f1 (ppm)

**2l**

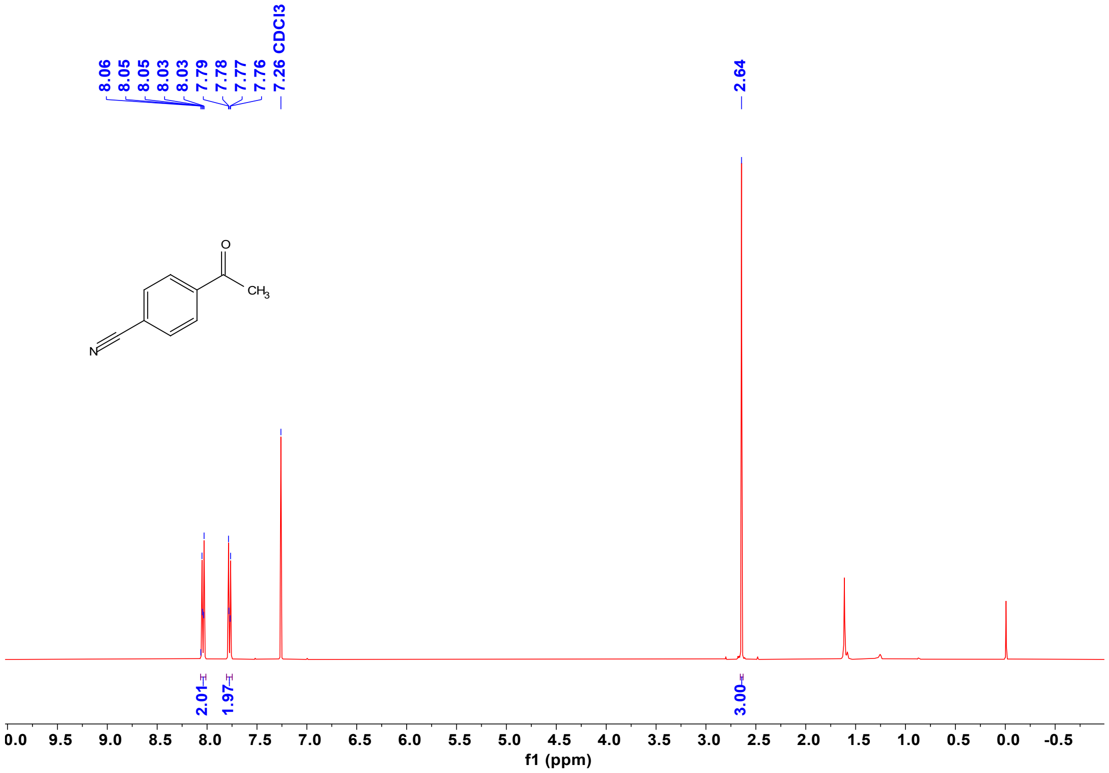
8.06
8.05
8.05
8.03
8.03
7.79
7.78
7.77
7.76
7.26 CDCl3
2.64
O
CH3
N
2.01
1.97
3.00
f1 (ppm)

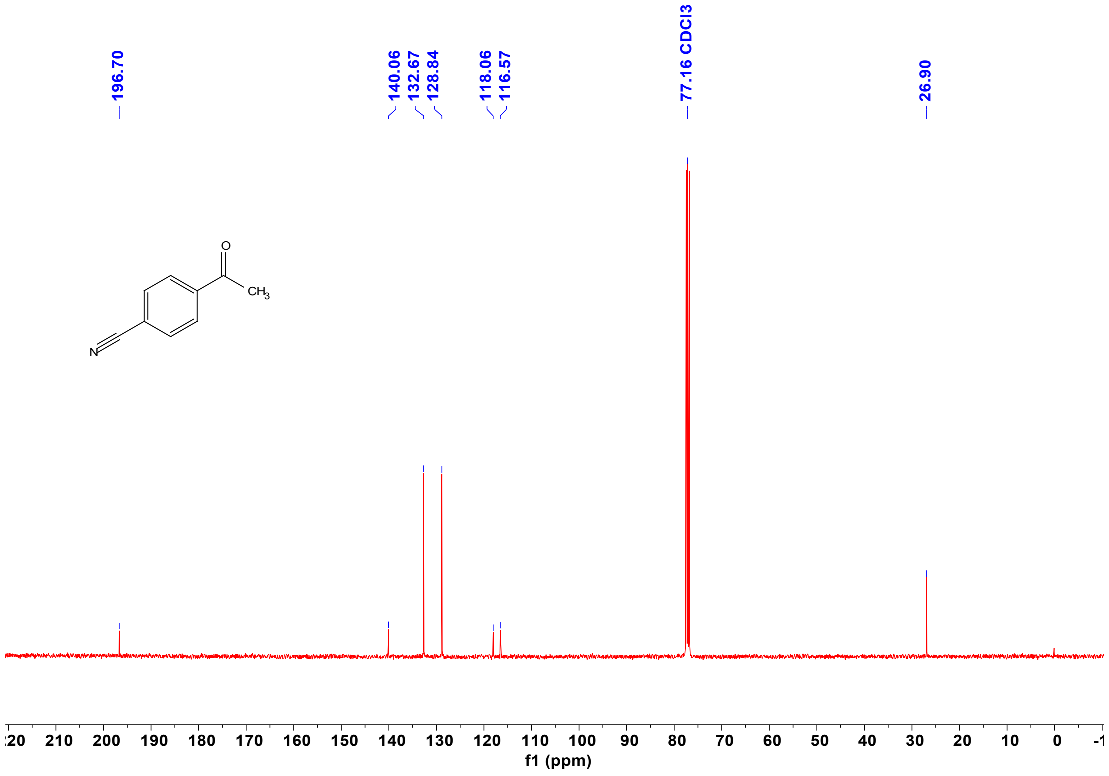
196.70
140.06
132.67
128.84
118.06
116.57
77.16 CDCl3
26.90
O
CH3
N
f1 (ppm)

**2m**

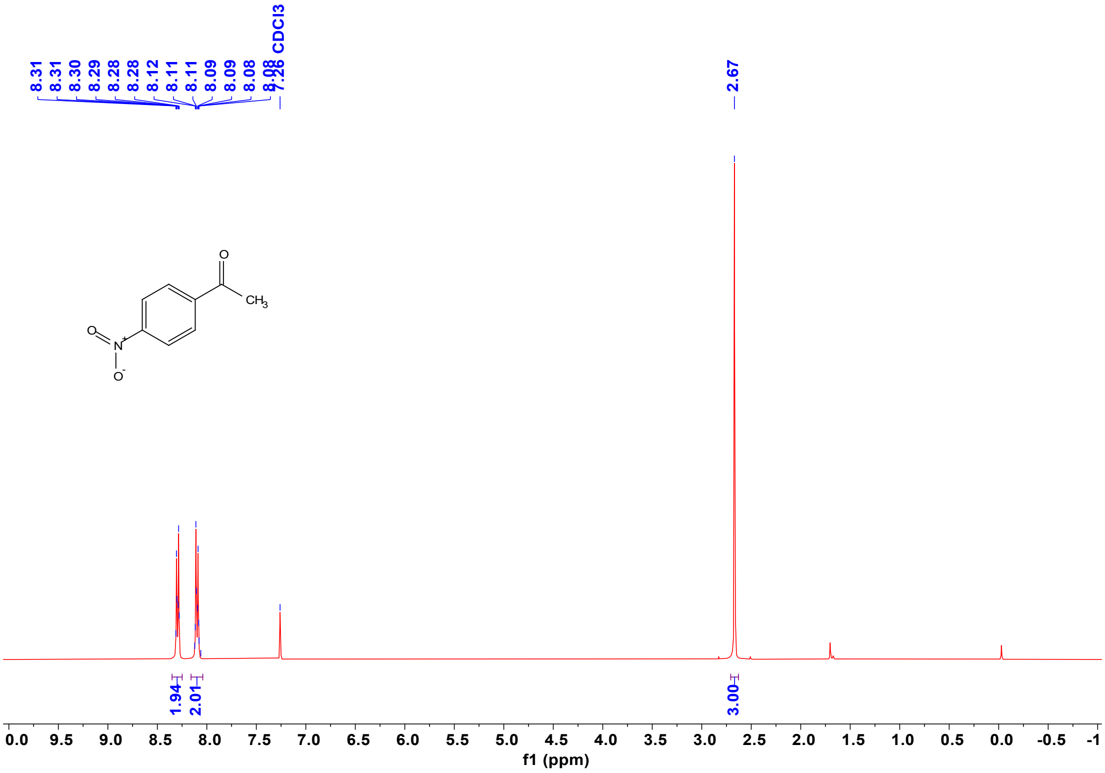
8.31
8.31
8.30
8.29
8.28
8.28
8.12
8.11
8.11
8.09
8.09
8.08
8.08
7.26 CDCl3
2.67
O
CH3
O
N+
O-
1.94
2.01
3.00
10.0 9.5 9.0 8.5 8.0 7.5 7.0 6.5 6.0 5.5 5.0 4.5 4.0 3.5 3.0 2.5 2.0 1.5 1.0 0.5 0.0 -0.5 -1
f1 (ppm)

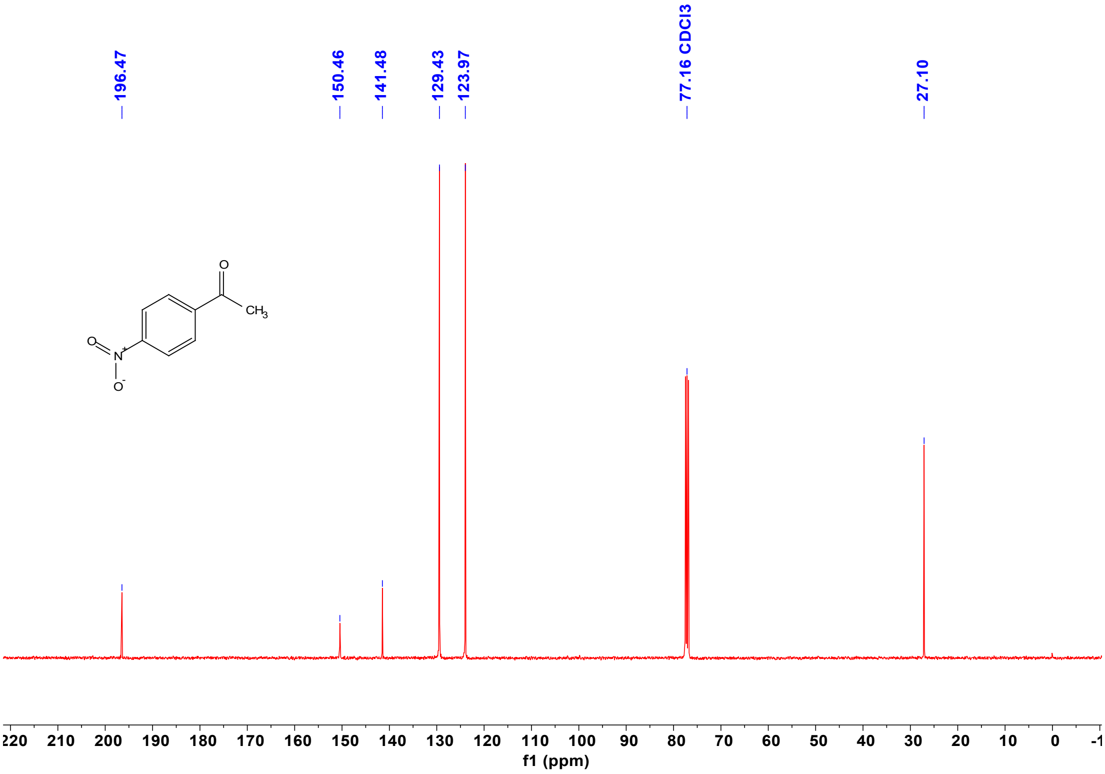
196.47
150.46
141.48
129.43
123.97
77.16 CDCl3
27.10
O
CH3
O
N+
O-
220 210 200 190 180 170 160 150 140 130 120 110 100 90 80 70 60 50 40 30 20 10 0 -1
f1 (ppm)

**2n**

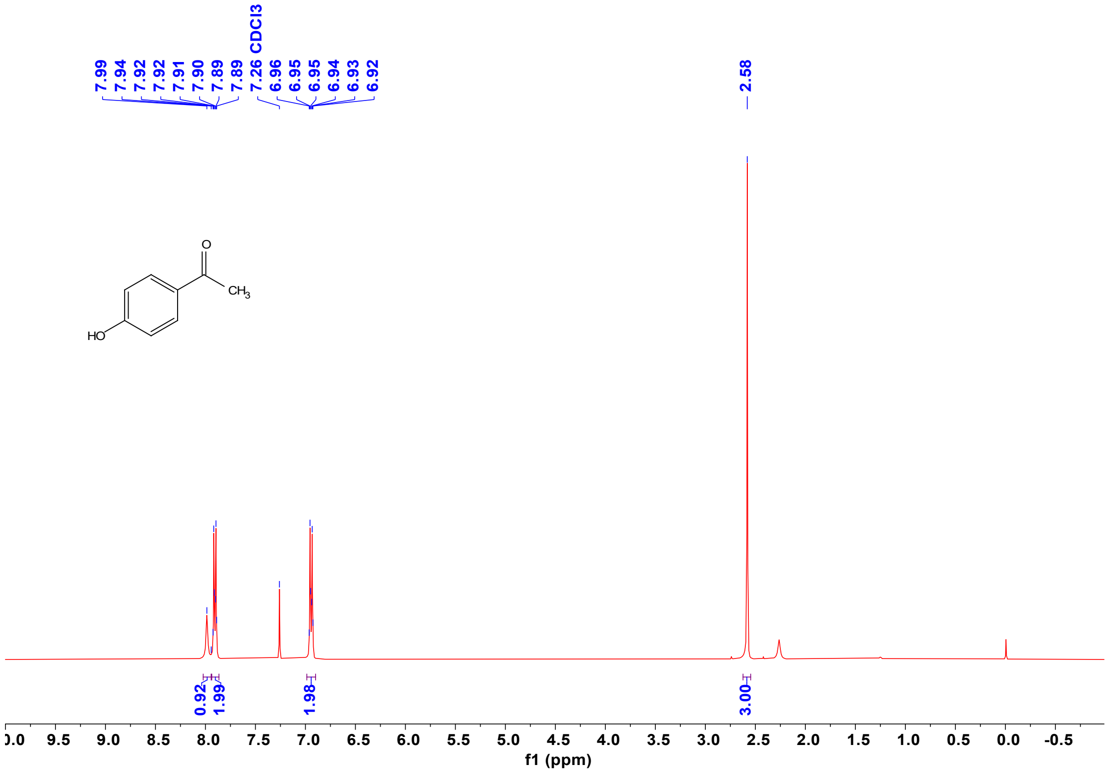

7.99
7.94
7.92
7.92
7.91
7.90
7.89
7.89
7.26 CDCl3
6.96
6.95
6.95
6.94
6.93
6.92
2.58
O
CH3
HO
0.92
1.99
1.98
3.00
f1 (ppm)


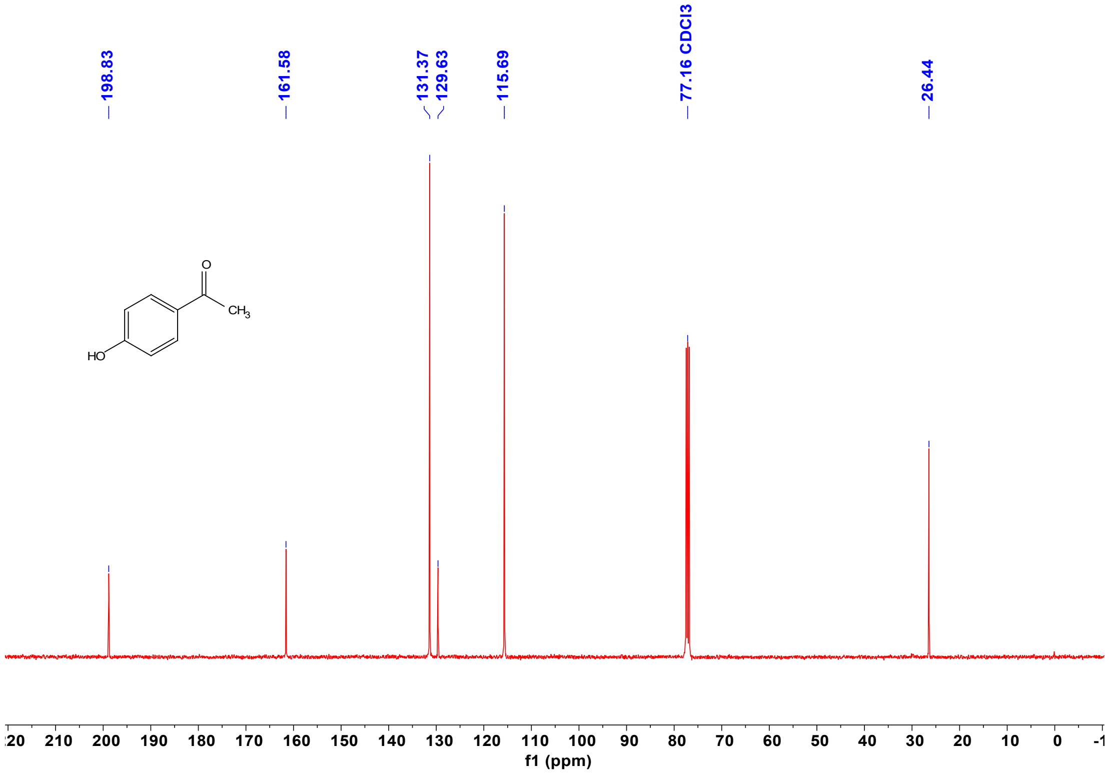

198.83
161.58
131.37
129.63
115.69
77.16 CDCl3
26.44
O
CH3
HO
f1 (ppm)

**2o**

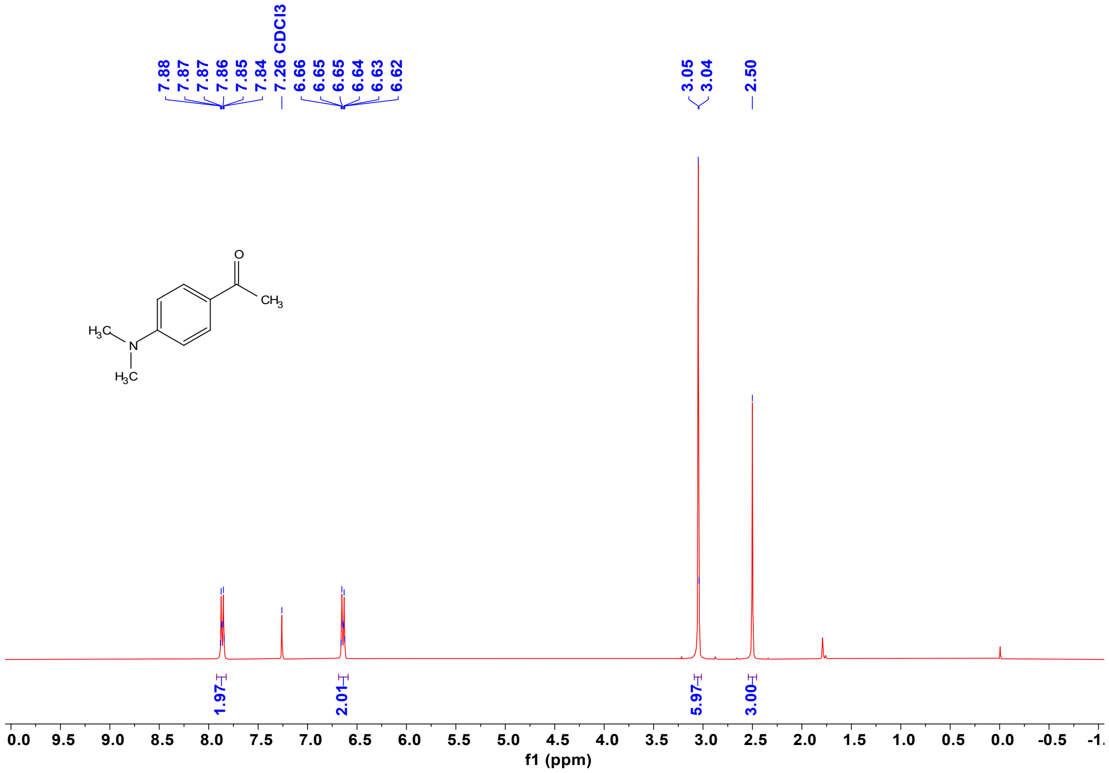

7.88
7.87
7.87
7.86
7.85
7.84
7.26 CDCl3
6.66
6.65
6.65
6.64
6.63
6.62
3.05
3.04
2.50
1.97
2.01
5.97
3.00
f1 (ppm)


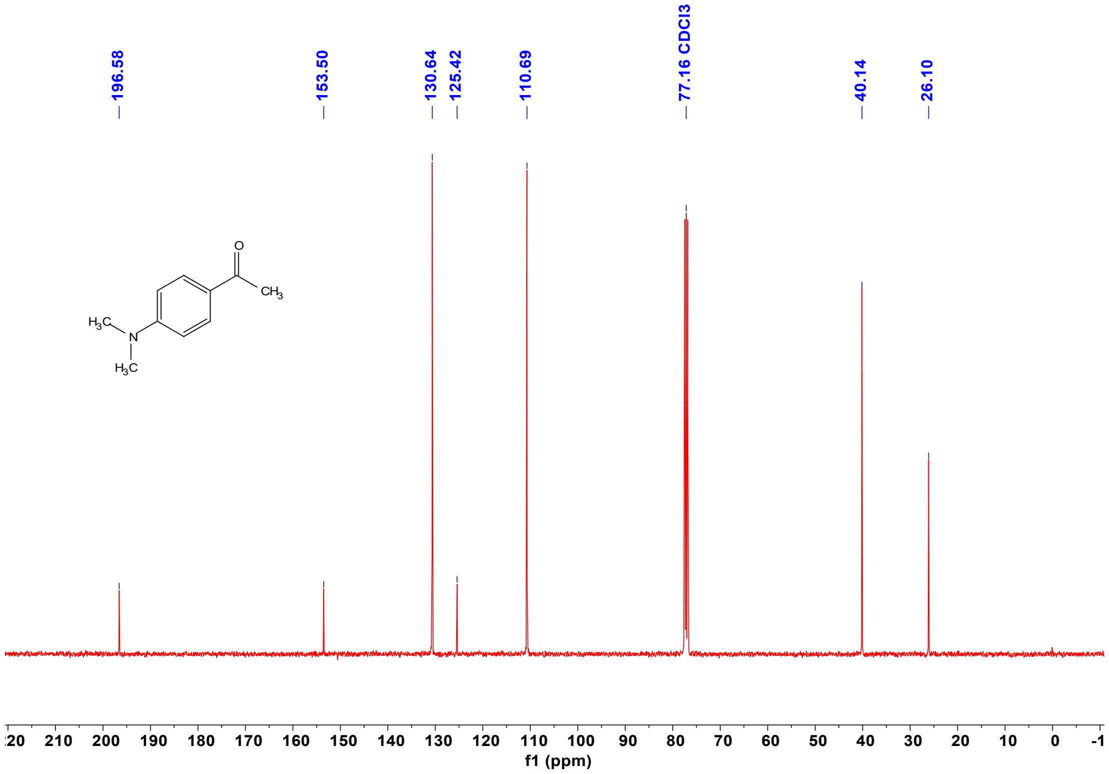

196.58
153.50
130.64
125.42
110.69
77.16 CDCl3
40.14
26.10
f1 (ppm)

# 2q

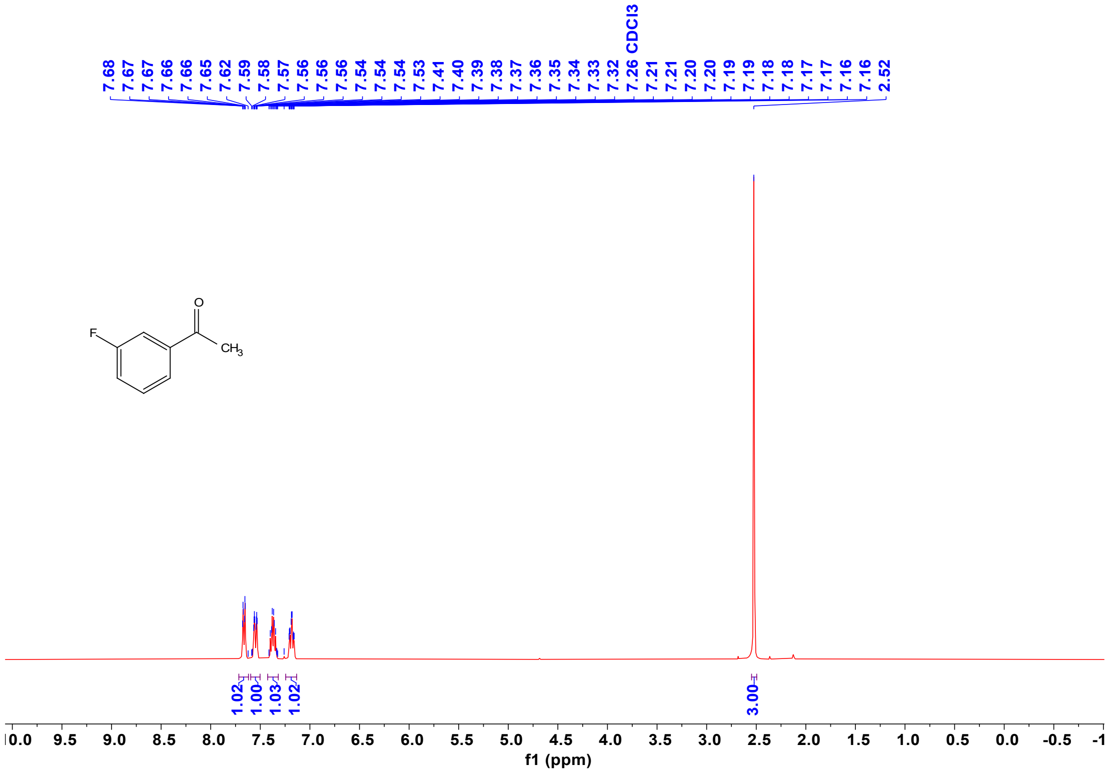

7.68 7.67 7.67 7.66 7.66 7.65 7.62 7.59 7.58 7.57 7.56 7.56 7.56 7.54 7.54 7.54 7.53 7.41 7.40 7.39 7.38 7.37 7.36 7.35 7.34 7.33 7.32 7.26 CDCl3 7.21 7.21 7.20 7.19 7.19 7.18 7.17 7.17 7.16 7.16 2.52
F
O
CH3
1.02
1.00
1.03
1.02
3.00
f1 (ppm)


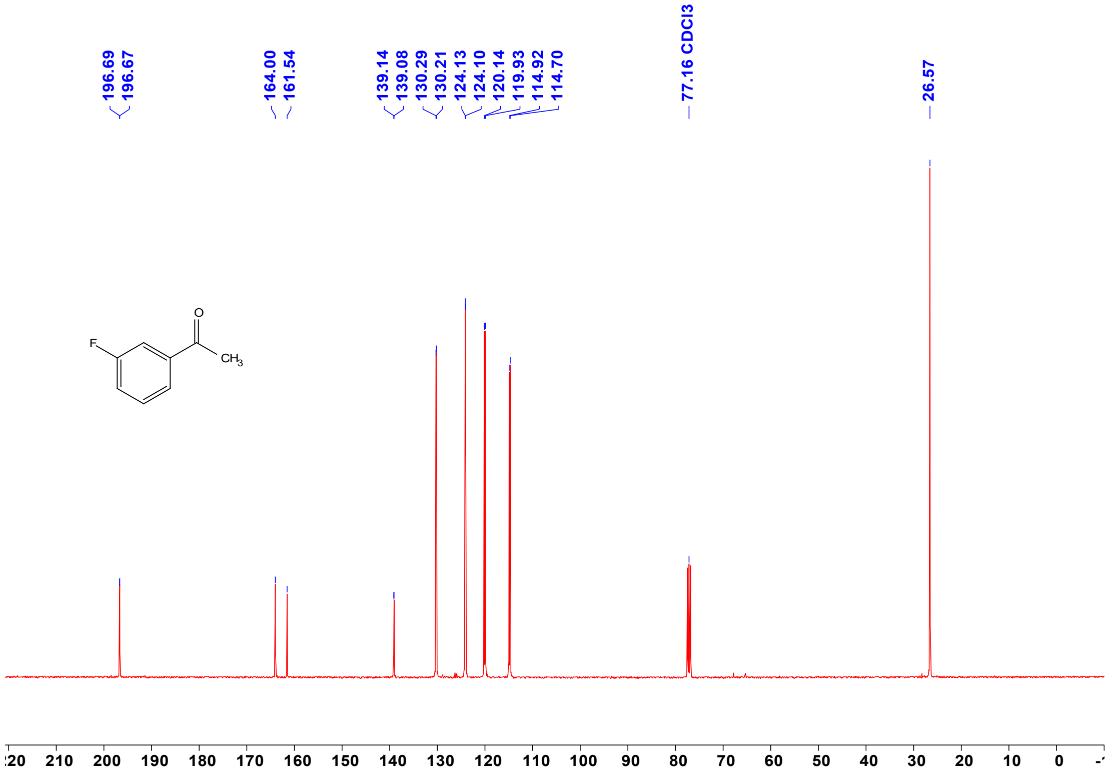

196.69 196.67
164.00 161.54
139.14 139.08 130.29 130.21 124.13 124.10 120.14 119.93 114.92 114.70
77.16 CDCl3
26.57
F
O
CH3

**2r**

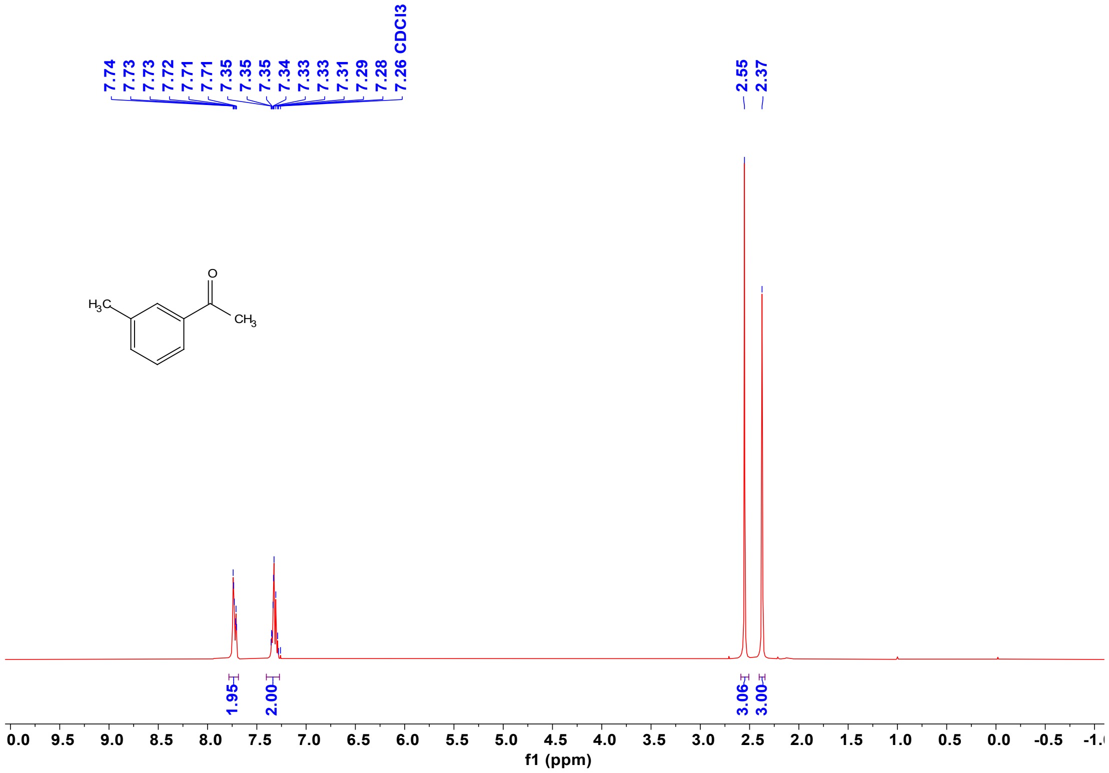
7.74
7.73
7.73
7.72
7.71
7.71
7.35
7.35
7.35
7.34
7.33
7.33
7.31
7.29
7.28
7.26 CDCl3
2.55
2.37
H3C
O
CH3
1.95
2.00
3.06
3.00
f1 (ppm)

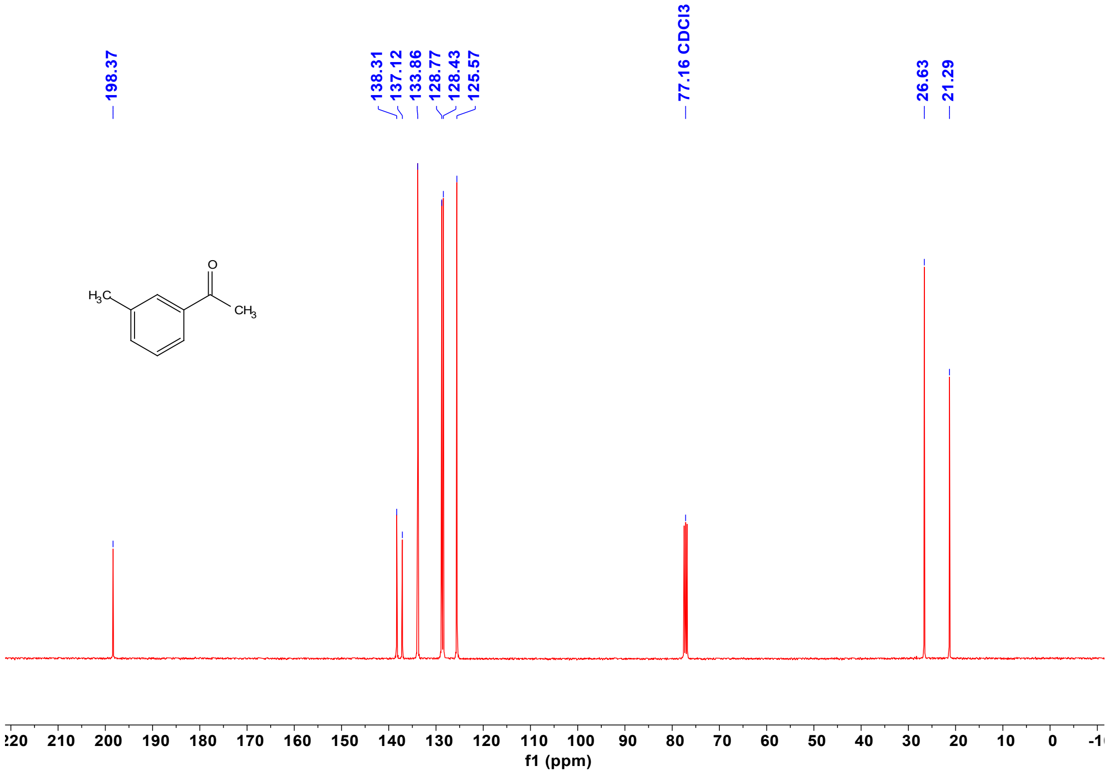
198.37
138.31
137.12
133.86
128.77
128.43
125.57
77.16 CDCl3
26.63
21.29
H3C
O
CH3
f1 (ppm)

**2s**

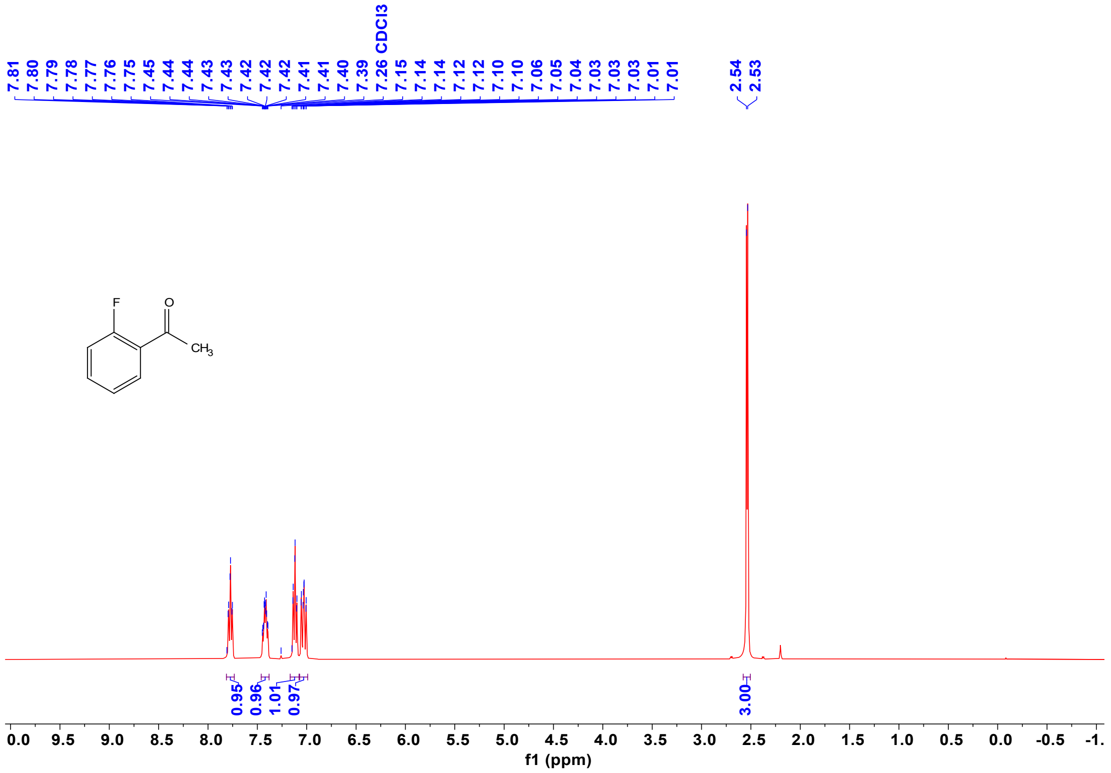

7.81
7.80
7.79
7.78
7.77
7.76
7.75
7.45
7.44
7.44
7.43
7.43
7.42
7.42
7.42
7.41
7.41
7.40
7.39
7.26 CDCl3
7.15
7.14
7.14
7.12
7.12
7.10
7.10
7.06
7.05
7.04
7.03
7.03
7.03
7.01
7.01
2.54
2.53
F
O
CH3
0.95
0.96
1.01
0.97
3.00
f1 (ppm)


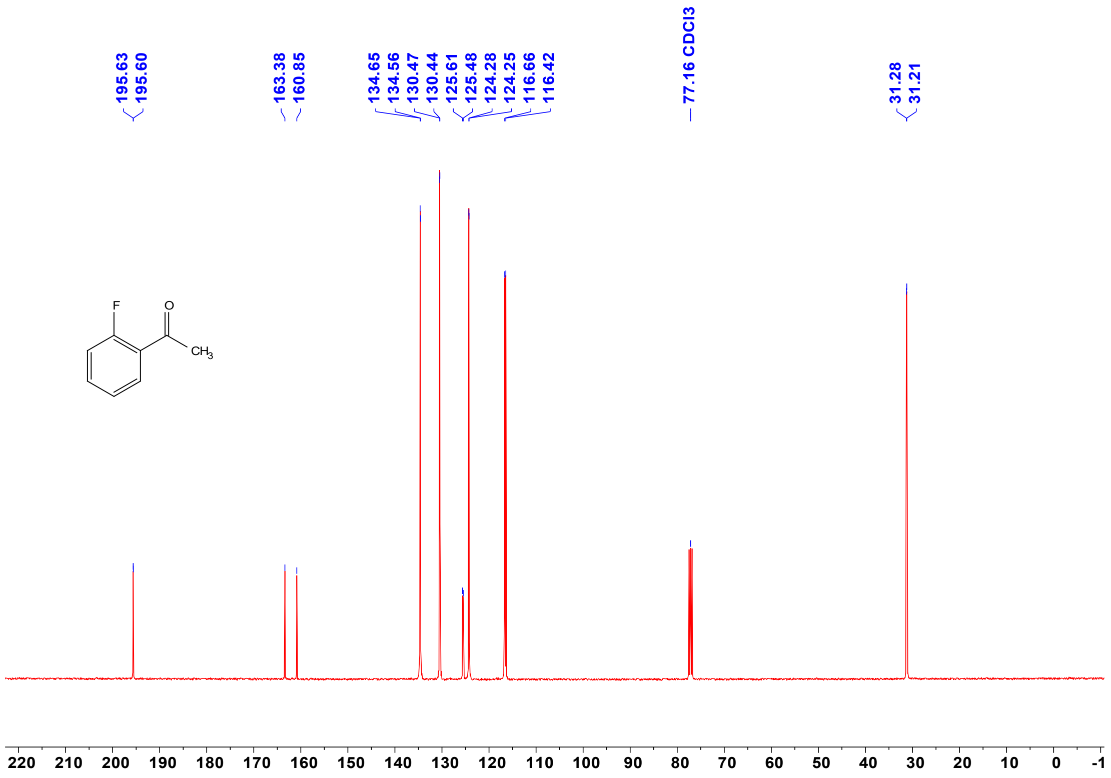

195.63
195.60
163.38
160.85
134.65
134.56
130.47
130.44
125.61
125.48
124.28
124.25
116.66
116.42
77.16 CDCl3
31.28
31.21
F
O
CH3

**2t**

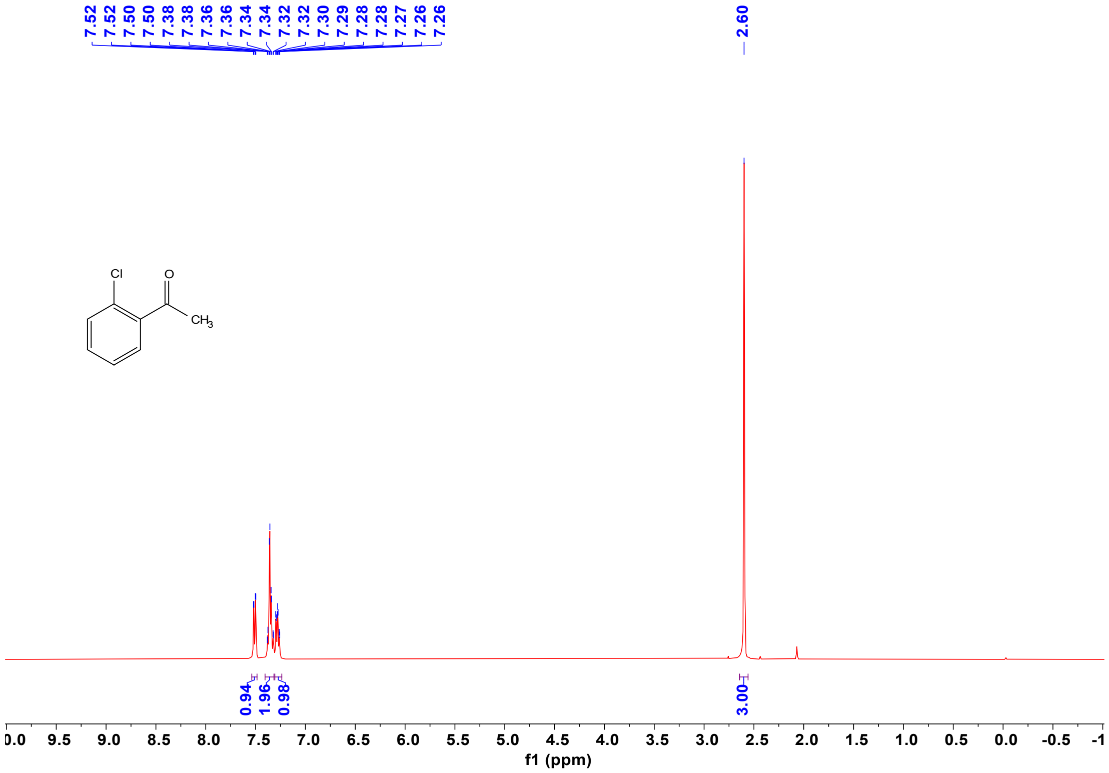

7.52
7.52
7.50
7.50
7.38
7.38
7.36
7.36
7.34
7.34
7.32
7.32
7.30
7.29
7.28
7.28
7.27
7.26
7.26
2.60
Cl
O
CH3
0.94
1.96
0.98
3.00
f1 (ppm)


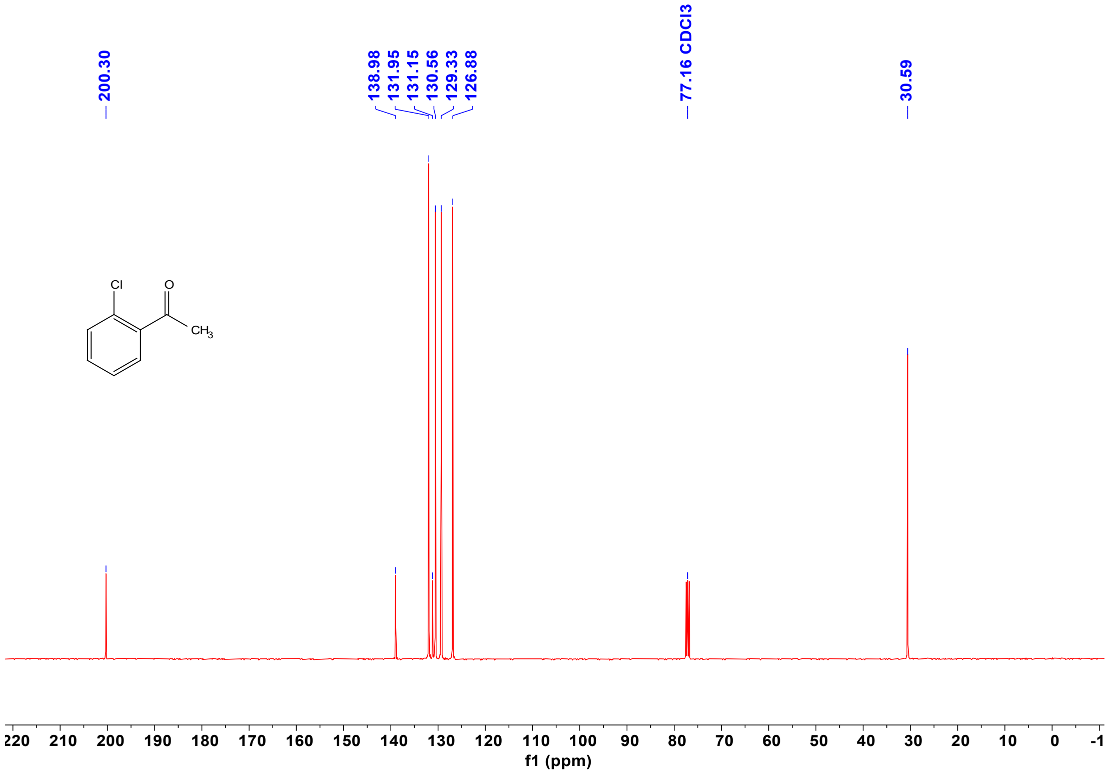

200.30
138.98
131.95
131.15
130.56
129.33
126.88
77.16 CDCl3
30.59
Cl
O
CH3
f1 (ppm)

## 2u

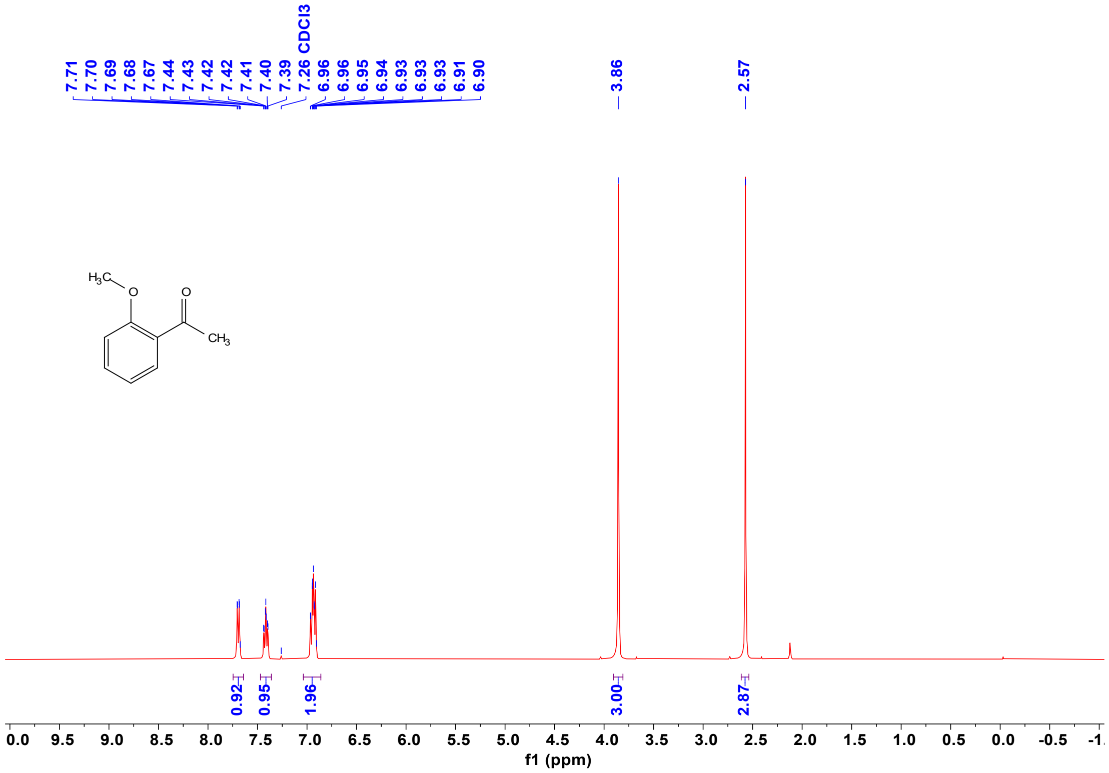
7.71
7.70
7.69
7.68
7.67
7.44
7.43
7.42
7.42
7.41
7.40
7.39
7.26 CDCl3
6.96
6.96
6.95
6.94
6.93
6.93
6.93
6.91
6.90
3.86
2.57
0.92
0.95
1.96
3.00
2.87
f1 (ppm)

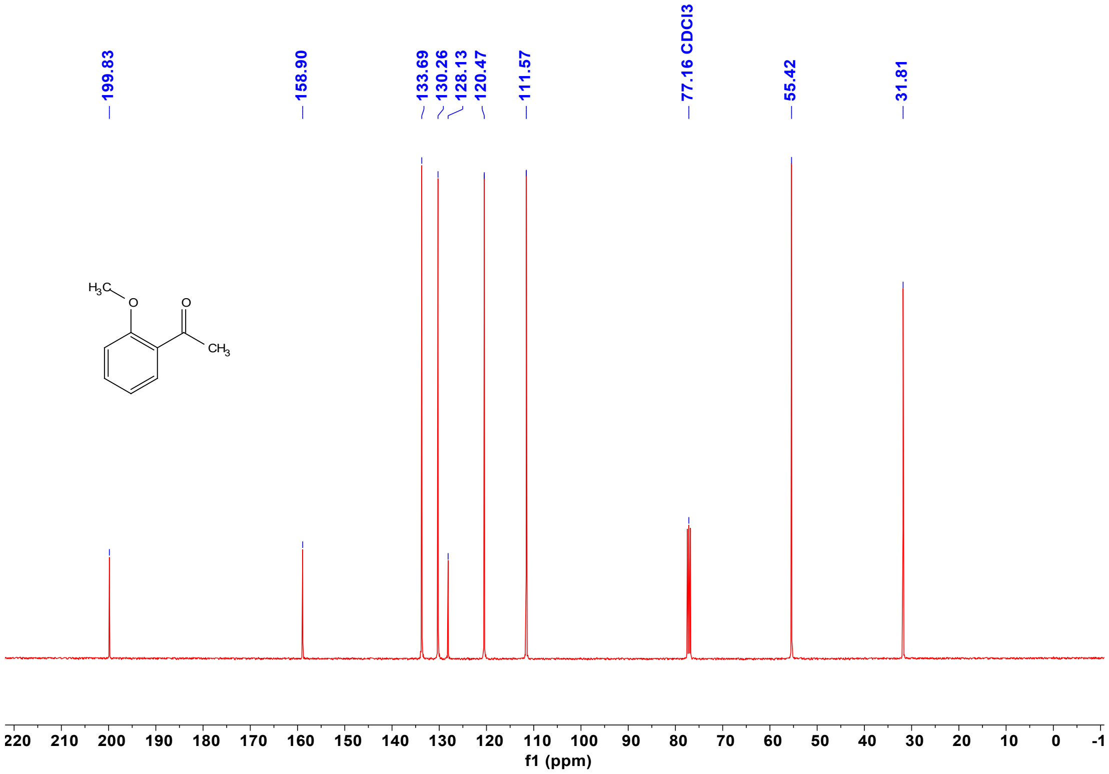
199.83
158.90
133.69
130.26
128.13
120.47
111.57
77.16 CDCl3
55.42
31.81
f1 (ppm)

**2w**

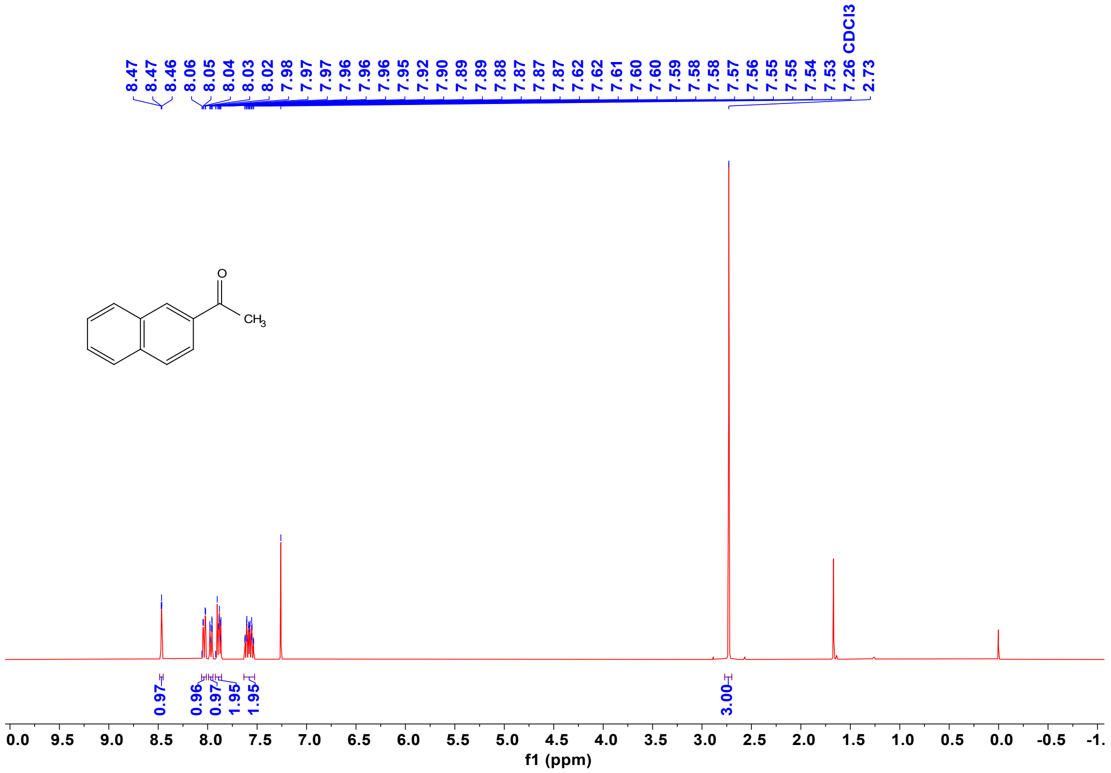

8.47
8.47
8.46
8.06
8.05
8.04
8.03
8.02
7.98
7.97
7.97
7.96
7.96
7.96
7.95
7.92
7.90
7.89
7.89
7.88
7.87
7.87
7.87
7.62
7.62
7.61
7.60
7.60
7.59
7.58
7.58
7.57
7.56
7.55
7.55
7.54
7.53
7.26 CDCl3
2.73
O
CH3
0.97
0.96
0.97
1.95
1.95
3.00
0.0 9.5 9.0 8.5 8.0 7.5 7.0 6.5 6.0 5.5 5.0 4.5 4.0 3.5 3.0 2.5 2.0 1.5 1.0 0.5 0.0 -0.5 -1.
f1 (ppm)


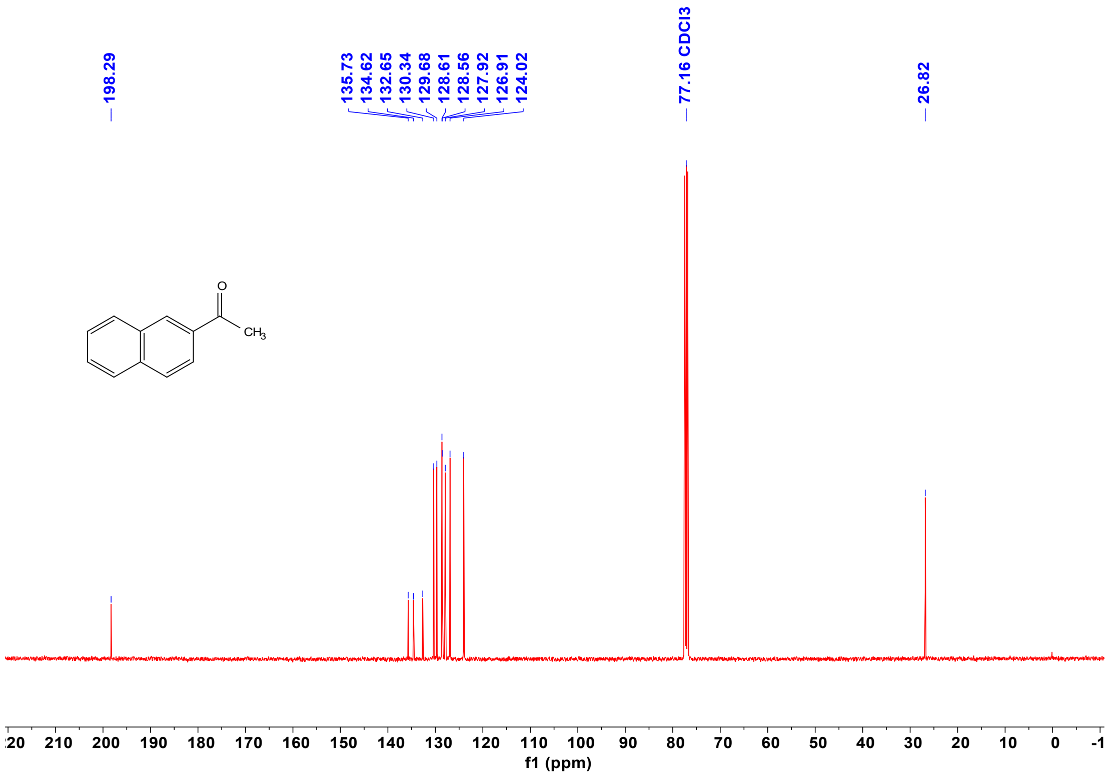

198.29
135.73
134.62
132.65
130.34
129.68
128.61
128.56
127.92
126.91
124.02
77.16 CDCl3
26.82
O
CH3
20 210 200 190 180 170 160 150 140 130 120 110 100 90 80 70 60 50 40 30 20 10 0 -1
f1 (ppm)

# 2x

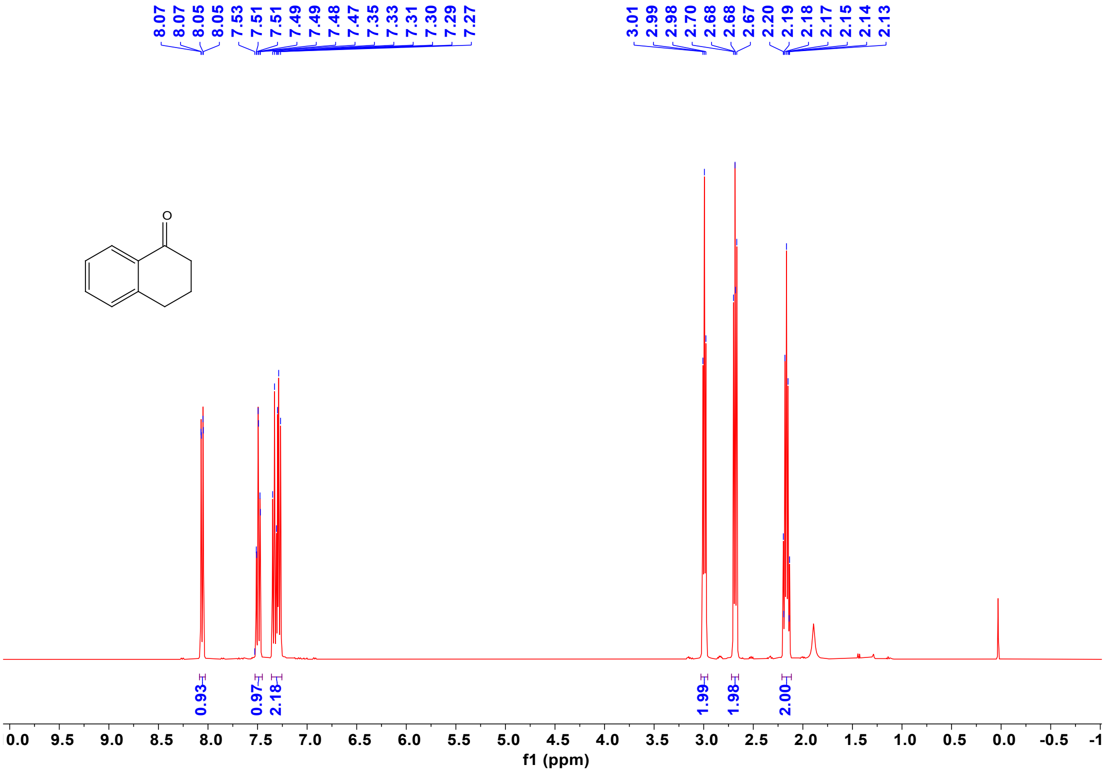

8.07 8.07 8.05 8.05 7.53 7.51 7.51 7.49 7.49 7.48 7.47 7.35 7.33 7.31 7.30 7.29 7.27
3.01 2.99 2.98 2.70 2.68 2.68 2.67 2.20 2.19 2.18 2.17 2.15 2.14 2.13
O
0.93
0.97
2.18
1.99
1.98
2.00
0.0 9.5 9.0 8.5 8.0 7.5 7.0 6.5 6.0 5.5 5.0 4.5 4.0 3.5 3.0 2.5 2.0 1.5 1.0 0.5 0.0 -0.5 -1
f1 (ppm)


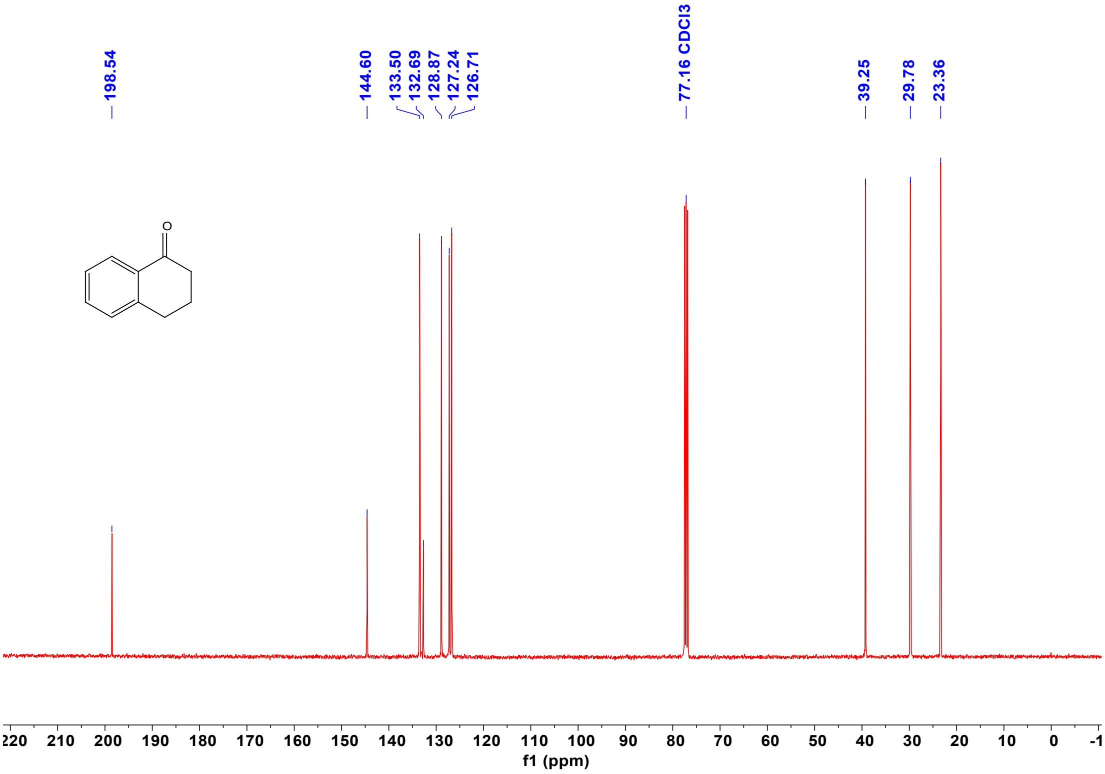

198.54
144.60
133.50
132.69
128.87
127.24
126.71
77.16 CDCl3
39.25
29.78
23.36
O
220 210 200 190 180 170 160 150 140 130 120 110 100 90 80 70 60 50 40 30 20 10 0 -1
f1 (ppm)

**2y**

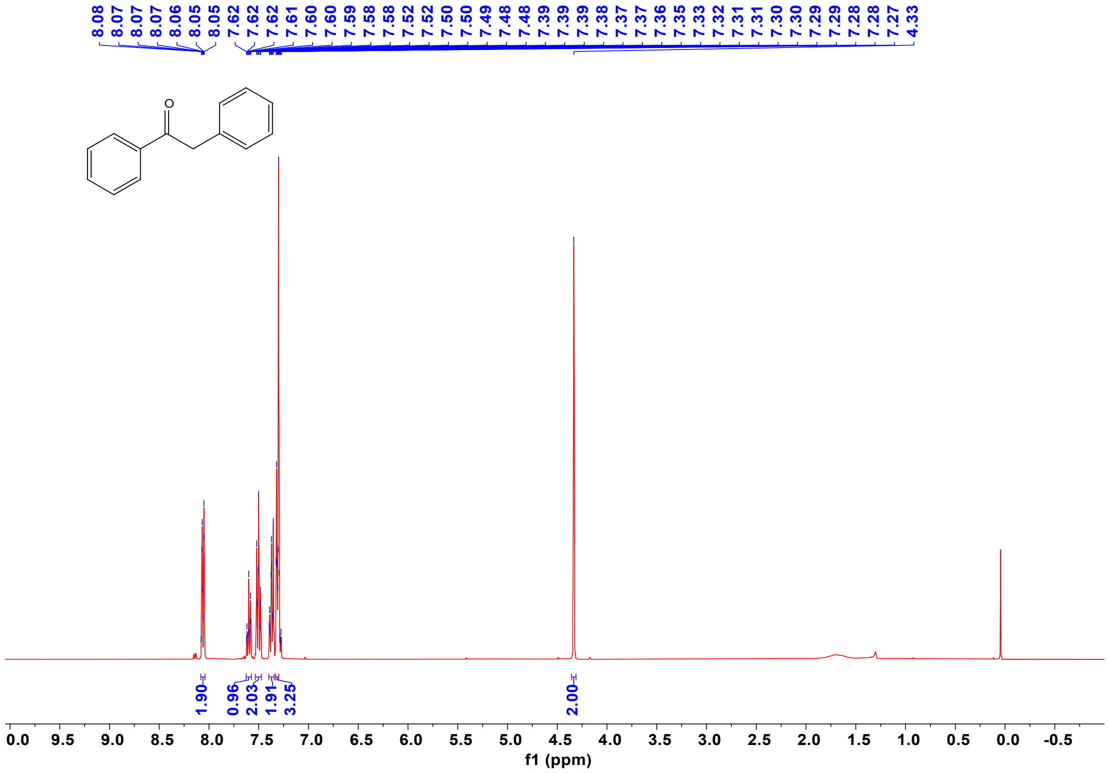
8.08
8.07
8.07
8.06
8.05
8.05
7.62
7.62
7.62
7.61
7.60
7.59
7.58
7.58
7.52
7.52
7.50
7.50
7.49
7.48
7.48
7.39
7.39
7.39
7.38
7.37
7.37
7.36
7.35
7.33
7.32
7.31
7.31
7.30
7.30
7.29
7.29
7.28
7.28
7.27
4.33
O
1.90
0.96
2.03
1.91
3.25
2.00
f1 (ppm)

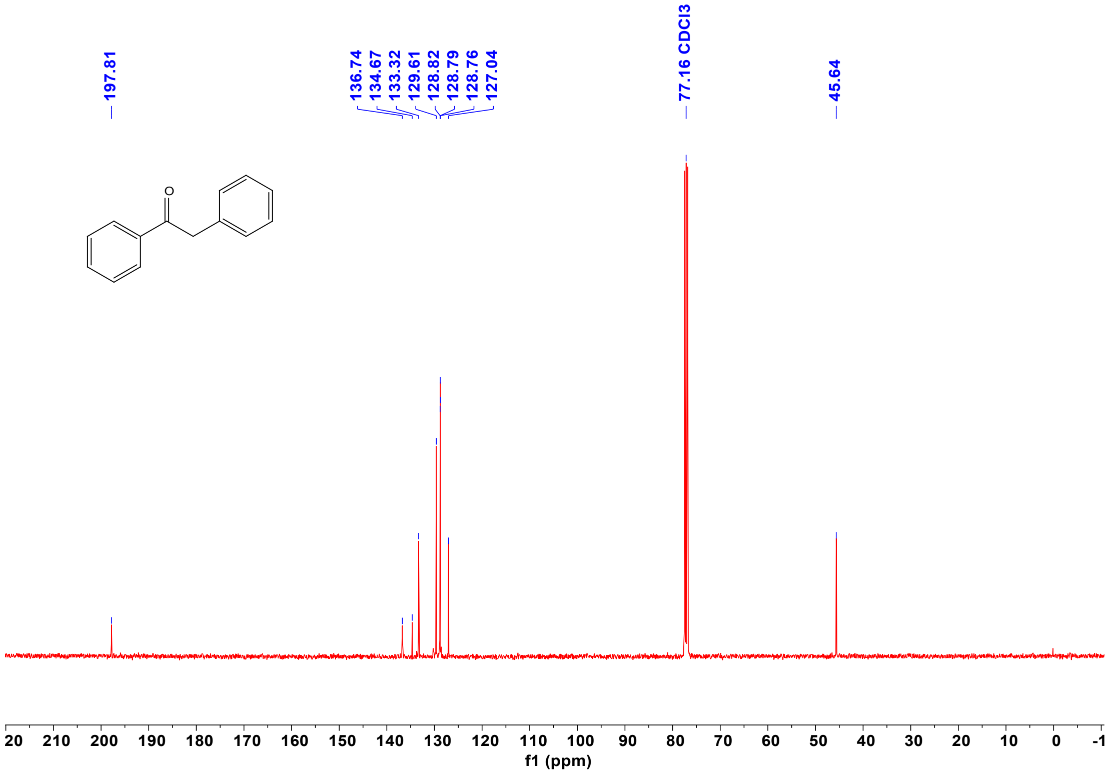
197.81
136.74
134.67
133.32
129.61
128.82
128.79
128.76
127.04
77.16 CDCl3
45.64
O
f1 (ppm)

# 2aa

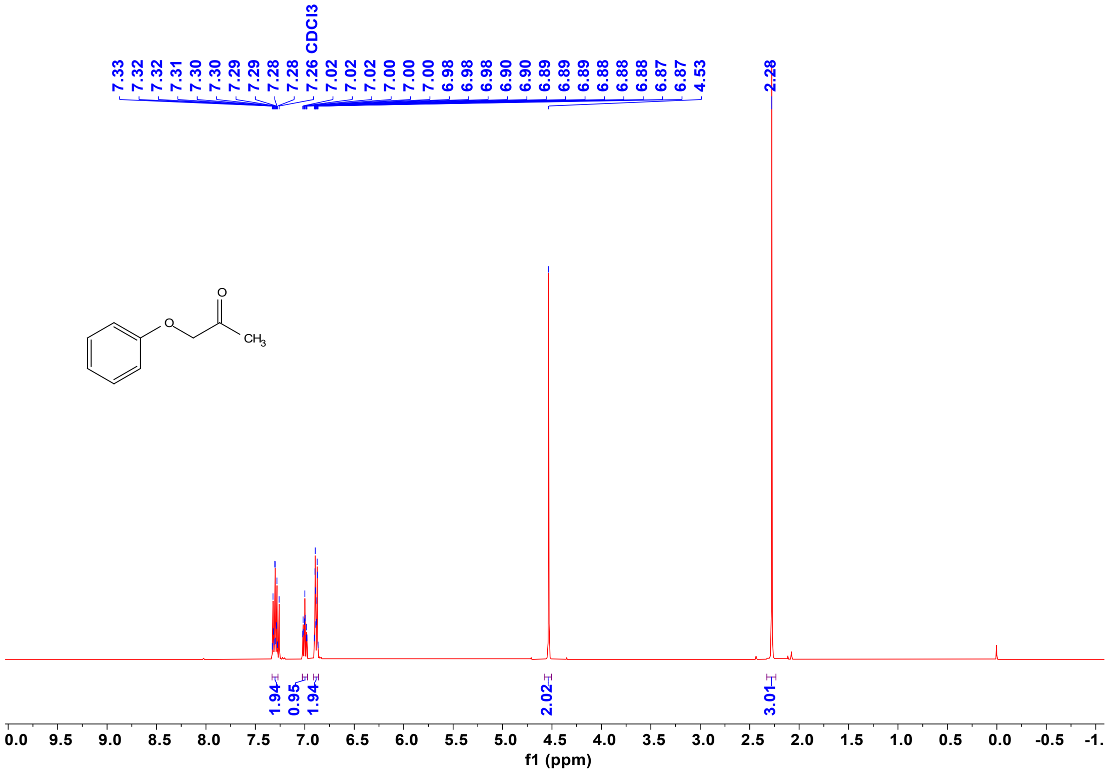

7.33
7.32
7.32
7.31
7.30
7.30
7.29
7.29
7.28
7.28
7.26 CDCl3
7.02
7.02
7.02
7.00
7.00
6.98
6.98
6.98
6.90
6.90
6.89
6.89
6.89
6.88
6.88
6.87
6.87
4.53
2.28
O
CH3
1.94
0.95
1.94
2.02
3.01
f1 (ppm)


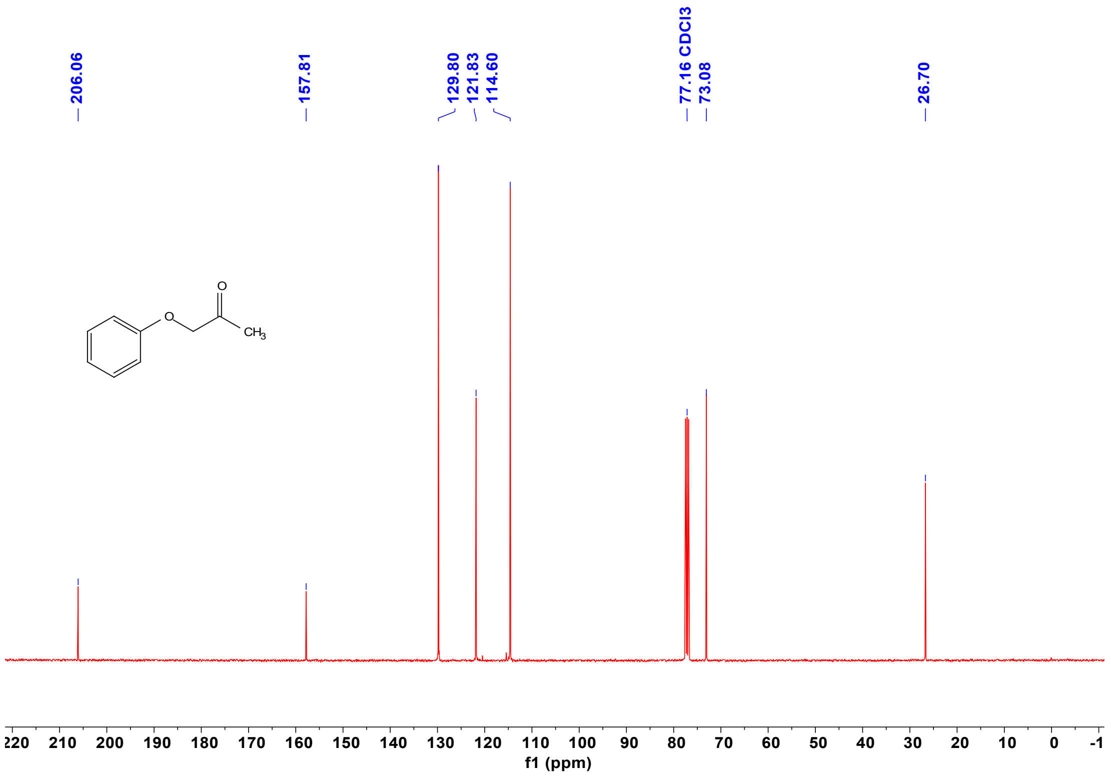

206.06
157.81
129.80
121.83
114.60
77.16 CDCl3
73.08
26.70
O
CH3
f1 (ppm)

# IN1

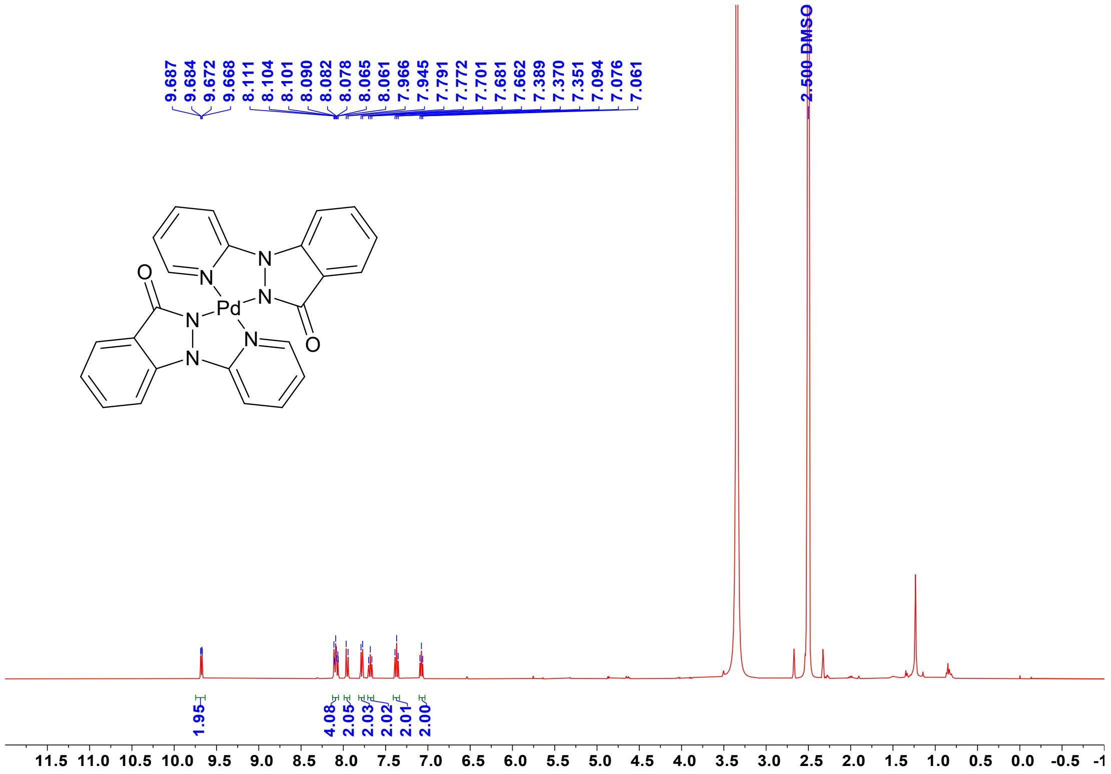
9.687 9.684 9.672 9.668 8.111 8.104 8.101 8.090 8.082 8.078 8.065 8.061 7.966 7.945 7.791 7.772 7.701 7.681 7.662 7.389 7.370 7.351 7.094 7.076 7.061
2.500 DMSO
1.95 4.08 2.05 2.03 2.02 2.01 2.00
O N Pd N N N N O
11.5 11.0 10.5 10.0 9.5 9.0 8.5 8.0 7.5 7.0 6.5 6.0 5.5 5.0 4.5 4.0 3.5 3.0 2.5 2.0 1.5 1.0 0.5 0.0 -0.5 -1

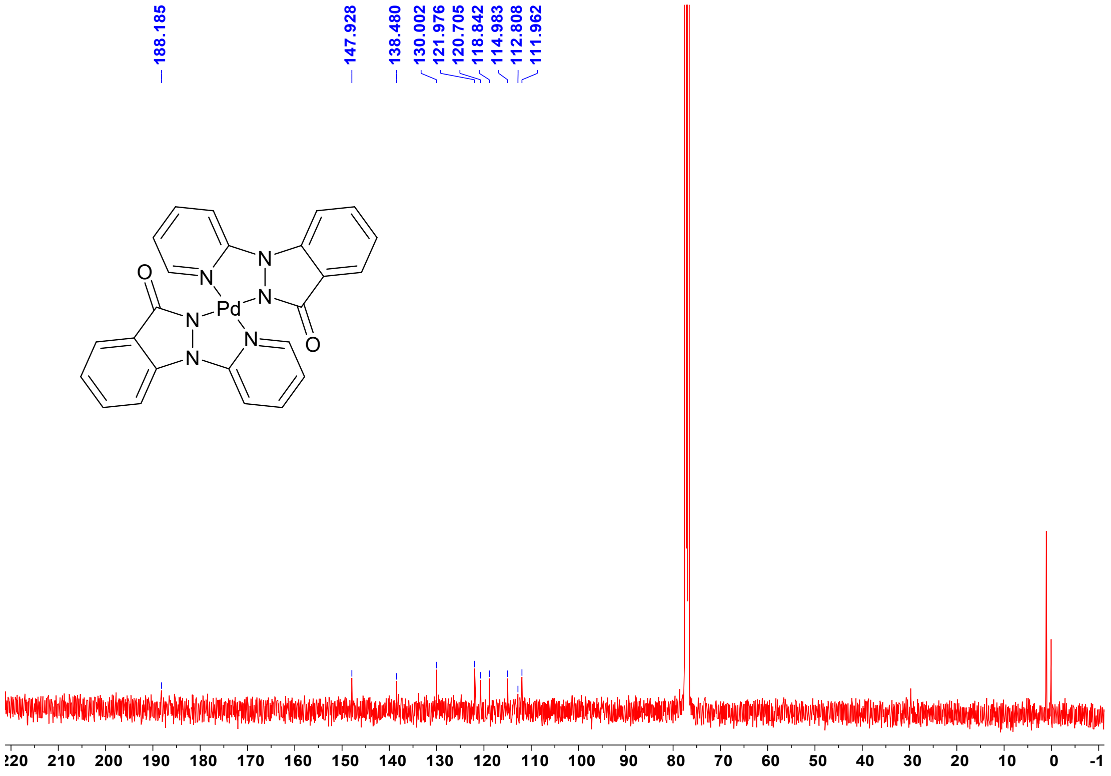
188.185
147.928
138.480
130.002 121.976 120.705 118.842 114.983 112.808 111.962
O N Pd N N N N O
220 210 200 190 180 170 160 150 140 130 120 110 100 90 80 70 60 50 40 30 20 10 0 -1

## IN2

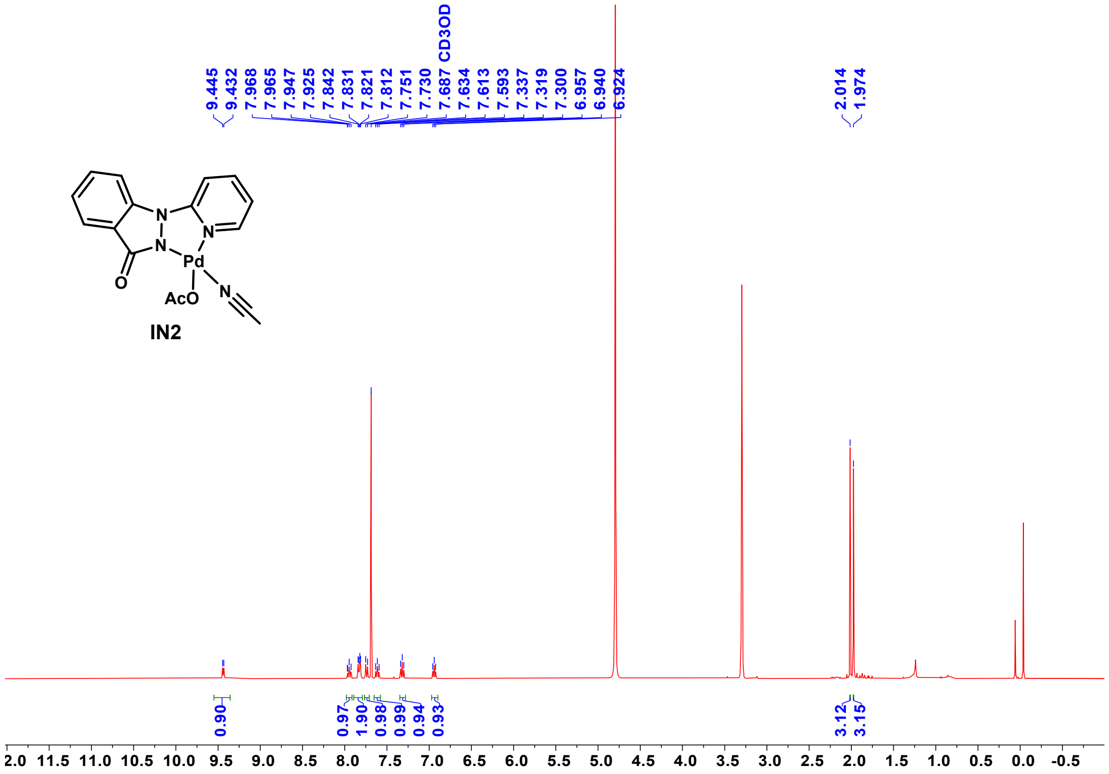

9.445
9.432
7.968
7.965
7.947
7.925
7.842
7.831
7.821
7.812
7.751
7.730
7.687 CD3OD
7.634
7.613
7.593
7.337
7.319
7.300
6.957
6.940
6.924
2.014
1.974
Pd
AcO
IN2
0.90
0.97
1.90
0.98
0.99
0.94
0.93
3.12
3.15
2.0 11.5 11.0 10.5 10.0 9.5 9.0 8.5 8.0 7.5 7.0 6.5 6.0 5.5 5.0 4.5 4.0 3.5 3.0 2.5 2.0 1.5 1.0 0.5 0.0 -0.5


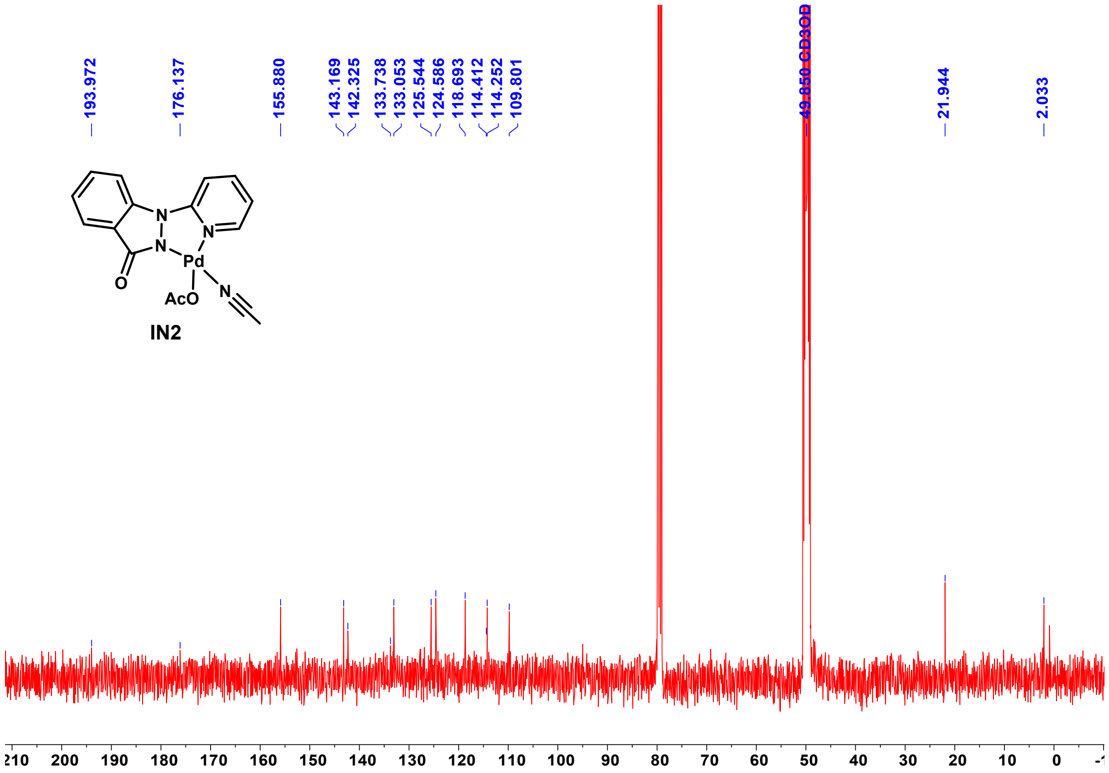

193.972
176.137
155.880
143.169
142.325
133.738
133.053
125.544
124.586
118.693
114.412
114.252
109.801
49.850 CD3OD
21.944
2.033
Pd
AcO
IN2
210 200 190 180 170 160 150 140 130 120 110 100 90 80 70 60 50 40 30 20 10 0
